# Adult Bone Marrow Neural Crest Stem Cells and Mesenchymal Stem Cells Are Not Able to Replace Lost Neurons in Acute MPTP-Lesioned Mice

**DOI:** 10.1371/journal.pone.0064723

**Published:** 2013-05-31

**Authors:** Virginie Neirinckx, Alice Marquet, Cécile Coste, Bernard Rogister, Sabine Wislet-Gendebien

**Affiliations:** 1 Groupe Interdisciplinaire de Génoprotéomique Appliquée (GIGA), Unit of Neurosciences, University of Liege, Liège, Belgium; 2 GIGA, Unit of Development, Stem Cells and Regenerative Medicine, University of Liège, Liège, Belgium; 3 Department of Neurology, Centre Hospitalier Universitaire de Liège, Liège, Belgium; Universidade Federal do ABC, Brazil

## Abstract

Adult bone marrow stroma contains multipotent stem cells (BMSC) that are a mixed population of mesenchymal and neural-crest derived stem cells. Both cells are endowed with *in vitro* multi-lineage differentiation abilities, then constituting an attractive and easy-available source of material for cell therapy in neurological disorders. Whereas the *in vivo* integration and differentiation of BMSC in neurons into the central nervous system is currently matter of debate, we report here that once injected into the striatum of 1-methyl-4-phenyl-1,2,3,6-tetrahydropyridine (MPTP)-treated mice, pure populations of either bone marrow neural crest stem cells (NCSC) or mesenchymal stem cells (MSC) survived only transiently into the lesioned brain. Moreover, they do not migrate through the brain tissue, neither modify their initial phenotype, while no recovery of the dopaminergic system integrity was observed. Consequently, we tend to conclude that MSC/NCSC are not able to replace lost neurons in acute MPTP-lesioned dopaminergic system through a suitable integration and/or differentiation process. Altogether with recent data, it appears that neuroprotective, neurotrophic and anti-inflammatory features characterizing BMSC are of greater interest as regards CNS lesions management.

## Introduction

The treatment of neurological disorders represents a critical issue in clinical research, since no complete functional recovery can be achieved with current therapeutic means, despite symptomatic improvements. Indeed, whereas restricted brain areas still house cells competent to generate newborn neurons in adulthood [1], [2], this limited neurogenesis does not seem to be sufficient to enable neuronal regeneration in cases of lesions of the central nervous system. Therefore, other sources of neural cells have to be considered in a cell therapy objective. Stem cells are characterized as cells endowed with continuous self-renewal ability and pluri- or multipotentiality [3], and could consequently give rise to a wide panel of cell types, including neural cells. Indeed, while neurons have already been successfully generated from embryonic stem cells (ES) [4], [5] or induced pluripotent stem cells (iPS) [6], [7], the use of adult somatic stem cells definitely remains of significant interest regarding technical, ethical and immunological issues concerning cell transplantation for brain-related diseases. In this regard, bone marrow stromal cells (BMSC) represent an important source of easily-accessible multipotent cells to use in a cell therapy purpose [8].

Numerous studies already described cell therapy experiments using BMSC and explored their neuronal plasticity *in vivo* [9]–[11]. However, result discrepancies appeared in those studies, which could mainly reside in the lack of exact phenotypic characterization of BMSC, due to the absence of specific membrane markers and non-standardized culture methods. Consequently, several groups described BMSC with major different phenotypes: Verfaillie’s group described a rare population of cells in human BM stroma as mesodermal adult progenitor cells [12], [13]; D’Ippolito and collaborators cultured cells in low oxygen tension and characterized marrow isolated adult multilineage inducible cells [14], [15]; whereas a lot of other groups kept the mesenchymal stem cell (MSC) concept as defined by Pittenger et al. [16]. In addition to the phenotypic differences of BMSC which are inherent to culture settings, it has been demonstrated that BMSC are constituted by a mixed population of cells arising from different embryonic lineages. Indeed, although adult BMSC were commonly considered to be of mesodermal origin [17], several studies have conclusively shown that some adult BMSC derive from the neural crest [8], [18]–[21].

The main objective of this study was consequently to specifically analyze the capacity of *in vivo* differentiation of the two distinct populations of BMSC: mesenchymal stem cells (MSC) and neural crest stem cells (NCSC), both isolated from adult bone marrow and recently characterized by Wislet-Gendebien et al. [21, 22 retracted in 55], when injected into lesioned brain. Indeed, we know that bone marrow NCSC are present in low proportion inside primary BMSC cultures compared to the MSC [21, 22 retracted in 55]. Consequently, a graft of pure bone marrow NCSC could lead to different results than observed with BMSC and could be able to restore brain lesions through a neural differentiation process in a larger extent, due to their neural crest developmental origin. We therefore grafted NCSC and MSC pure populations into the brain of mice characterized by dopaminergic nigrostriatal pathway lesions (mimicking the dopaminergic cell loss in advanced stages of Parkinson’s disease) induced by previous 1-methyl-4-phenyl-2,3,5-tetrahydropyridine hydrochloride (MPTP-HCl) injections. We then investigated neural differentiation events and downstream effects on the nigrostriatal pathway integrity, in order to evaluate potential of NCSC and MSC therapeutic abilities once inside the lesioned brain.

## Materials and Methods

### Animal Care

*Wnt1-Cre/R26R*-LacZ double transgenic mice were used to isolate NCSC and MSC clones from adult bone marrow stromal cells cultures [22 retracted in 55]. 12 to 16-week-old wild type C57BL/6J mice (The Jackson Laboratory, Bar Harbor, ME, USA) were used as recipient mice for graft experiments. Animals were bred at the University of Liège Central Animal facility and experiments were performed in accordance with the rules set by the local animal ethics committee (ethical permit 1038) as well as the Swiss Academy of Medical Sciences.

### MPTP Administration

Five days before the cell graft experiment, 1-methyl-4-phenyl-1,2,3,6-tetrahydropyridine hydrochloride (MPTP-HCl) (Sigma-Aldrich, St-Louis, MO, USA) is suspended in sterile PBS solution at a concentration of 5 mg/mL. Mice received four intraperitoneal injections of 20 mg/kg MPTP-HCl, at two hours interval (100 to 120 µL for 25 to 30 g-weight mice), as already described in [23], [24], triggering acute bilateral dopaminergic neurons cell death.

### Cell Culture Procedures and Clonal Selection

Bone marrow cells from adult (8–10 week-old) *Wnt1-Cre/R26R*-LacZ mice were obtained from femoral and tibial bones by aspiration and were resuspended in MesenCult Medium (MesenCult, Stem Cells Technologies, Grenoble, France). After 24 hours, non-adherent cells were removed. After reaching confluence, BMSC were resuspended using 0,05% trypsin-EDTA (Life Technologies, Carlsbald, CA, USA) and then sub-cultured (750,000 cells/25 cm^2^) at 37°C, in a 95% O_2_/5% CO_2_ atmosphere. For clonal selection, passage 5 BMSC were seeded in a 96 well plate (Thermo Fisher Scientific, Langenselbold, Germany) at a mean dilution of 0.7 cell/well, in MesenCult Medium. At confluence, cells were dissociated with 0,05% trypsin-EDTA and subcultured in the same conditions.

### Genomic Validation of Wnt1-CRE/R26R-LacZ Recombination

DNA was isolated from NCSC_mix_ and MSC_mix_ using the QIAamp DNA Mini Kit extraction protocol (Qiagen, Germantown, MD, USA). Briefly, cells were incubated at 56°C with proteinase K in a lysis buffer for 10 min, then genomic DNA was purified through several silica-membrane-based steps (see manufacturer’s instructions). DNA amount was then calculated via a NanoDrop spectrophotometer (Thermo Scientific). Afterwards, DNA sequences of interest were amplified by polymerase chain reaction by mixing 500 ng of genomic DNA with Taq Polymerase (Promega) and specific primers (PGK-Neo: For-ATGGATTGCACGCAGGTTCTCC; Rev-CAGAAGAACTCGTCAAGAAGGC and actin: For-ATCTTGATCTTCATGGTGCTAGG; Rev-TGTTACCAACTGGGACGACATGG) in a T3000 thermocycler (Biometra, Göttingen, Germany).

### Cell Preparation and Transplantation

Just before the transplantation, two cell solutions containing respectively 5 NCSC clones and 5 MSC clones in equal numbers were prepared. Whereas NCSC_mix_ were already traceable thanks to their β-galactosidase activity, we needed to label MSC_mix_ with Cell Tracker Green (CTG) (Life Technologies) to allow their traceability *in vivo*. Mice were anesthetized with 100 mg/kg of a solution containing equivalent volumes of xylazine (Rompun, Bayer, Belgium) and ketamine (Ketalar, Pfijzer, Belgium). They were then placed into a stereotaxic frame (Benchmark, MyNeuroLab.com) and received one injection of 5×10^4^ cells suspended in 2 µL PBS (Life Technologies) in the right striatum (0,5 mm anterior, 2 mm lateral and 3 mm ventral, with respect to bregma). The intracerebral injection was performed using a Hamilton’s 5 µl syringe, coupled with a 26-gauge needle. The needle was left in place for few minutes before being retracted, to avoid reflux along the injection track. After the surgery, mice were placed under a warm lamp until their complete awakening.

### Brain Processing

At different delays following cell transplantation, animals were anesthetized with pentobarbital and sacrificed by intracardiac perfusion of ice-cold PBS, followed by paraformaldehyde (PFA) 4% (in PBS 0,1 M), at. Skulls were dissected and brains were immediately removed, post-fixed for 2 hours at 4°C in the same fixative then immersed overnight in a solution of sucrose 20% (in PBS 0,1 M). They were frozen by slow immersion in isopentane cooled on dry-ice. Coronal 14 µm-sections were cut using a cryostat, mounted on positively charged slides, and stored in −20°C for further experiments (30 slides covering the entirety of the striatum and 10 slides covering the entirety of the midbrain).

### DNA Extraction from Striatal Slices and PCR Validation of Survival Rate Evaluation

DNA was extracted from 14 µm-striatal slices (after −20°C storage) using the PrepFiler Forensic DNA Extraction Kit (Life Technologies). Right striatum was microdissected on about 10 slices and then scraped into a 1,5 mL microcentrifuge tube. After lysis with proteinase K and heat/shake treatment, genomic DNA was bound to PrepFiler Magnetic Particles and then eluted in order to amplify sequences of interest by PCR (See *Genomic validation of Wnt1-CRE/R26R-LacZ recombination* section).

### Immunostainings

Briefly, 14-µm brains slices (or cells on coverslips) were incubated for 1 hour with 10% normal donkey serum in PBS 0,1 M (supplemented with 0,3% Triton X-100 for intracellular antigens). For specific immunofluorescent staining, anti-nestin (1∶300, NB100-1604; Novus Biologicals, Littleton, CO, USA), anti-βIII-tubulin (1∶1000, MMS-435P; Covance, Princeton, NJ, USA), anti-GFAP (1∶1000, Z0334; Dako, Glostrup, Denmark), anti-TH (1∶250, ab112; Abcam, Cambridge, UK), anti-Sca-1 (1∶100, ab25195; Abcam), anti-Fzd-4 (1∶100, MAB194; R&D System, Minneapolis, MN, USA) and anti-CD24 (1∶200, ab64064; Abcam) were diluted in PBS 0,1 M overnight at 4°C. After three PBS washes, brains sections were incubated with FITC or Rhodamine Red X-conjugated secondary antibodies (1∶500; Jackson Immunoresearch Laboratories, West Grove, PA, USA) for 1 hour at room temperature. Nuclei were then counterstained with Hoescht 33342 (Molecular Probes, Life Technologies) and finally mounted in Q Path Safemount (Labonord, Templemars, France).

The same steps and panel of primary antibodies were used for immunochemistry, but stainings were acquired using peroxydase-coupled secondary antibodies (1∶500, Dako) and diaminobenzidine revelation.

Image acquisition and analysis were performed using a Zeiss AxioImager Z1 epifluorescent microscope (Zeiss, Zaventem, Belgique) coupled with FluoView software (Olympus, Artselaar, Belgique), and Olympus AX-70 microscope (Olympus) coupled with AnalySIS software (Olympus). The digitized images were adjusted for brightness and contrast, color-coded, and merged, when appropriate, using the NIH program ImageJ (Wayne Rasband, National Institute of Mental Health, Bethesda, MD, USA).

### Other Stainings

#### X-gal staining

Brains sections were incubated for 2 hours in PBS supplemented with Tris (pH 7,4) 20 mM, MgCl_2_ 2 mM, 0.02% NP-40, 0.01% Na-deoxycholate, K_3_Fe(CN)_6_ 5 mM (Sigma-Aldrich), K_4_Fe(CN)_6_ 5 mM (Sigma-Aldrich) and 1-methyl-3-indolyl-beta-Dgalactopyranoside 1 mg/ml (Sigma-Aldrich) suspended in DMSO. The reaction was stopped by PBS washes.

#### Carazzi hematoxylin coloration

Dry brain sections were placed in denatured ethanol and slightly heated for approximately 4 minutes, then were washed three times in milliQ water, before an incubation of 10 minutes in Carazzi hematoxylin. After three washes in water, sections were finally mounted with Q Path Safemount (Labonord).

### Quantification of Cell Survival and Number of Neurons in the SN and VTA

Cell survival was quantified as followed: To evaluate the number of NCSC_mix_ in the brains at each time point post-transplantation, X-gal staining was performed on 4 slides containing striatal slices (4 slides covering the 30 slides : e.g. slide 1–10–20 - 25). Nuclei were counterstained with Hoescht, and after superposition of X-gal/Hoescht staining, X-gal positive nuclei were counted in each striatal sections on the slides. We then normalized the number of X-gal positive nuclei for all the 30 sections covering the entirety of striatum, and expressed this number in % regarding the 5×10^4^ cells that were initially injected. To evaluate the number of MSC_mix_ in the brains at each time point post-transplantation, co-localization of Cell Tracker Green and Hoescht was used and the same countings and normalizations were performed.

To evaluate the nigrostriatal pathway integrity, SNpc and VTA neurons were counted as previously described in the followed MPTP-administration protocol [23]. Our neuronal counts were expressed as mean number of neurons per representative mesencephalic plane. For each mouse, sections covering the entire rostrocaudal axis of the mesencephalon were analyzed. The mean number of neurons for each representative mesencephalic plane was obtained by averaging the number of neurons counted from both right and left SNpc or VTA areas, respectively.

### Statistical Analysis

Data were analyzed statistically using Statistica 10 program (StatSoft, Tulsa, OK, USA). Results are reported as mean ± standard error of mean, with the n described as the number of mice in each group. Level of statistical significance was set at p<0.05.

## Results

### Clonal Selection of NCSC and MSC from Adult Bone Marrow

**Figure 1:**
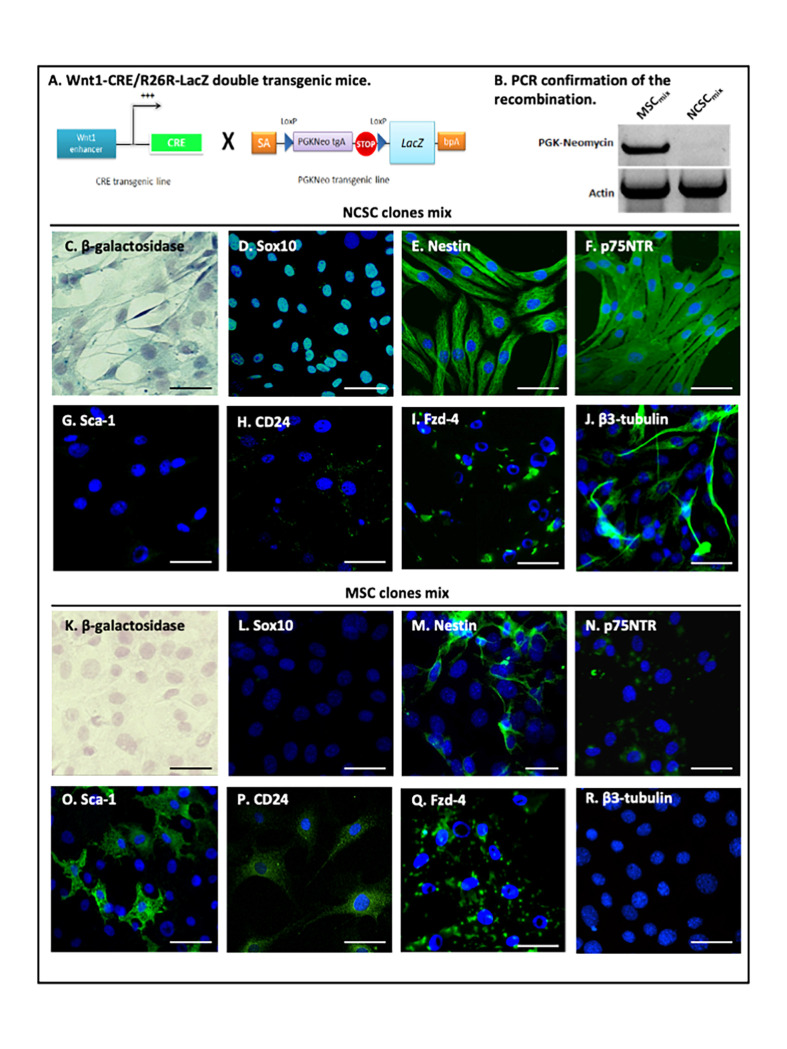
In vitro characterization of NCSC_mix_ and MSC_mix,_ isolated from adult Wnt1-Cre/R26R-LacZ mouse bone marrow. BMSC were harvested from double-transgenic Wnt1-CRE/R26R-LacZ mouse bone marrow (A). Amplification of the PGK-Neo cassette confirmed that NCSC_mix_ underwent recombination, conversely to MSC_mix_ (B). NCSC_mix_ were β-galactosidase positive (*blue*, C), whereas MSC_mix_ were not (*blue*, K) (Hematoxylin–stained nuclei). NCSC_mix_ expressed neural crest-associated proteins Sox10 (*green*, D), Nestin (*green*, E) and p75NTR (*green*, F). MSC_mix_ were Sox10-negative (*green*, L), weakly p75NTR-positive (*green*, N), and only a small proportion (<15%) of cells expressed nestin (*green*, M). MSC_mix_ also expressed Sca-1 (*green*, O) and CD24 (*green*, P) while NCSC_mix_ did not (*green*, G-H), and both types of cells were positive for Fzd-4 (*green*, I-Q). In NCSC_mix_, some β-tubulin-expressing cells were detected (in MesenCult medium) (*green*, J), but no cell in the MSC_mix_ was β-tubulin-positive in those conditions (*green*, R) (DAPI-stained nuclei). (Scale bars = 30 µm).

Since we previously demonstrated that neural crest stem cells were mainly composed of nestin-positive cells [22 retracted in 55] and that the number of nestin-positive cells increased with the number of passages [25 retracted in 56], we decided to perform clonal selection of NCSC and MSC starting from passage 5, which should theoretically give us equal chances to isolate NCSC or MSC. Single cell BMSC were placed in a 96-wells plate in MesenCult medium allowing 1.2% of cells to proliferate. NCSC clones or MSC clones were then pooled together creating two distinct and pure population: NCSC_mix_ and MSC_mix._ We first verified that cells in NCSC_mix_ were effectively derived from initial embryonic neural crest cells that underwent Cre-Lox recombination, conversely to MSC clones that were not neural crest-derived (Fig. 1 A–B). NCSC_mix_ (β-galactosidase-positive cells; Fig 1.C) and MSC_mix_ (β-galactosidase-negative cells; Fig 1.D) were then characterized *in vitro*. As previously described [22 retracted in 55], NCSC_mix_ were Sox10-positive (Fig 1.D), nestin-positive (Fig. 1.E) and p75^NTR^-positive (Fig. 1.F) while MSC_mix_ were Sox10-negative (Fig. 1.L), less than 15% were nestin-positive (Fig 1.M) and weakly p75^NTR^-positive (Fig. 1.N). MSC_mix_ also expressed Fzd-4 (Fig. 1.Q), Sca-1 (Fig. 1.O) and CD24 (Fig. 1.P) while NCSC_mix_ were only positives for Fzd-4 (Fig. 1.I). Additionally, βIII-tubulin positive cells were observed in NCSC_mix_ when cultivated in Mesencult medium (without any differentiation protocol, Fig. 1.J). At the opposite, no βIII-tubulin positive cells were observed among MSC_mix_ (Fig. 1.R). However, NCSC_mix_ and MSC_mix_ were both negative for more mature or specific neuronal markers like MAP2ab or TH and for GFAP (data not shown). We then decided to characterize NCSC_mix_ and MSC_mix_ differentiation and therapeutic abilities *in vivo* using the MPTP mouse model.

### MPTP Mouse Model Validation

**Figure 2:**
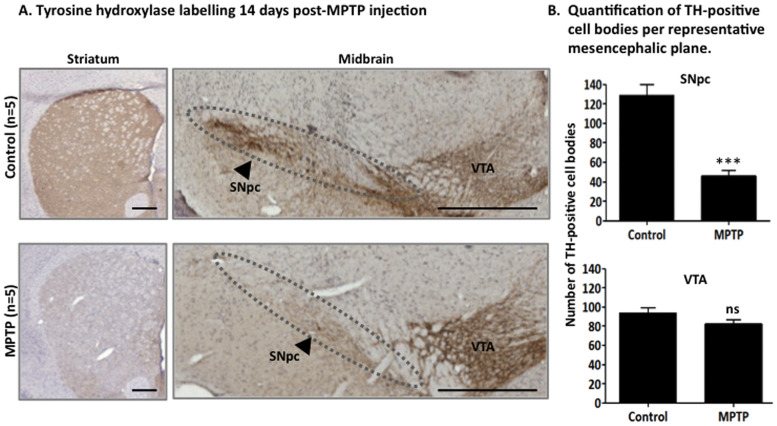
Validation of the MPTP mouse model of dopaminergic cell degeneration. **A.** Tyrosine hydroxylase (TH) immunostaining (*brown*, hematoxylin-stained nuclei) of dopaminergic fibers in the striatum and neurons in the midbrain of control and acute MPTP-treated mice. **B.** Number of TH-positive cell bodies per representative mesencephalic plane (Mean ± SEM). The number of dopaminergic neurons in the SNpc of MPTP-treated mice is significantly decreased compared to control (p<0,001, One-way ANOVA), while the VTA dopaminergic neurons are not strongly affected (p>0,05, One-way ANOVA). (Scale bars = 500 µm).

In order to verify MPTP-injection impact on nigrostriatal system (classically affected in Parkinson’s disease) and validate our experimental model, we quantified the number of dopaminergic neurons by immunostaining of tyrosine hydroxylase (TH) (limiting enzyme in dopamine synthesis). As observed on Fig. 2.A, a drastic decrease in TH-positive fibers in the entire striatum was observed in MPTP-treated animals compared to control (>95%). At the midbrain level, TH-positive cell bodies in the Substantia Nigra *pars compacta* (SNpc) of MPTP-treated mice were quantified (45,93±5,86 cells per representative mesencephalic plane, n = 5) and revealed a significant decrease (60%) of the number of cells compared to the control condition (129,40±10,50 TH-positive neurons per representative mesencephalic plane; n = 5; One-way ANOVA, *p*<0,001; Fig. 2.A–B). Ventral tegmental area (VTA) provides an internal control zone: VTA dopaminergic neurons were affected by MPTP in a lesser extent than SNpc neurons [26], and the difference between controls and MPTP-treated animals was not significant, attesting of MPTP specificity for SNpc neurons (One-way ANOVA, p>0,05). Those results were consistent with already published studies [23] (Fig. 2.A–B).

### NCSC_mix_ and MSC_mix_ Survival Rate

**Figure 3:**
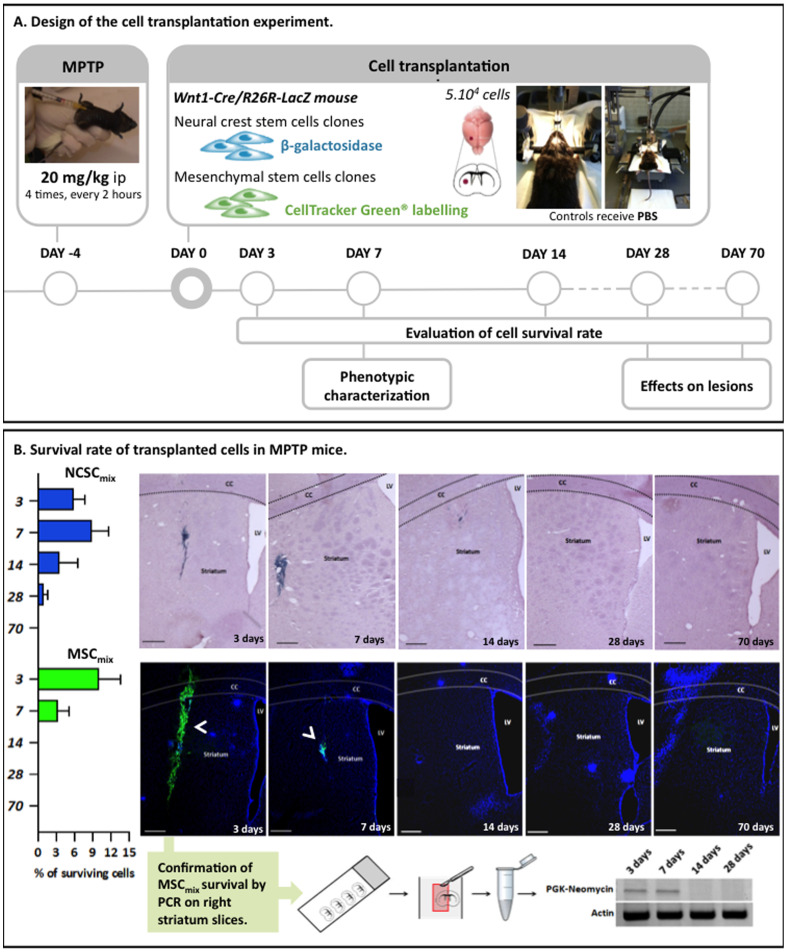
Intrastriatal transplantation of MSC_mix_/NCSC_mix_ in MPTP mice and survival rate of grafted cells at 3, 7, 14, 28 and 70 days after transplantation. **A.** Experimental design of the lesion and transplantation experiment. Adult C57Bl/6J male mice were injected with MPTP following the “classical” acute regimen. Five days after MPTP treatment MSC_mix_/NCSC_mix_ were injected into the right striatum of mice (MSC_mix_ were first stained with Cell Tracker Green in order to be detected *in vivo*). Survival rate evaluation and phenotypic characterization were performed at different delays post-graft. **B.** The number of surviving NCSC_mix_ in the right striatum (*blue* X-gal staining and *purple* Hematoxylin-stained nuclei) can reach 15% in the first week after transplantation, then the cells begin to disappear and after 4 weeks, we only observe a mean survival rate of 1%. Results are expressed in %, according to the 5×10^4^ injected cells (Mean ± SEM). MSC_mix_ (*green* CTG staining, *blue* DAPI-stained nuclei) seem to disappear more rapidly than NCSC_mix_, since no cells were observed starting from 14 days. (n≥3 for each group, at each delay post-transplantation). As CTG relevancy might be questioned, the temporary survival of cells was confirmed by PCR amplification of the PGK-Neomycin cassette in grafted MSC_mix_. (CC = Corpus callosum; LV = Lateral ventricle; Scale bars = 500 µm).

NCSC_mix_ and MSC_mix_ were injected in mice striatum 5 days post-MPTP injection. Surviving cells were quantified after 3, 7, 14, 28 and 70 days following cell graft, on all brain sections by counting nuclei co-localizing with X-gal staining or Cell Tracker Green (CTG) (for respectively grafted NCSC_mix_ and MSC_mix_). We first observed that transplanted cells (both NCSC_mix_ and MSC_mix_) staid tightly confined to the engraftment site, without any evident signs of migration through the brain tissue: no grafted cells were recovered into the lesioned SNpc or anywhere else inside the brain. As observed on Fig. 3.B, around 10% of NCSC_mix_ survived up to 7 days post-graft and 3% up to 14 days. After that delay, less than 1% of NCSC_mix_ were detected. Similar results were observed for MSC_mix_ as the mean survival rate of grafted cells was evaluated at 10% after 3 days. The survival rate at 7 days post-graft was decreased to 3%, and no grafted MSC were detected at 14 days post-graft. Control mice (injected with saline instead of MPTP) were also grafted with the same number of NCSC_mix_ or MSC_mix_ and we observed that the cells also disappeared within a 28 days timeframe (Fig. S1). As the stability of CTG fluorescence with time could be questioned, we confirmed by PCR that the cells were totally gone from the brain starting from 14 days after transplantation. In that purpose, we microdissected transplanted striatum slices and we showed that no PGK-Neo signal was observed starting from 14 days post transplantation (in saturating conditions) (Fig. 3.B).

### Phenotypic Characterization of Grafted MSC_mix_/NCSC_mix_

**Figure 4:**
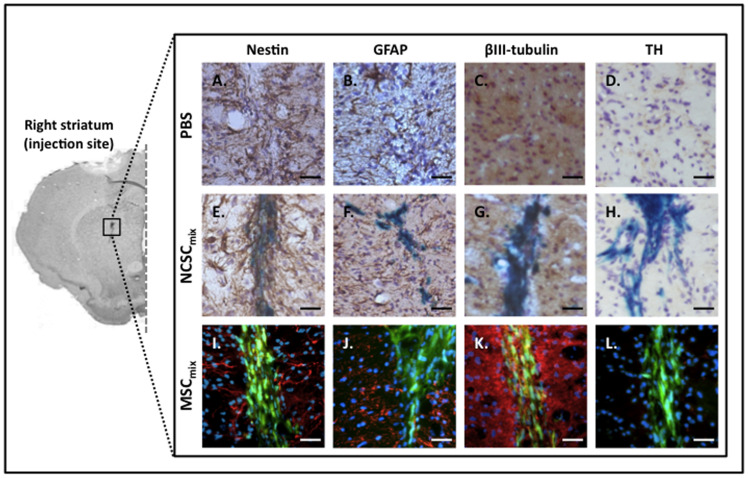
In vivo characterization of brain-injected NCSC_mix_ and MSC_mix_. All cells were implanted in the correct brain site, in each mouse that was included in the study. Transplanted NCSC_mix_ were detected by X-gal staining (*blue*) and MSC_mix_ were identified thanks to CTG staining (green). Grafted cells conserved their *in vitro* phenotype, whatever the delay post-transplantation : NCSC_mix_ were nestin-positive (*brown*, A) whereas MSC_mix_ were not (*red*, I). No cells did differentiate into GFAP-positive cells (NCSC_mix_, *brown*, F; MSC_mix_, *red*, J). Only few positive cells in NCSC_mix_ were βIII-tubulin-positive (*brown*, G), and no specific positivity was noticed for MSC_mix_ (*red*, K). Finally, grafted cells were negative for TH (NCSC_mix_, *brown*, F; MSC_mix_, *red*, J). Nestin and GFAP staining (*brown* or *red*) are detected around the transplanted cells and are linked with injection-induced inflammation (n≥3 for each group, at each delay post-transplantation). Scale bars = 25 µm.

At each time point post-transplantation, grafted NCSC_mix_ and MSC_mix_ were tested for nestin, glial fibrillary acidic protein (GFAP), βIII-tubulin, and tyrosine hydroxylase (TH) immunoreactivity. Similar results than the one observed *in vitro* were recovered as transplanted NCSC_mix_ expressed nestin (Fig. 4.E) while MSC_mix_ did not (Fig. 4.I) [22 retracted in 55], and both types of cells were GFAP-negative (Fig. 4.F,J). Regarding βIII-tubulin expression, specific co-localization was more difficult to appreciate since the environing brain tissue was entirely immunoreactive. However, only few positive cells were observed for NCSC_mix_ (Fig 4.G), and no evidence was noticed for MSC_mix_ (Fig. 4.K). Finally, no grafted cell (neither NCSC_mix_ nor MSC_mix_) differentiated into functional dopaminergic cells, as attested by their negativity for TH marker (Fig. 4.H,L and Fig. S2). According to those results, it appeared that the *in vivo* environment (MPTP-lesioned striatum) did not induce any modification in the phenotype of transplanted NCSC_mix_ and MSC_mix_.

### Effects of Cell Graft on MPTP-induced Lesions

**Figure 5:**
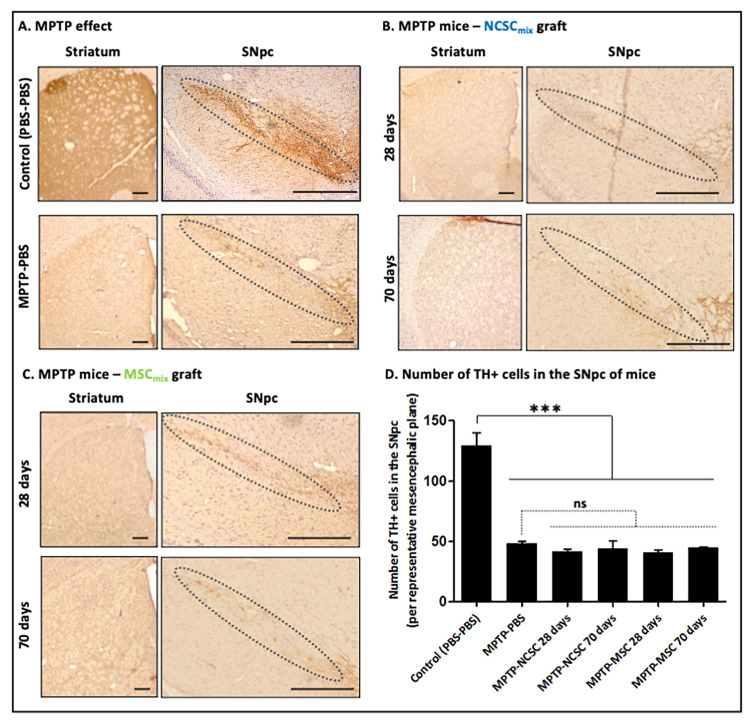
Evaluation of NCSC_mix_ and MSC_mix_ graft consequences on the number of host TH-positive neurons in MPTP-induced dopaminergic lesions. **A.** Effect of MPTP on the integrity of dopaminergic nigro-striatal pathway (at 28 days post-MPTP treatment). **B.** NCSC_mix_ graft in MPTP-treated mice. No increase in TH-positive (*brown*) striatal dopaminergic fibers and TH-positive cell bodies in the SNpc is observed, at 28 days as well as 70 days post- NCSC_mix_ transplantation. **C.** The same observations were carried out after MSC_mix_ transplantation. **D.** Number of TH-positive cell bodies in the SNpc of MPTP-treated mice that were transplanted with NCSC_mix_/MSC_mix_ (p>0,05; Kruskall-Wallis ANOVA). Scale bars = 250 µm.

We evaluated the integrity of nigro-striatal pathway thanks to the immunolabelling of tyrosine hydroxylase (TH). As already mentioned, the number of TH-positive neurons in the SNpc of MPTP-treated animals was decreased for more than 50%, compared to control animals, and the dopaminergic fibers in the striatum nearly completely disappeared (Fig. 5.A). After 28 and even 70 days following the cell transplantation, no improvement in striatal TH staining was observed in MPTP-injected mice in both conditions (NCSC_mix_-grafted group, Fig. 5.B and MSC_mix_-grafted group, Fig. 5. C). Similarly, we did not see any significant modification in the number of host TH-positive cell bodies into the SNpc of MPTP mice that received cell grafts (Fig. 5.D) (Kruskal-Wallis ANOVA, p>0,05).

## Discussion

Adult bone marrow stromal cells (BMSC) are of significant interest in cell therapy, regarding their accessible location, low immunogenicity and multipotentiality. Previous studies already described their neural differentiation abilities [27], [28], then confirming their potential use in the treatment of neurological diseases. Moreover, the presence of neural crest derived cells into the adult bone marrow stroma [21, 22 retracted in 55] raised new hopes to obtain functional neurons from autologous adult stem cells [8]. Recently, a clinical trial described unilateral transplantation of autologous whole BMSC population into the subventricular zone (SVZ) of PD patients, and reported reserved clinical improvement with no adverse effects, such as tumor formation [29], [30]. Whereas those results were based on clinical observations and Unified Parkinson’s disease Rate Scale scores, the mechanisms underlying the reported improvements are completely unknown.

To the light of those observations, the main objective of this study was to determine if bone marrow neural crest stem cells (NCSC) were responsible for the positive impact of bone marrow stromal cells in several PD models rather than mesenchymal stem cells (MSC). In this study, we addressed the aspect of neural differentiation abilities of pure NCSC or MSC populations by directly injecting those cells into the lesioned brain. In this purpose, the model we selected was an acute lesion of dopaminergic system, induced by 1-methyl-4-phenyl-1,2,3,6-tetrahydropyridine (MPTP) injection [23], [31], [32]. Consequently, cells were grafted five days after MPTP injection in order to pass up ongoing cell death and acute inflammatory events [33]–[35], with the aim of confirming if a potential enhancement was properly due to phenotypic plasticity and neural differentiation of injected cells. In those conditions, neither NCSC nor MSC survived for more than 28 days into the lesioned brain, neither underwent phenotypic modifications compared to their *in vitro* state before graft. Therefore, it wasn’t surprising not to observe any enhancement in nigro-striatal pathway integrity, suggesting that NCSC and MSC were not able to successfully differentiate into neural cells and to integrate and connect with host neurons in acute MPTP-treated mice.

Discrepancies of our observations with previous studies [36] could be justified regarding the chronic MPTP-injection protocol that leads to completely different degeneration kinetics [37]. Indeed, acute-MPTP mouse model do not reflect a real phenomenon of progressive degeneration, we might miss several events that could prompt grafted cells to adapt their phenotype. On the other hand, grafting stem cells in chronic-MPTP mice would be trickier to characterize, as both neuroprotective and neurorestorative events could simultaneously occur.

Nonetheless, our results reinforce the current controversy on BMSC neural differentiation ability. Indeed, whereas loads of papers describe *in vitro* specific neural proteins expression in BMSC after various neural induction protocols [38]–[41], few data provide convincing evidence for a neuron-specific electrophysiological signature of the differentiated cells, namely the elicitation of action potentials [42]. Moreover, the expression of neural specific proteins fails to characterize authentic functional and mature neurons, as some neural markers are already observed in primary cultures (without any differentiation protocols) [43], [44] and even after mesenchymal differentiation [45].

Concerning preclinical cell therapy experiments on PD animals models, neural differentiation-based therapy protocols were performed using stem cells from Wharton’s Jelly [46], dental pulp [47] and bone marrow [9], [48] that underwent various culture conditions before being transplanted in 6-hydroxydopamine (6-OHDA)-treated rats. Behavioral and pathological enhancements were observed in most of the studies, but the underlying mechanisms were not sufficiently detailed, and no evidence for an appropriate integration into the lesioned central nervous system (CNS) was observed. Conversely, significant improvements were observed in PD animal models that were transplanted with BMSC without any pre-differentiation step. In those conditions, no sign of neural differentiation was properly observed. Still, beneficial effects and rescue of dopaminergic neurons were noticed and mainly associated with neuroprotection [49], [50], trophic support (i.e. glial cell line-derived neurotrophic factor (GDNF) or epidermal growth factor (EGF) secretion) [50], [51] or anti-inflammation (attenuation of blood-brain barrier damages, microglia inactivation) [52]. Moreover, BMSC graft induced proliferation and migration of endogenous SVZ neuroblasts in two PD animal models [50], [53].

As regards the recent data about PD models and BMSC-based cell therapy, it appears that neural differentiation might not be responsible for physiopathological and clinical recoveries that are observed after BMSC transplantation in PD experimental models. Indeed, no evidence for *in vivo* functional neuronal replacement and CNS integration has been provided so far. Our results confirmed that bone marrow NCSC (in a pure population) are not any more competent than pure MSC nor whole BMSC to differentiate into neurons and integrate the damaged dopaminergic system. Altogether, it looks like adult BMSC are not a prime option for cell replacement therapies in the context of Parkinson’s disease. However, neuroprotective, neurotrophic and anti-inflammatory features characterizing BMSC are of greater interest as regards CNS lesions management, and still need to be fully characterized [54].

## Supporting Information

Figure S1 **Survival rate of grafted cells at 3, 7, 14, 28 and 70 days after transplantation of MSC_mix_/NCSC_mix_ in MPTP and control mice.** **A.** In MPTP mice, the number of surviving NCSC_mix_ in the right striatum (*blue* X-gal staining and *purple* Hematoxylin-stained nuclei) can reach 15% in the first week after transplantation, then the cells begin to disappear and after 4 weeks, we only observe a mean survival rate of 1%. In control mice, even if the number of surviving cells is higher at 3 and 7 days post-graft, the survival rate also decreases to 1% after 28 days. **B.** MSC_mix_ (*green* CTG staining, *blue* DAPI-stained nuclei) seem to disappear more rapidly than NCSC_mix_, since no cells were observed starting from 14 days, in both MPTP and control mice. **C.** Number of grafted cells that were recovered in mice brains at different delays post transplantation (Mean ± SEM) (CC = Corpus callosum; LV = Lateral ventricle; Scale bars = 500 µm).
(TIF)

Figure S2 **Tyrosine hydroxylase staining of brain-injected NCSC_mix_, at different delays post transplantation.** Transplanted NCSC_mix_ were detected by X-gal staining (*blue*). Grafted cells were negative for TH (*brown*) at 3, 7, 14 and 28 days after the cell injection (n≥3 for each group, at each delay post-transplantation). (Scale bars = 100 µm).
(TIF)
