# Expression of Concern: Adult Bone Marrow Neural Crest Stem Cells and Mesenchymal Stem Cells Are Not Able to Replace Lost Neurons in Acute MPTP-Lesioned Mice

**DOI:** 10.1371/journal.pone.0256488

**Published:** 2021-10-28

**Authors:** The PLOS ONE Editors

Following the publication of this article [1] concerns were raised regarding results presented in Figs 1, 2 and 5, and S1 Fig. Specifically,

- The SOX10 panel in Fig 1D appears similar to the SOX10 results in Figure 1E of [2], the SOX10 results in Figure 2C of [4], and the SOX10 results in Fig 1F of [5].
- The Nestin panel in Fig 1E appears similar to the Nestin results in Figure 1C of [2], and the Nestin results in Figure 2D of [3 retracted in 6].
- Within this article [1] the following panels appear more similar than would be expected from independent samples:
  - Figure 2A Control Midbrain (14 days post-MPTP injection) and Fig 5A Control (PBS-PBS) NSpc (28 days post-MPTP injection).
  - Figure 2A MPTP Striatum (14 days post-MPTP injection) and Fig 5B 70 days Striatum.
  - Fig 5A MPTP-PBS SNpc and Fig 5C 28 days SNpc.
  - S1B Fig MPTP Mice 14 days and S1B Fig Control Mice 70 days.

The authors explained that this study follows up from the results previously described in their *Cellular and Molecular Life Sciences* and *PLOS ONE* articles [2, 3 retracted in 6, 4], and preludes their *PLOS ONE* article [5], and clarify that the SOX10 panel in Fig 1D and the Nestin panel in Fig 1E represent the same experimental conditions as those panels presented in their other publications. As the original Figures presented in Fig 1D and 1E are not licensed for reproduction and distribution under the terms of the Creative Commons Attribution License (or Public Domain License for US gov), this article was republished on October 28, 2021 to remove this content and replace it with alternative relevant immunological characterization images. Please download this article again to view the correct version.

Furthermore, the authors explain that the wrong images were used to prepare the Fig 5A MPTP effect SNpc and MTPT + NCSC Stratum 70 days panels, the Fig 5C SNpc panel and the S1B Fig 70 days panel. The authors clarify that the wrong images were used inadvertently during figure preparation and explain that this error was introduced during the preparation of the figure; the correct samples were used for the quantification and the preparation of the associated graphs. The updated figures below were provided to relay the correct panels for Fig 5, but the authors explained that they were unable to provide a replacement panel for the S1B Fig 70 days result and S1 Fig has been updated to remove the incorrect panel. The originally published, uncorrected version of S1 Fig is provided in the S6 File below.

The updated Figs 1 and 5, and S1 Fig and their respective figure captions are provided here. The Board of Ethics and Scientific Integrity of the University of Liège investigated the overlap between the aforementioned panels and recommended the article be corrected. In addition, a member of *PLOS ONE*’s Editorial Board advised that the updated figures support the results and conclusions reported in the original article. However, the *PLOS ONE* Editors issue this Expression of Concern to due to the number of panels affected and the unavailability of the original MSC_mix_ transplanted brains Control Mice 70 days results.

**Fig 1:**
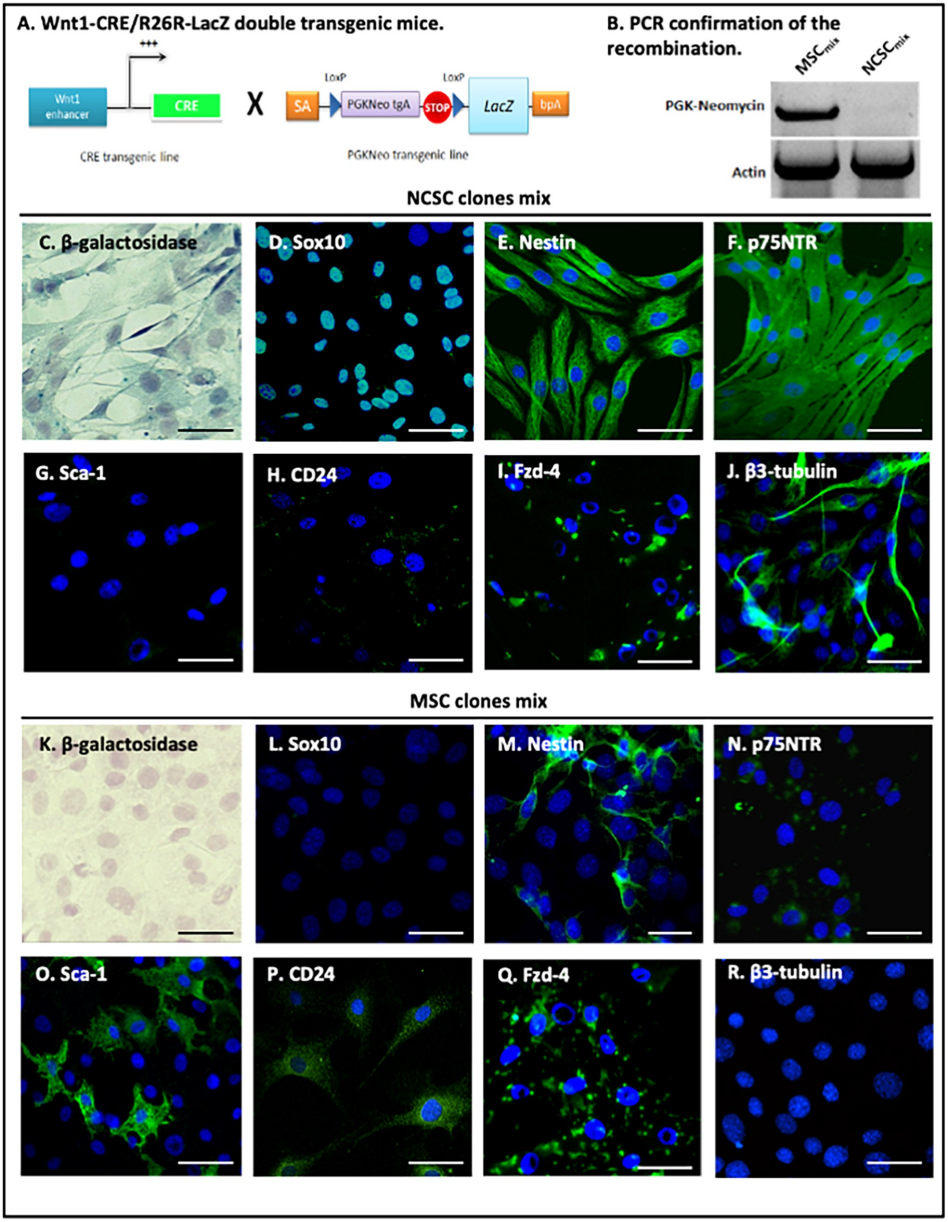
In vitro characterization of NCSCmix and MSCmix, isolated from adult Wnt1-Cre/R26R-LacZ mouse bone marrow. BMSC were harvested from double-transgenic Wnt1-CRE/R26R-LacZ mouse bone marrow (A). Amplification of the PGK-Neo cassette confirmed that NCSC_mix_ underwent recombination, conversely to MSC_mix_ (B). NCSC_mix_ were β-galactosidase positive (*blue*, C), whereas MSC_mix_ were not (*blue*, K) (Hematoxylin–stained nuclei). NCSC_mix_ expressed neural crest-associated proteins Sox10 (*green*, D), Nestin (*green*, E) and p75NTR (*green*, F). MSC_mix_ were Sox10-negative (*green*, L), weakly p75NTR-positive (*green*, N), and only a small proportion (<15%) of cells expressed nestin (*green*, M). MSC_mix_ also expressed Sca-1 (*green*, O) and CD24 (*green*, P) while NCSC_mix_ did not (*green*, G-H), and both types of cells were positive for Fzd-4 (*green*, I-Q). In NCSC_mix_, some β-tubulin-expressing cells were detected (in MesenCult medium) (*green*, J), but no cell in the MSC_mix_ was β-tubulin-positive in those conditions (*green*, R) (DAPI-stained nuclei). (Scale bars = 30 μm).

**Fig 5:**
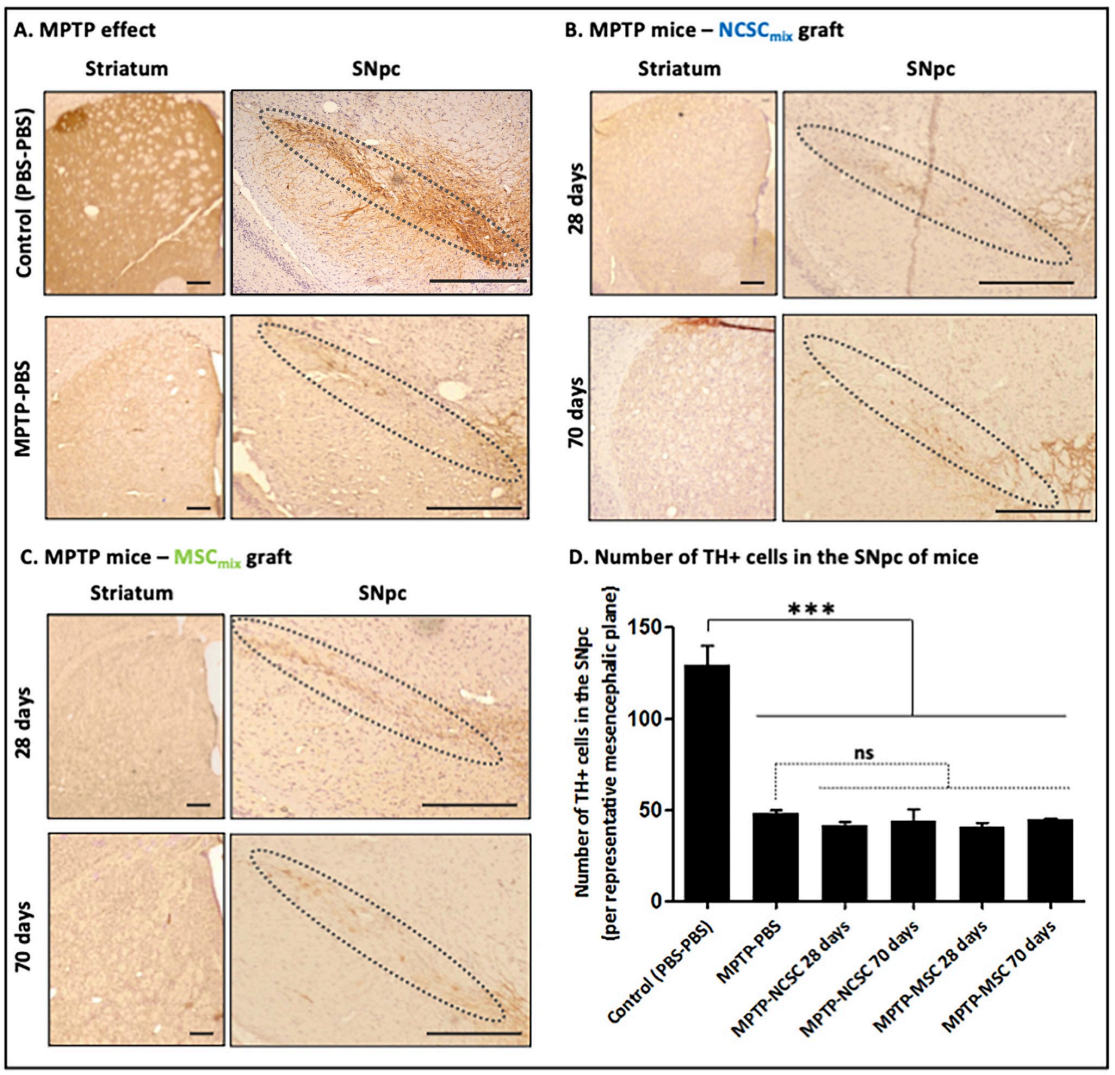
Evaluation of NCSCmix and MSCmix graft consequences on the number of host TH-positive neurons in MPTP-induced dopaminergic lesions. A. Effect of MPTP on the integrity of dopaminergic nigro-striatal pathway (at 28 days post-MPTP treatment). B. NCSC_mix_ graft in MPTP-treated mice. No increase in TH-positive (*brown*) striatal dopaminergic fibers and TH-positive cell bodies in the SNpc is observed, at 28 days as well as 70 days post- NCSC_mix_ transplantation. C. The same observations were carried out after MSC_mix_ transplantation. D. Number of TH-positive cell bodies in the SNpc of MPTP-treated mice that were transplanted with NCSC_mix_/MSC_mix_ (p>0,05; Kruskall-Wallis ANOVA). Scale bars = 250 μm.

## Supporting information

S1 Fig Survival rate of grafted cells at 3, 7, 14, 28 and 70 days after transplantation of MSCmix/NCSCmix in MPTP and control mice. A. In MPTP mice, the number of surviving NCSC_mix_ in the right striatum (*blue* X-gal staining and *purple* Hematoxylin-stained nuclei) can reach 15% in the first week after transplantation, then the cells begin to disappear and after 4 weeks, we only observe a mean survival rate of 1%. In control mice, even if the number of surviving cells is higher at 3 and 7 days post-graft, the survival rate also decreases to 1% after 28 days. B. MSC_mix_ (*green* CTG staining, *blue* DAPI-stained nuclei) seem to disappear more rapidly than NCSC_mix_, since no cells were observed starting from 14 days, in both MPTP and control mice. C. Number of grafted cells that were recovered in mice brains at different delays post transplantation (Mean ± SEM) (CC = Corpus callosum; LV = Lateral ventricle; Scale bars = 500 μm).
(JPG)

S1 File Data underlying updated Fig 1. (ZIP)

S2 File Raw data Figure 2. (ZIP)

S3 File Data underlying updated Fig 5. (ZIP)

S4 File Raw data S1 Fig. (ZIP)

S5 File Originally published, uncorrected article with copyrighted images removed. (PDF)

S6 File Originally published, uncorrected version of S1 Fig. (TIF)
