# Supplementary figures and images for: Adult Bone Marrow Neural Crest Stem Cells and Mesenchymal Stem Cells Are Not Able to Replace Lost Neurons in Acute MPTP-Lesioned Mice
Source: PLoS One. 2013 May 31;8(5):e64723. doi: 10.1371/journal.pone.0064723 (PMC3669410; doi:10.1371/journal.pone.0064723)

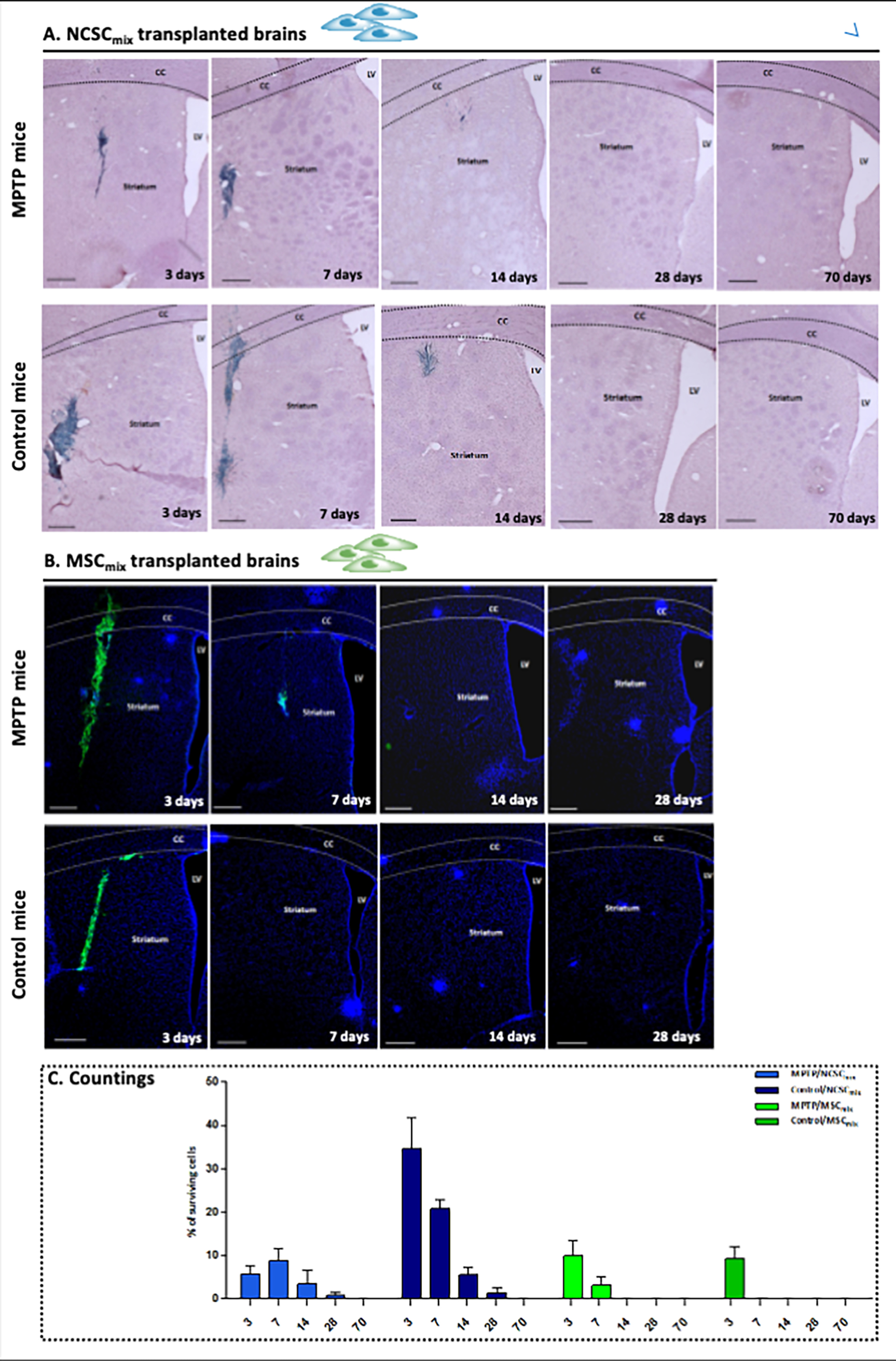

Supplement: Figure S1 — Survival rate of grafted cells at 3, 7, 14, 28 and 70 days after transplantation of MSCmix/NCSCmix in MPTP and control mice. A. In MPTP mice, the number of surviving NCSCmix in the right striatum (blue X-gal staining and purple Hematoxylin-stained nuclei) can reach 15% in the first week after transplantation, then the cells begin to disappear and after 4 weeks, we only observe a mean survival rate of 1%. In control mice, even if the number of surviving cells is higher at 3 and 7 days post-graft, the survival rate also decreases to 1% after 28 days. B. MSCmix (green CTG staining, blue DAPI-stained nuclei) seem to disappear more rapidly than NCSCmix, since no cells were observed starting from 14 days, in both MPTP and control mice. C. Number of grafted cells that were recovered in mice brains at different delays post transplantation (Mean ± SEM) (CC = Corpus callosum; LV = Lateral ventricle; Scale bars = 500 µm). (TIF) [file pone.0064723.s001.tif]

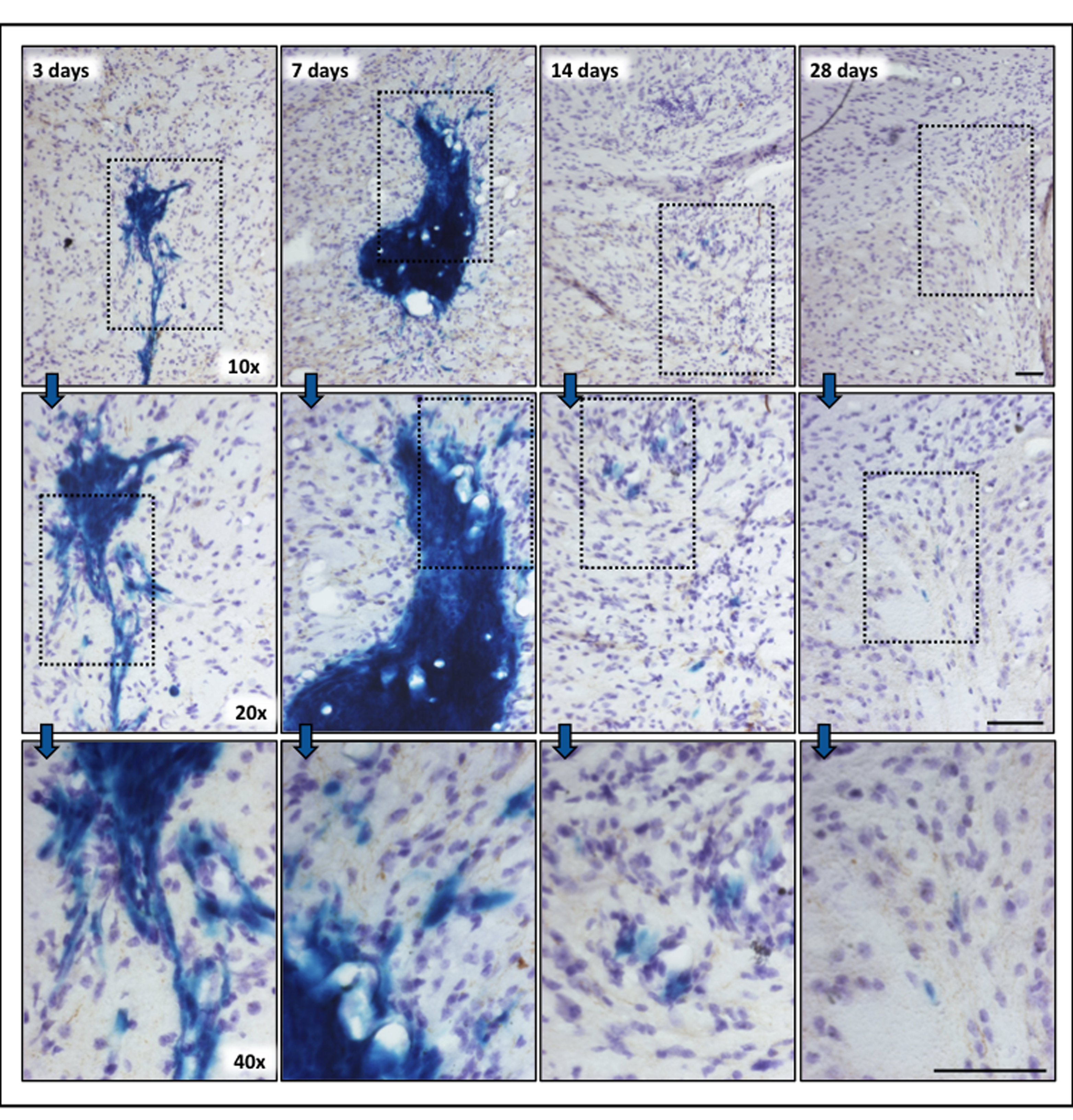

Supplement: Figure S2 — Tyrosine hydroxylase staining of brain-injected NCSCmix, at different delays post transplantation. Transplanted NCSCmix were detected by X-gal staining (blue). Grafted cells were negative for TH (brown) at 3, 7, 14 and 28 days after the cell injection (n≥3 for each group, at each delay post-transplantation). (Scale bars = 100 µm). (TIF) [file pone.0064723.s002.tif]
