# Supplementary figures and images for: Expression of Concern: Adult Bone Marrow Neural Crest Stem Cells and Mesenchymal Stem Cells Are Not Able to Replace Lost Neurons in Acute MPTP-Lesioned Mice
Source: PLoS One. 2021 Oct 28;16(10):e0256488. doi: 10.1371/journal.pone.0256488 (PMC8553060; doi:10.1371/journal.pone.0256488)

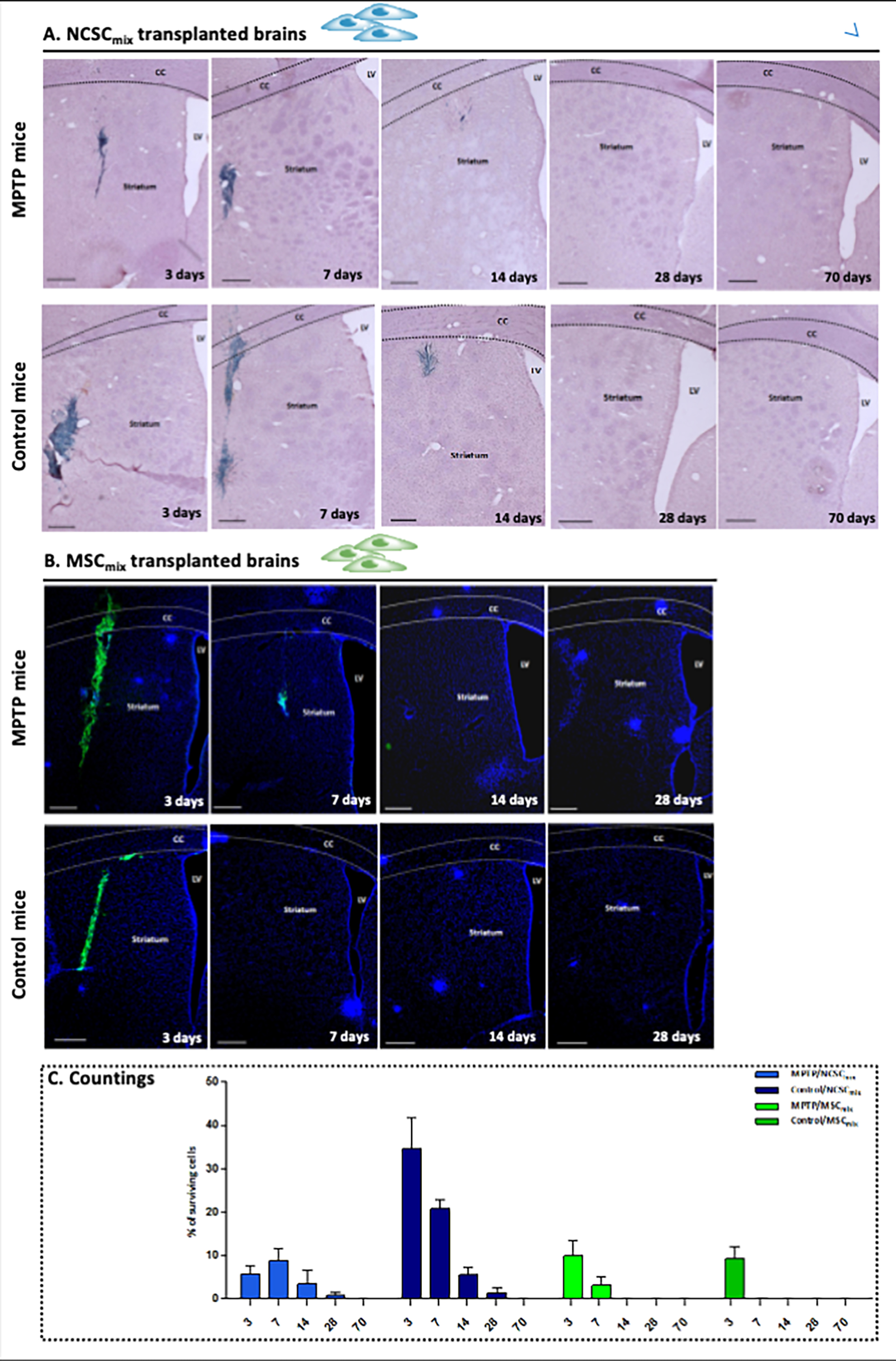

Supplement: S1 Fig — A. In MPTP mice, the number of surviving NCSCmix in the right striatum (blue X-gal staining and purple Hematoxylin-stained nuclei) can reach 15% in the first week after transplantation, then the cells begin to disappear and after 4 weeks, we only observe a mean survival rate of 1%. In control mice, even if the number of surviving cells is higher at 3 and 7 days post-graft, the survival rate also decreases to 1% after 28 days. B. MSCmix (green CTG staining, blue DAPI-stained nuclei) seem to disappear more rapidly than NCSCmix, since no cells were observed starting from 14 days, in both MPTP and control mice. C. Number of grafted cells that were recovered in mice brains at different delays post transplantation (Mean ± SEM) (CC = Corpus callosum; LV = Lateral ventricle; Scale bars = 500 μm). (JPG) [file pone.0256488.s001.jpg]

## Slide 1
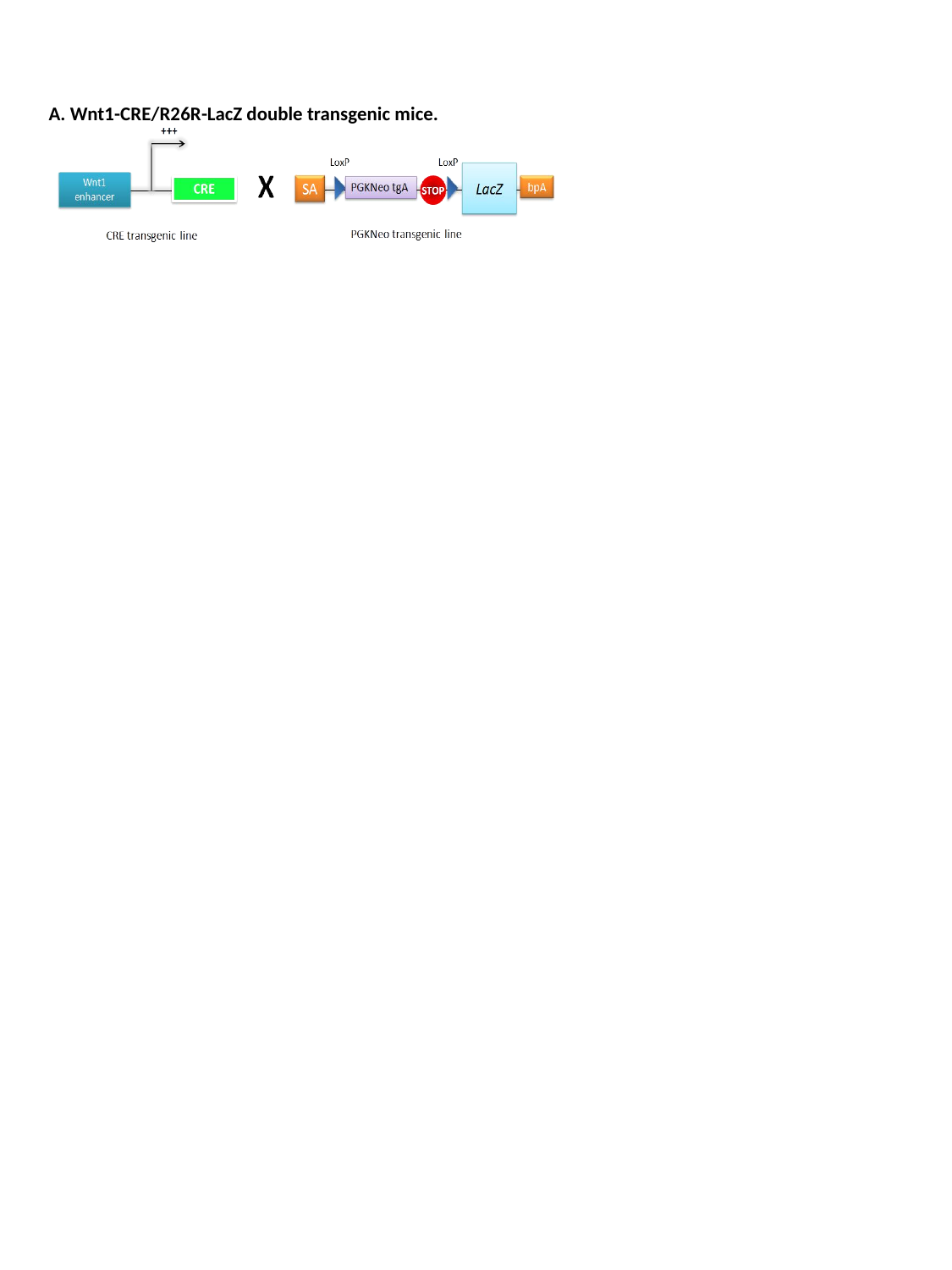

A. Wnt1-CRE/R26R-LacZ double transgenic mice.

Supplement: S1 File — (ZIP) [file pone.0256488.s002.zip › S1 File/Figure 1A S Wislet.pptx]

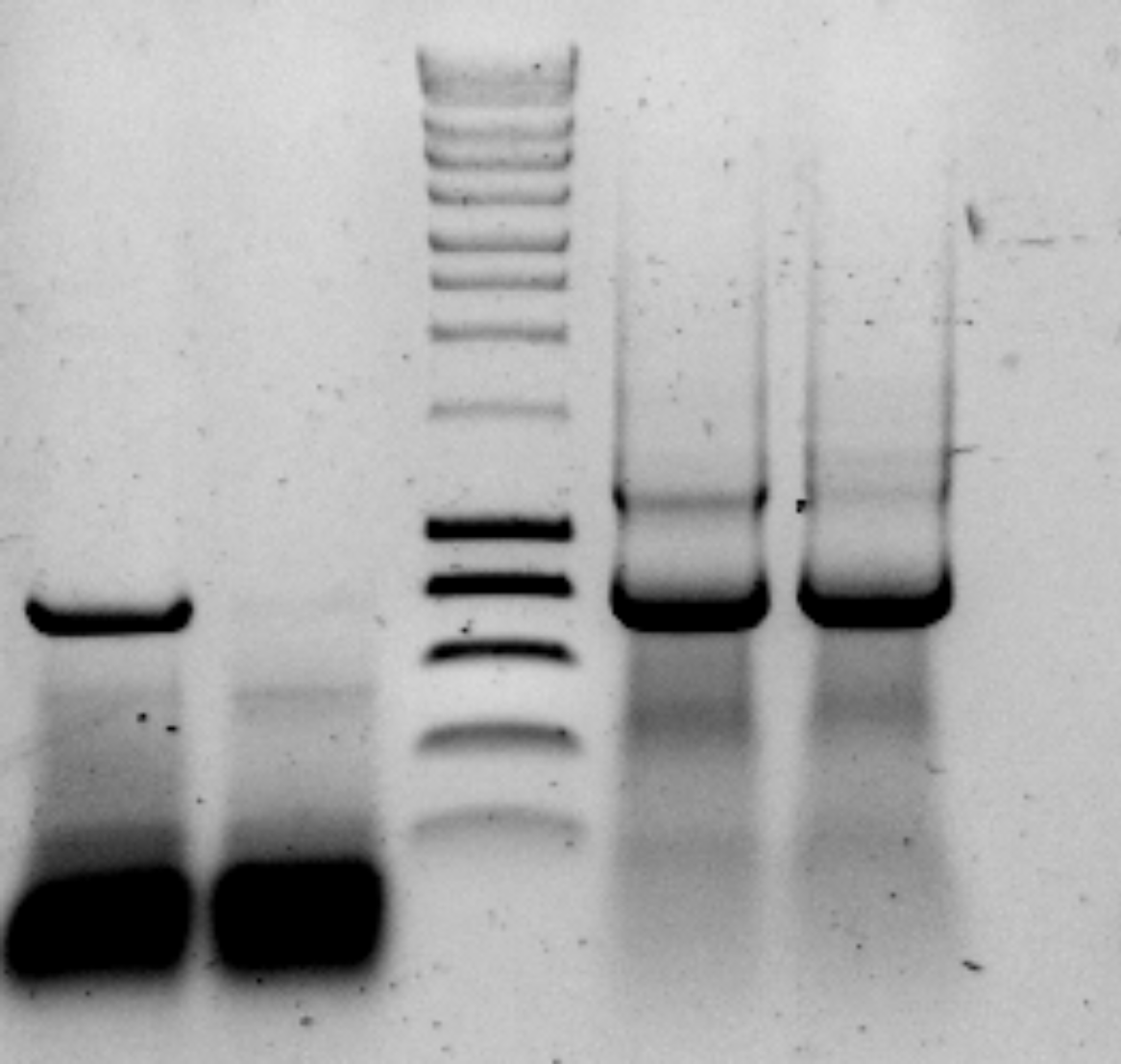

Supplement: S1 File — (ZIP) [file pone.0256488.s002.zip › S1 File/Figure 1B.tif]

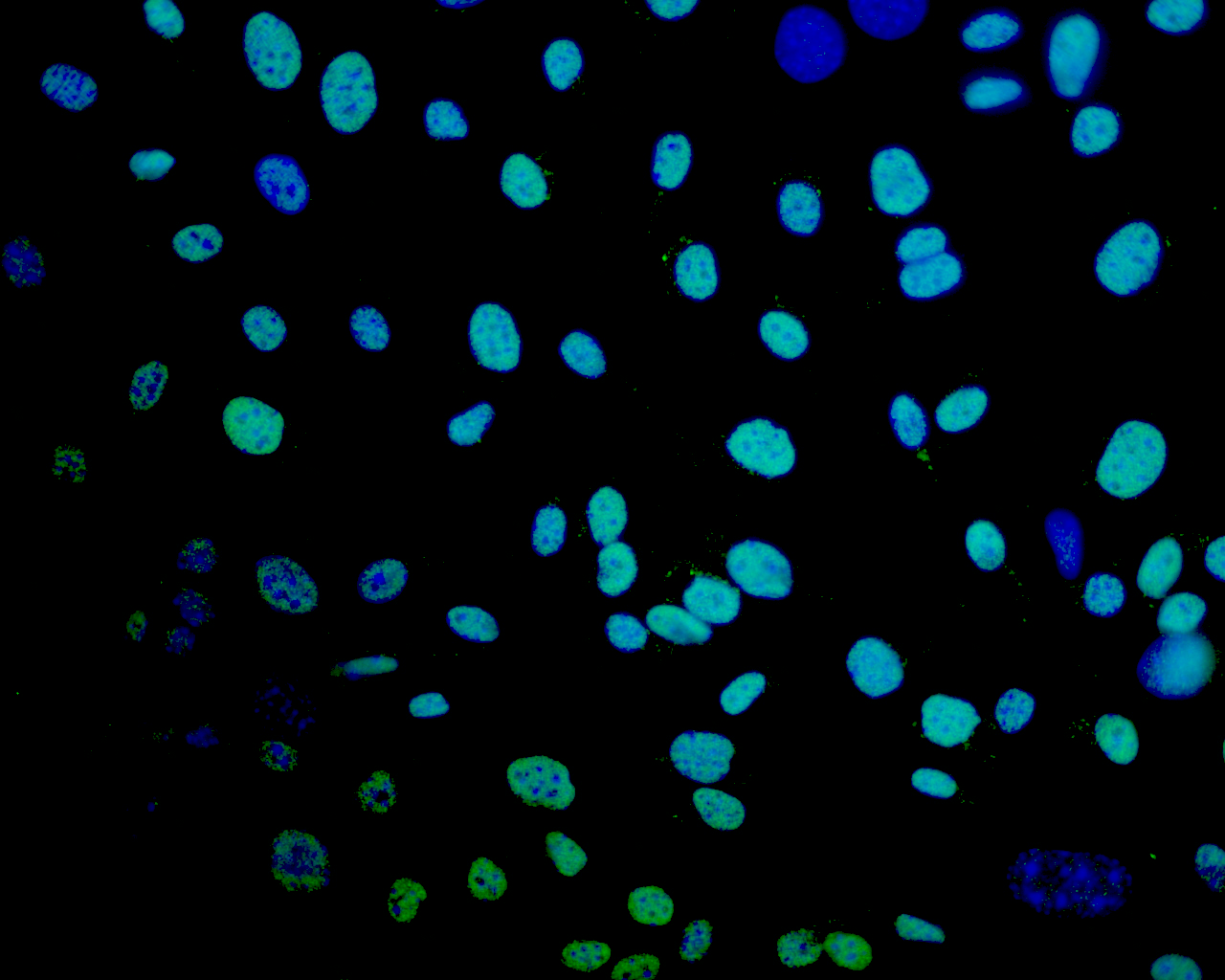

Supplement: S1 File — (ZIP) [file pone.0256488.s002.zip › S1 File/Figure 1D.tif]

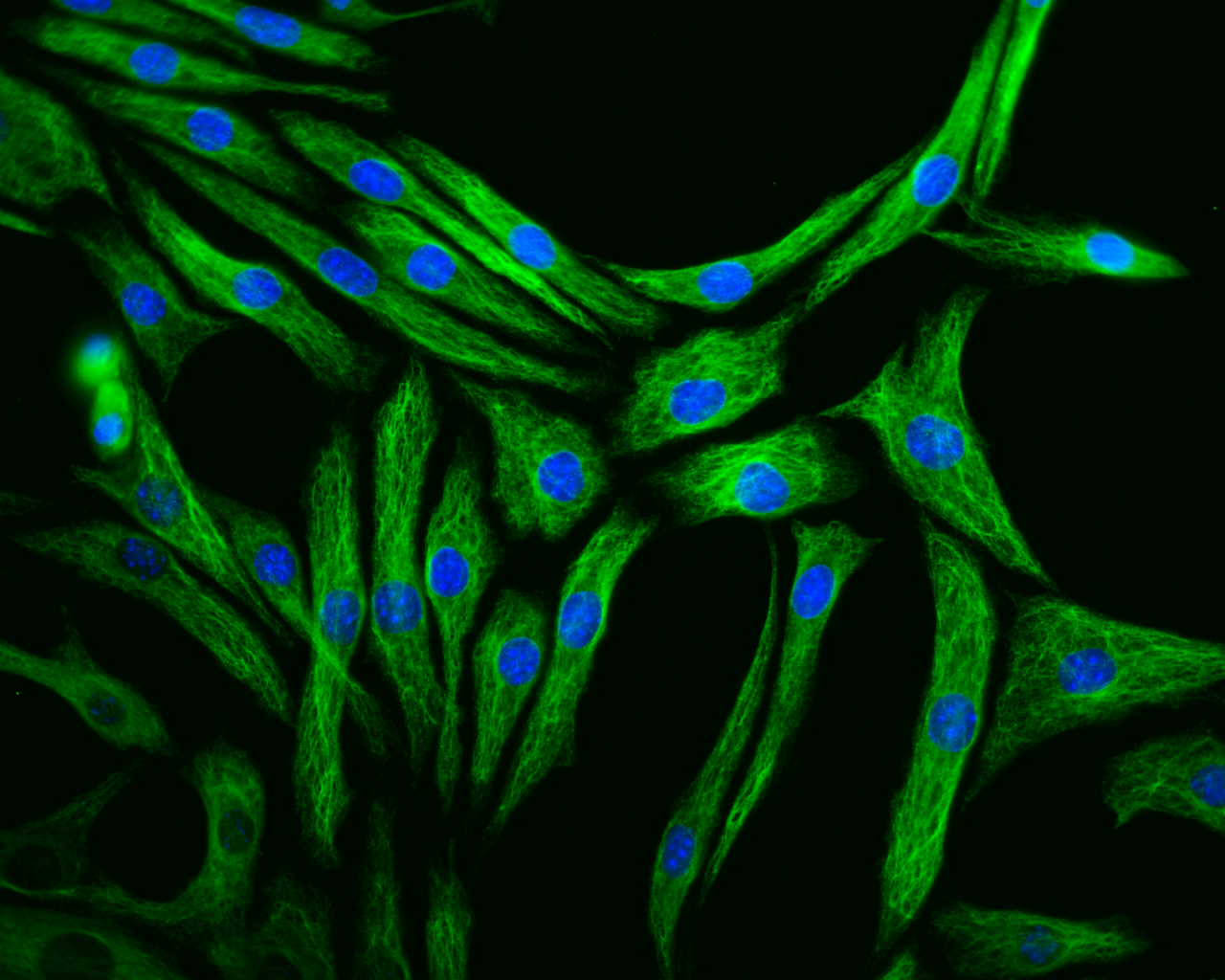

Supplement: S1 File — (ZIP) [file pone.0256488.s002.zip › S1 File/Figure 1E.tif]

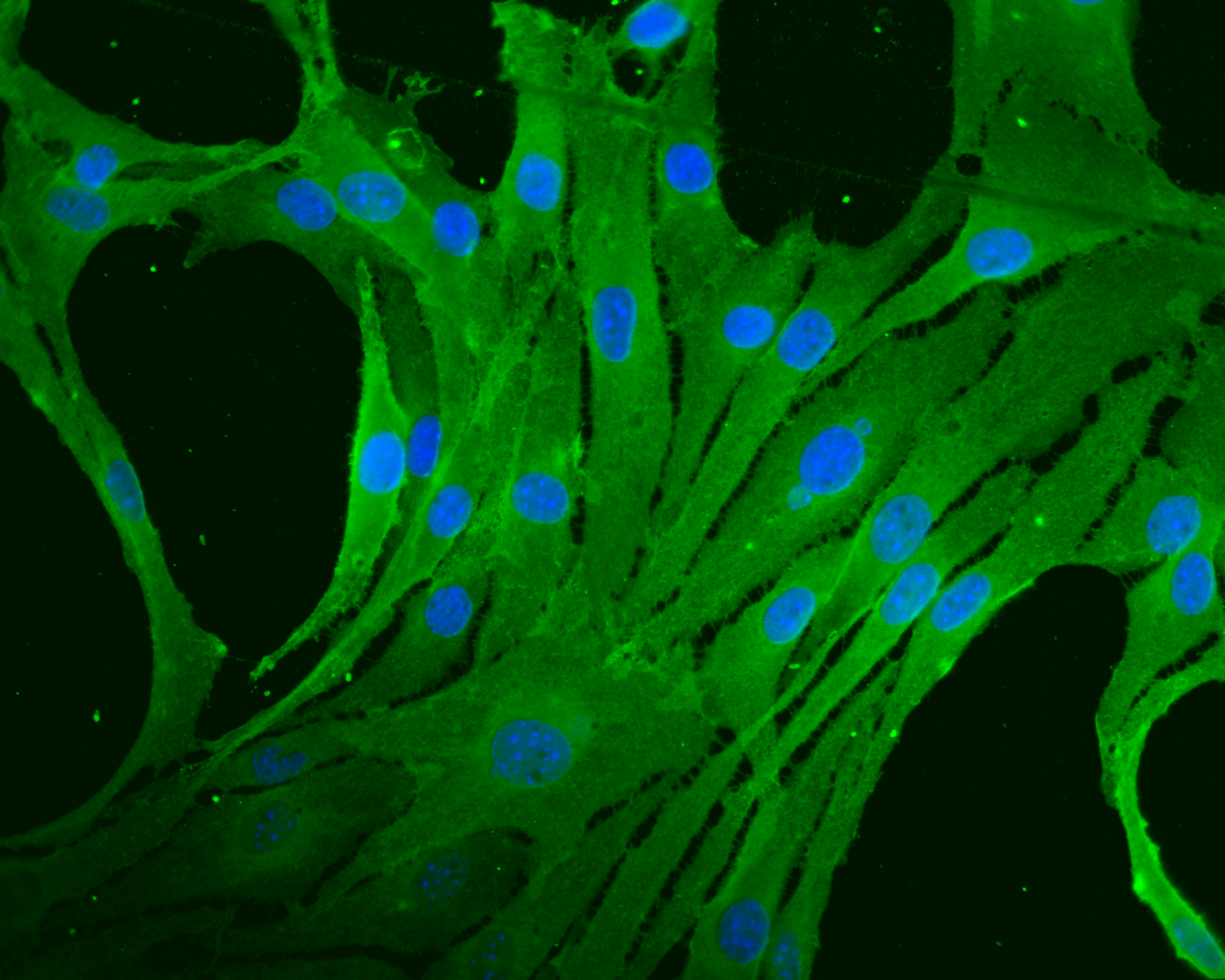

Supplement: S1 File — (ZIP) [file pone.0256488.s002.zip › S1 File/Figure 1F.tif]

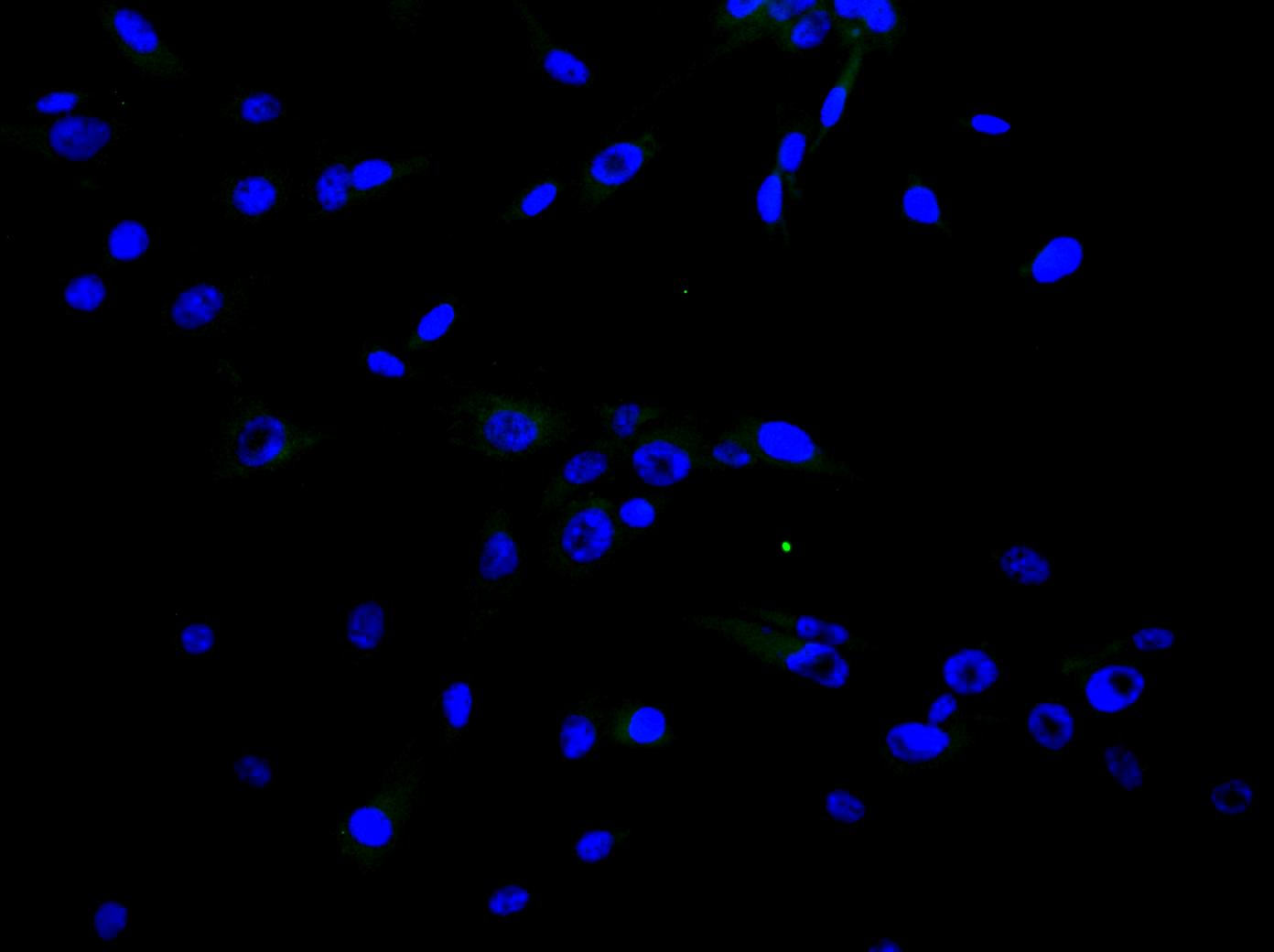

Supplement: S1 File — (ZIP) [file pone.0256488.s002.zip › S1 File/Figure 1G.jpg]

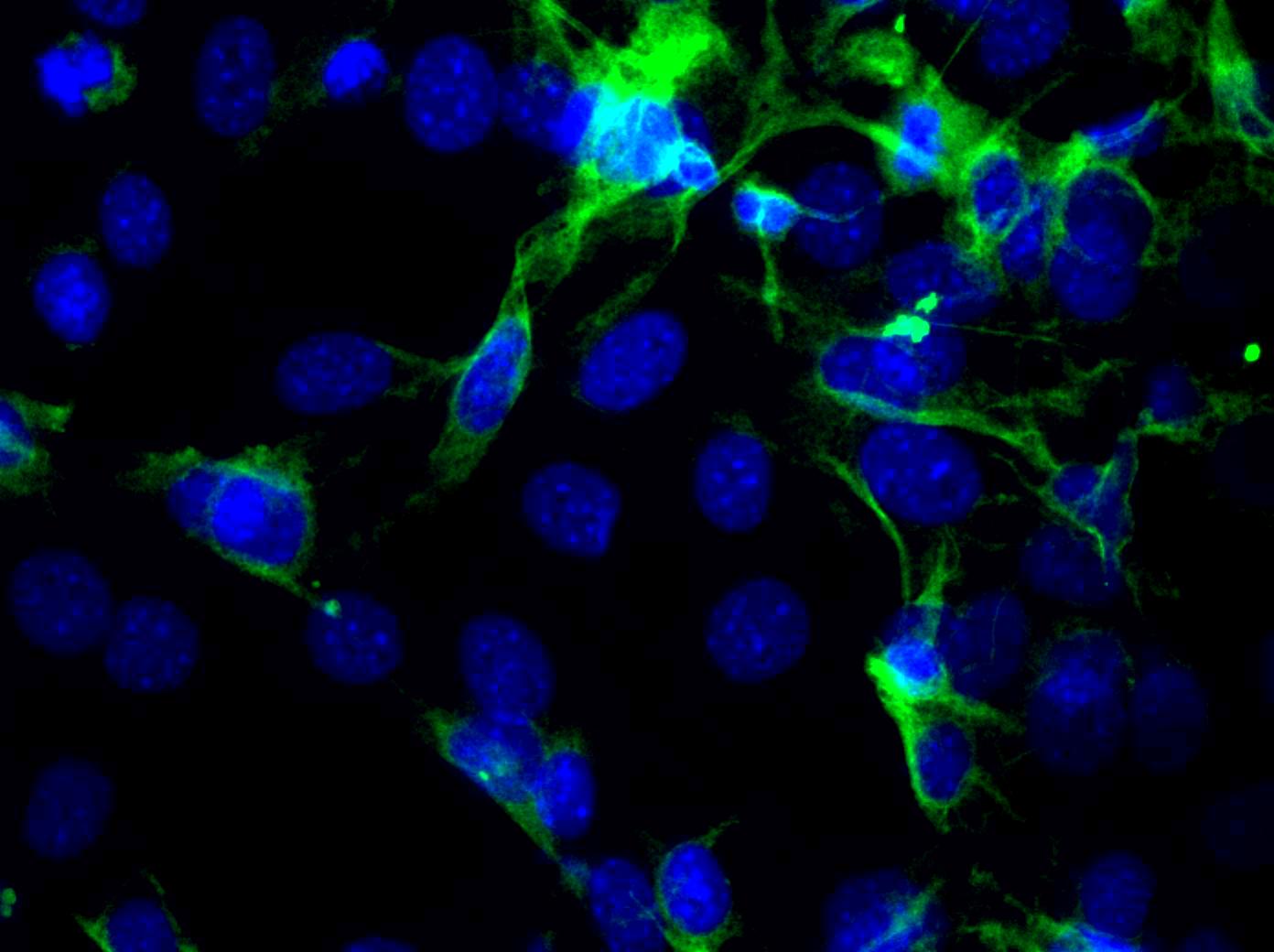

Supplement: S1 File — (ZIP) [file pone.0256488.s002.zip › S1 File/Figure 1M.jpg]

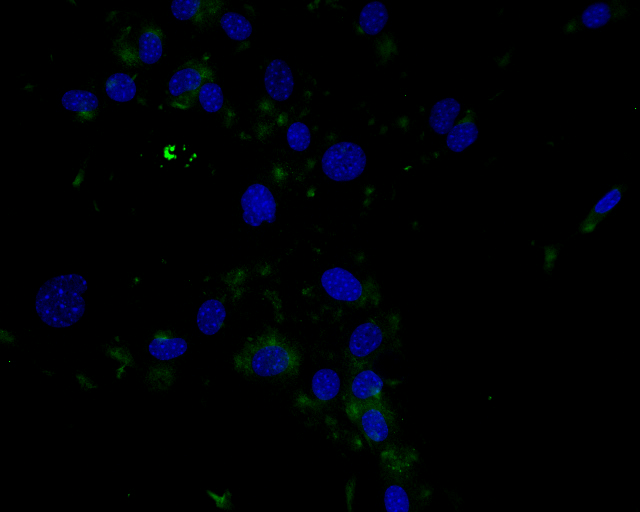

Supplement: S1 File — (ZIP) [file pone.0256488.s002.zip › S1 File/Figure 1N.tif]

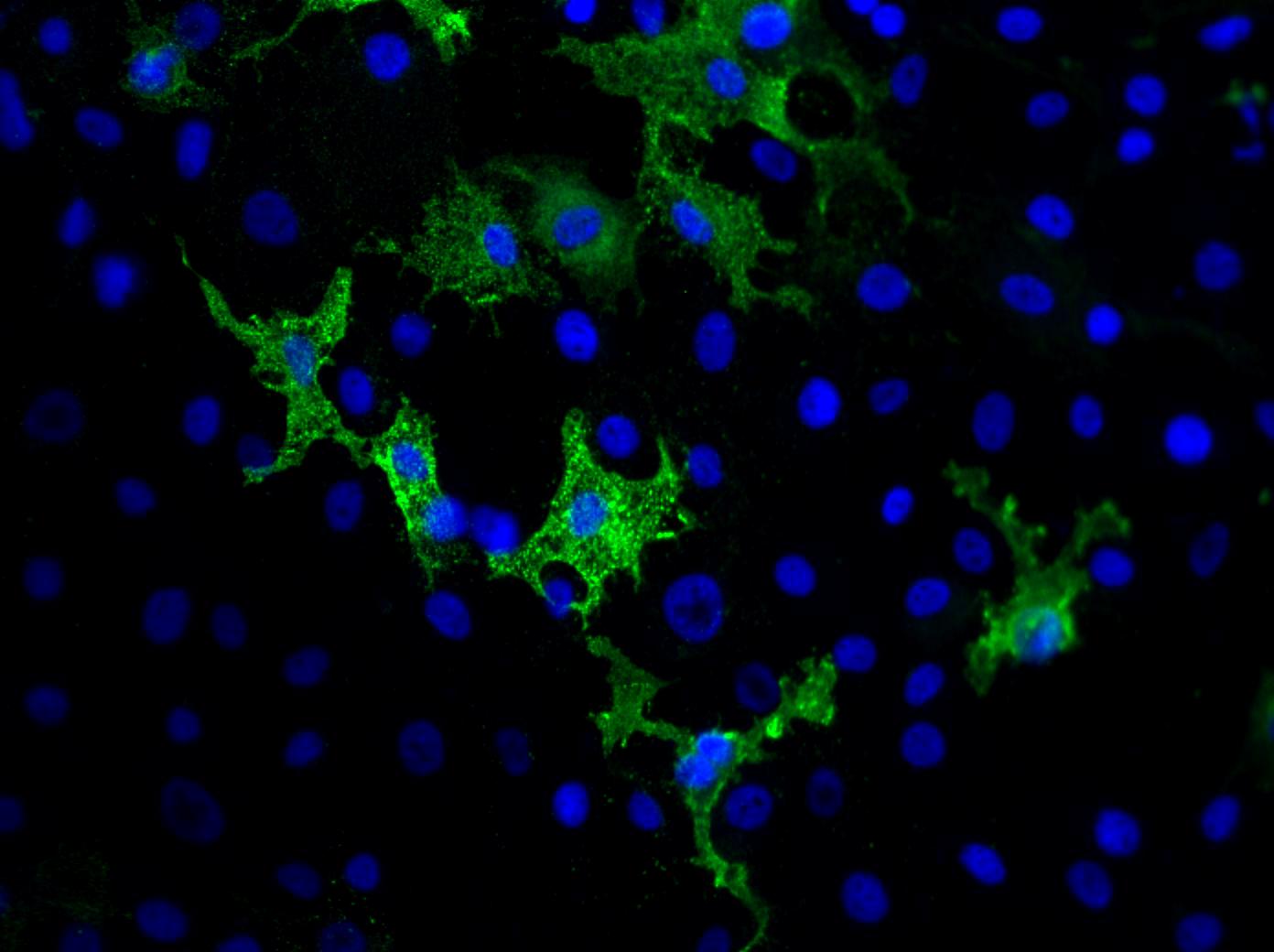

Supplement: S1 File — (ZIP) [file pone.0256488.s002.zip › S1 File/Figure 1O.jpg]

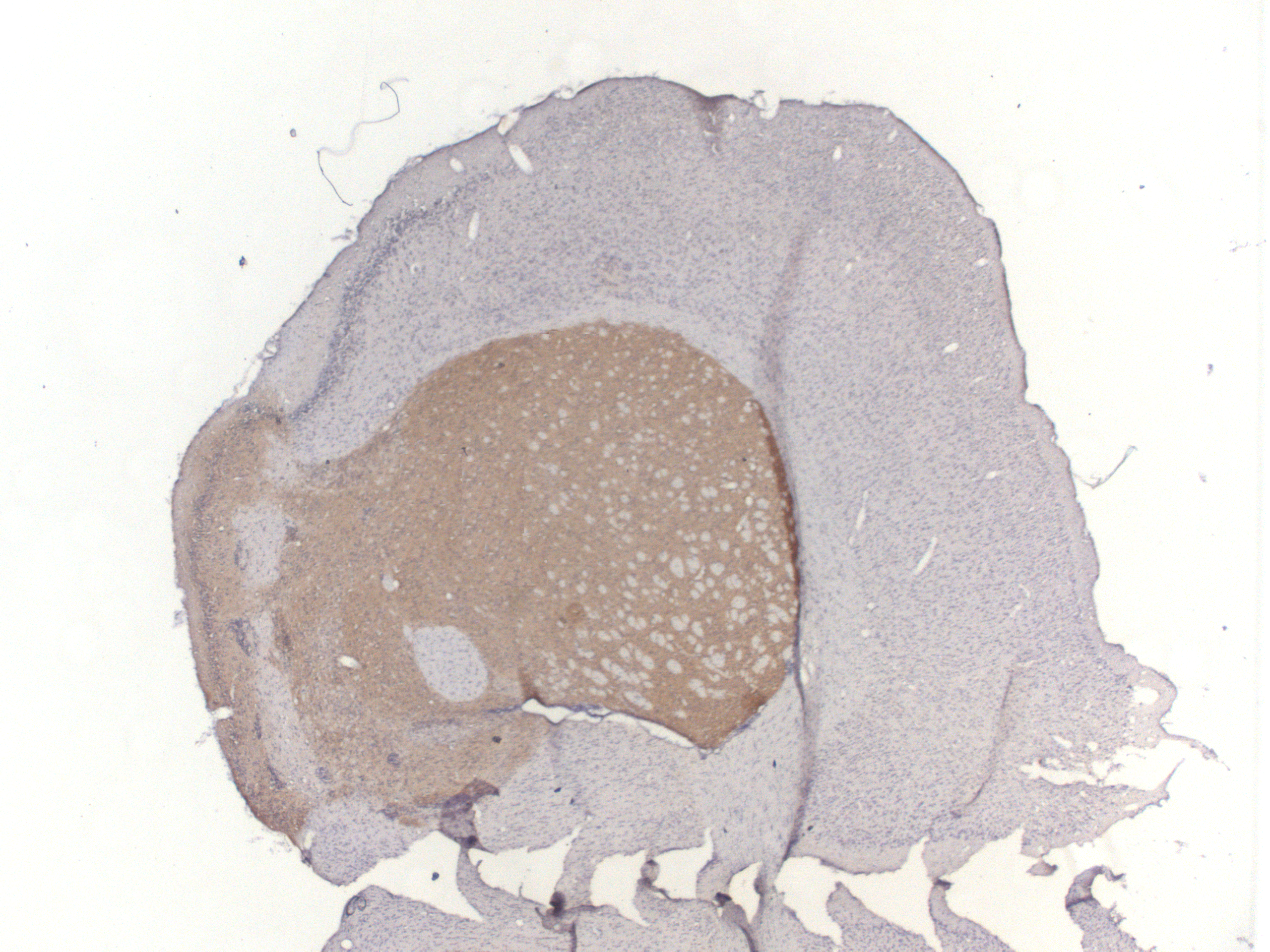

Supplement: S2 File — (ZIP) [file pone.0256488.s003.zip › S2 File/Figure 2A/Figure 2 CTRL Striatum.tiff]

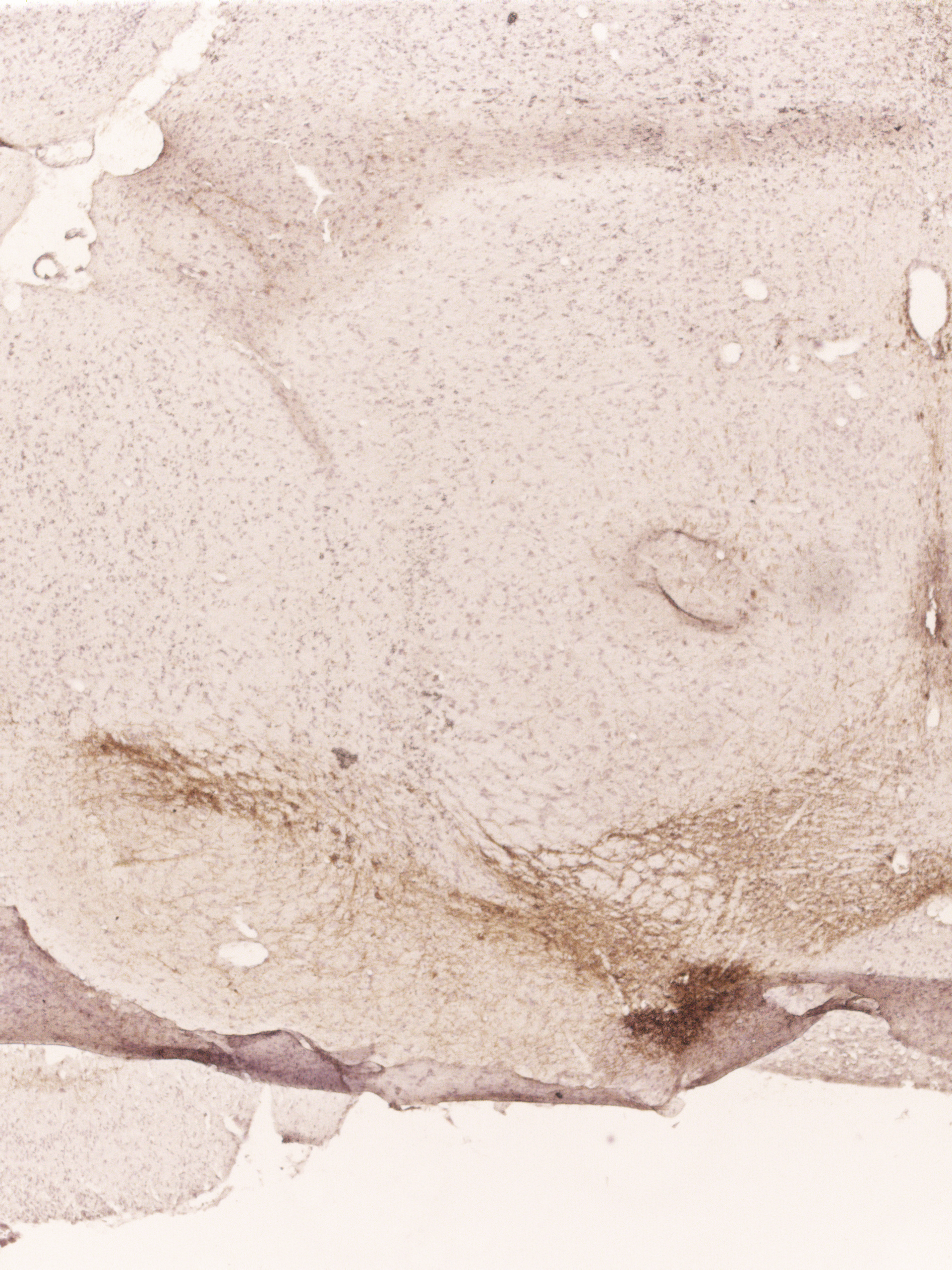

Supplement: S2 File — (ZIP) [file pone.0256488.s003.zip › S2 File/Figure 2A/Figure 2 Midbrain CTRL.tiff]

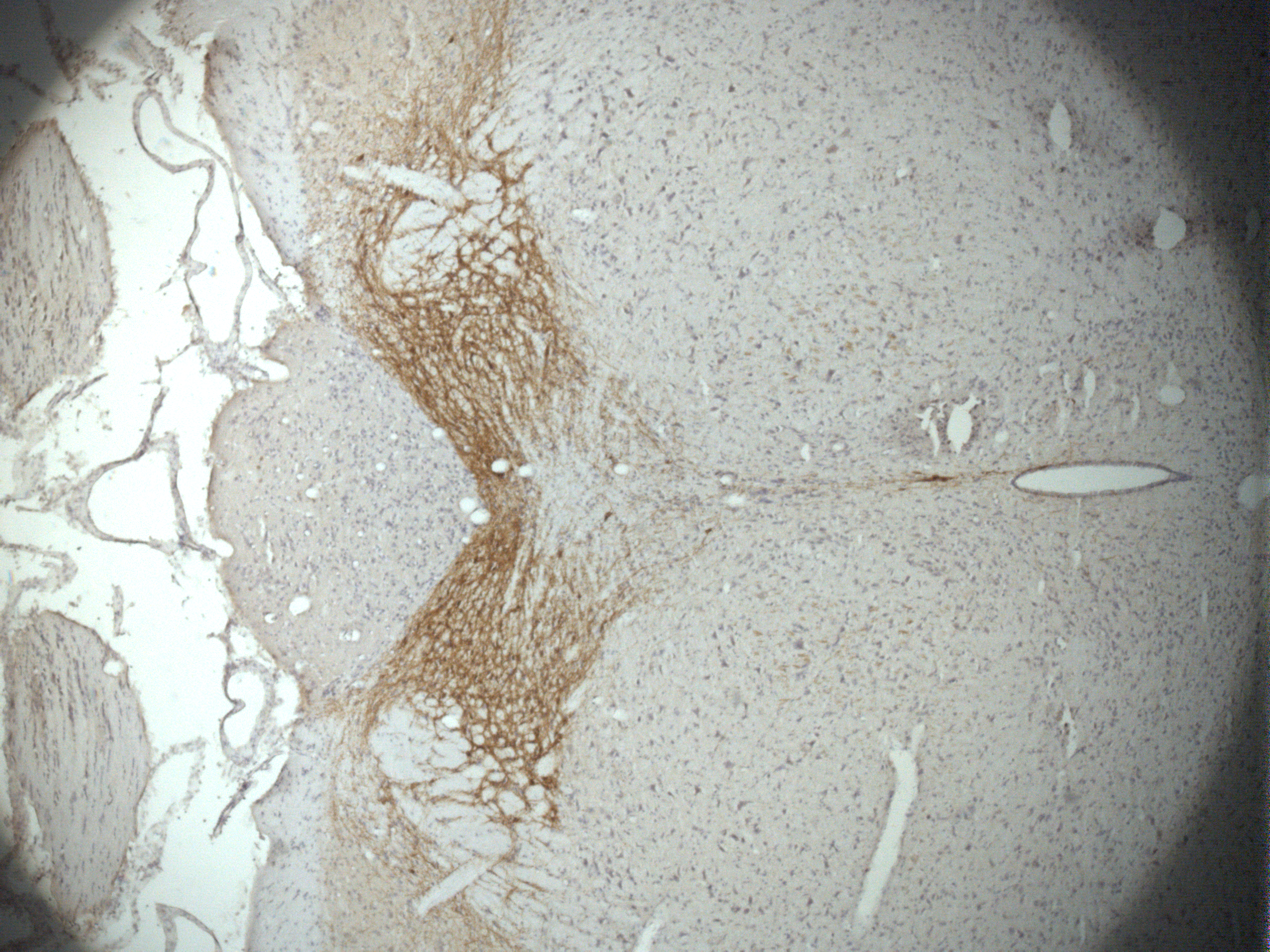

Supplement: S2 File — (ZIP) [file pone.0256488.s003.zip › S2 File/Figure 2A/Figure 2 MPTP Midbrain.tiff]

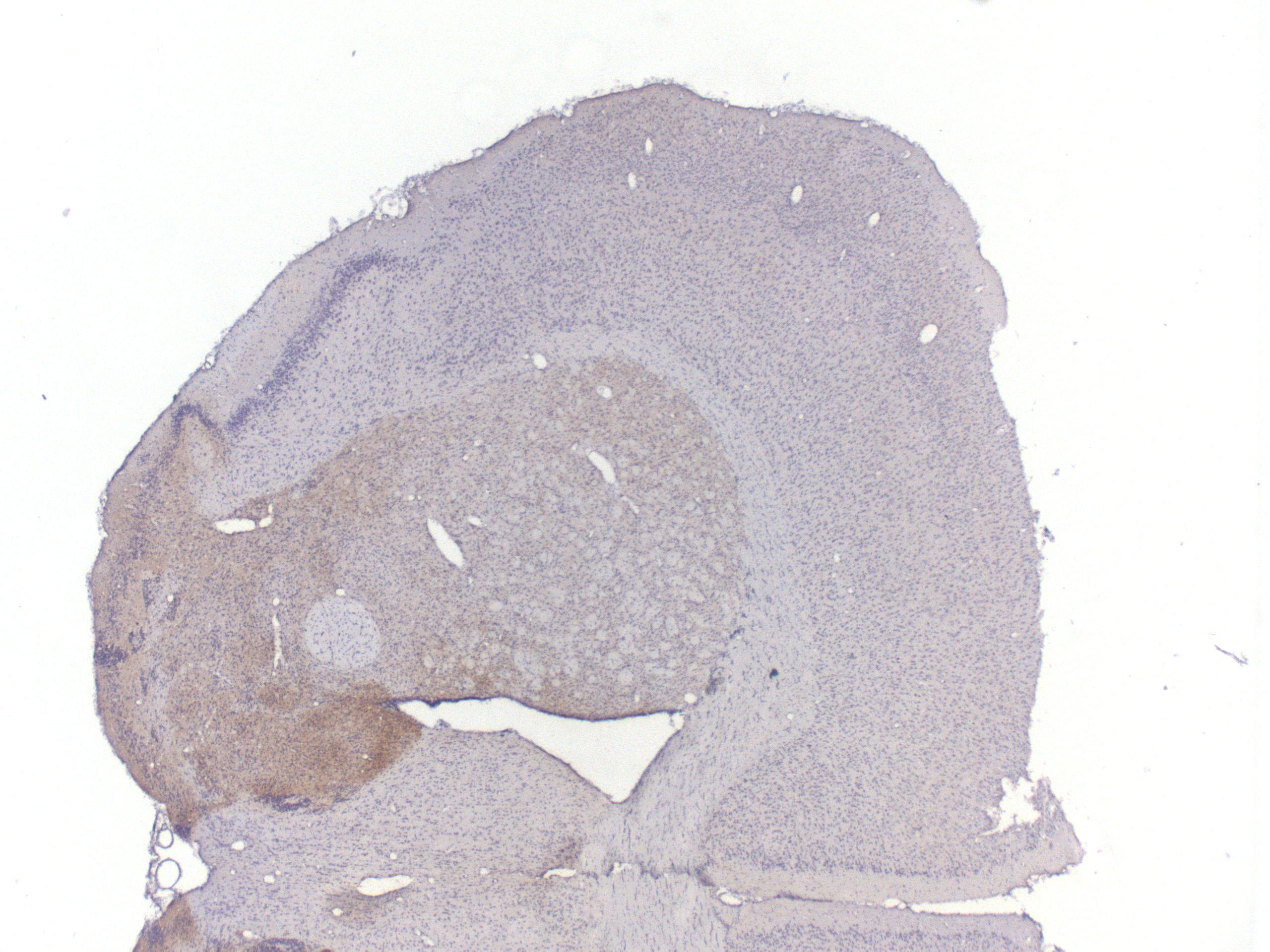

Supplement: S2 File — (ZIP) [file pone.0256488.s003.zip › S2 File/Figure 2A/Figure 2 MPTP Striatum.tiff]

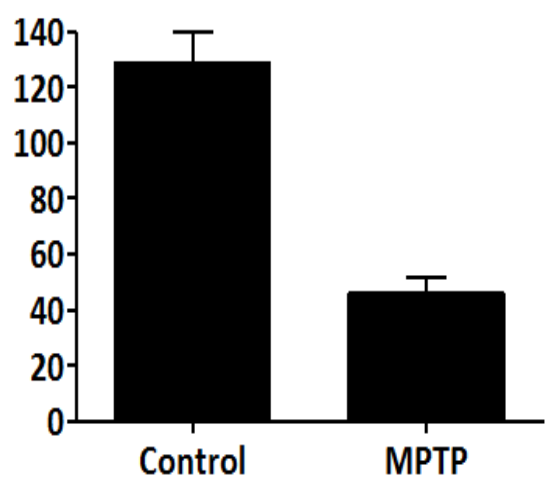

Supplement: S2 File — (ZIP) [file pone.0256488.s003.zip › S2 File/Figure 2B/Graph1.tif]

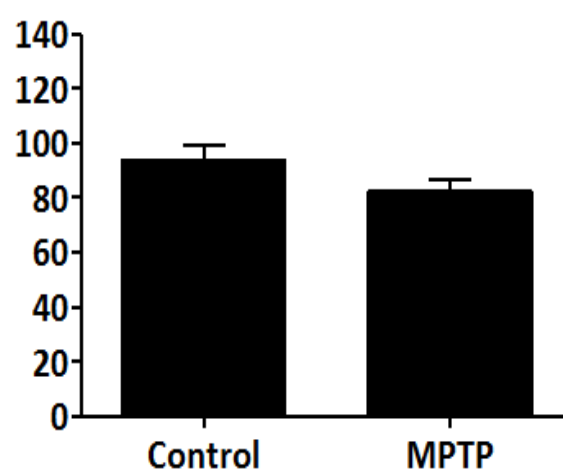

Supplement: S2 File — (ZIP) [file pone.0256488.s003.zip › S2 File/Figure 2B/Graph2.tif]

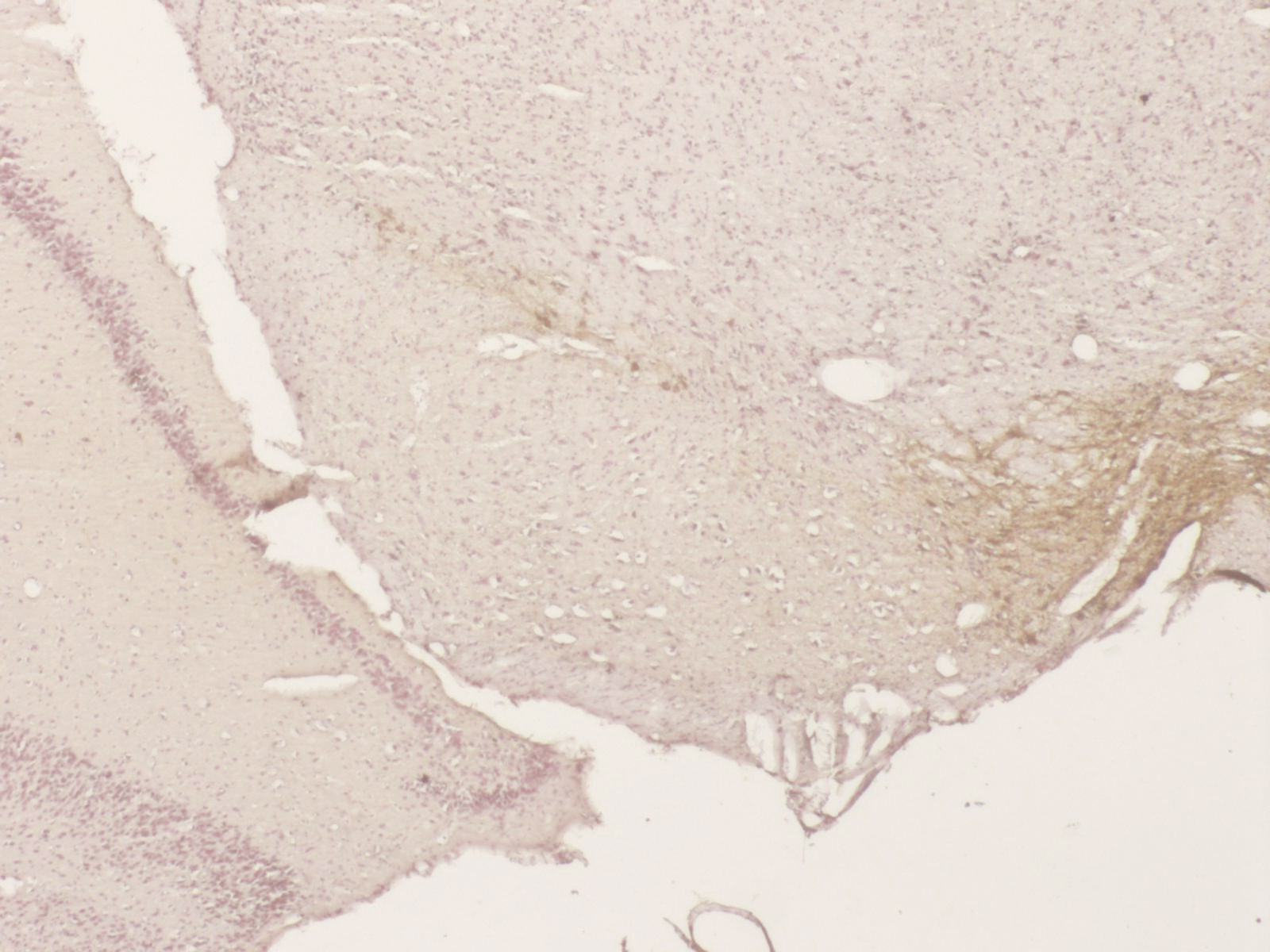

Supplement: S3 File — (ZIP) [file pone.0256488.s004.zip › S3 File/Figure 5A SNcp MPTP PBS.jpg]

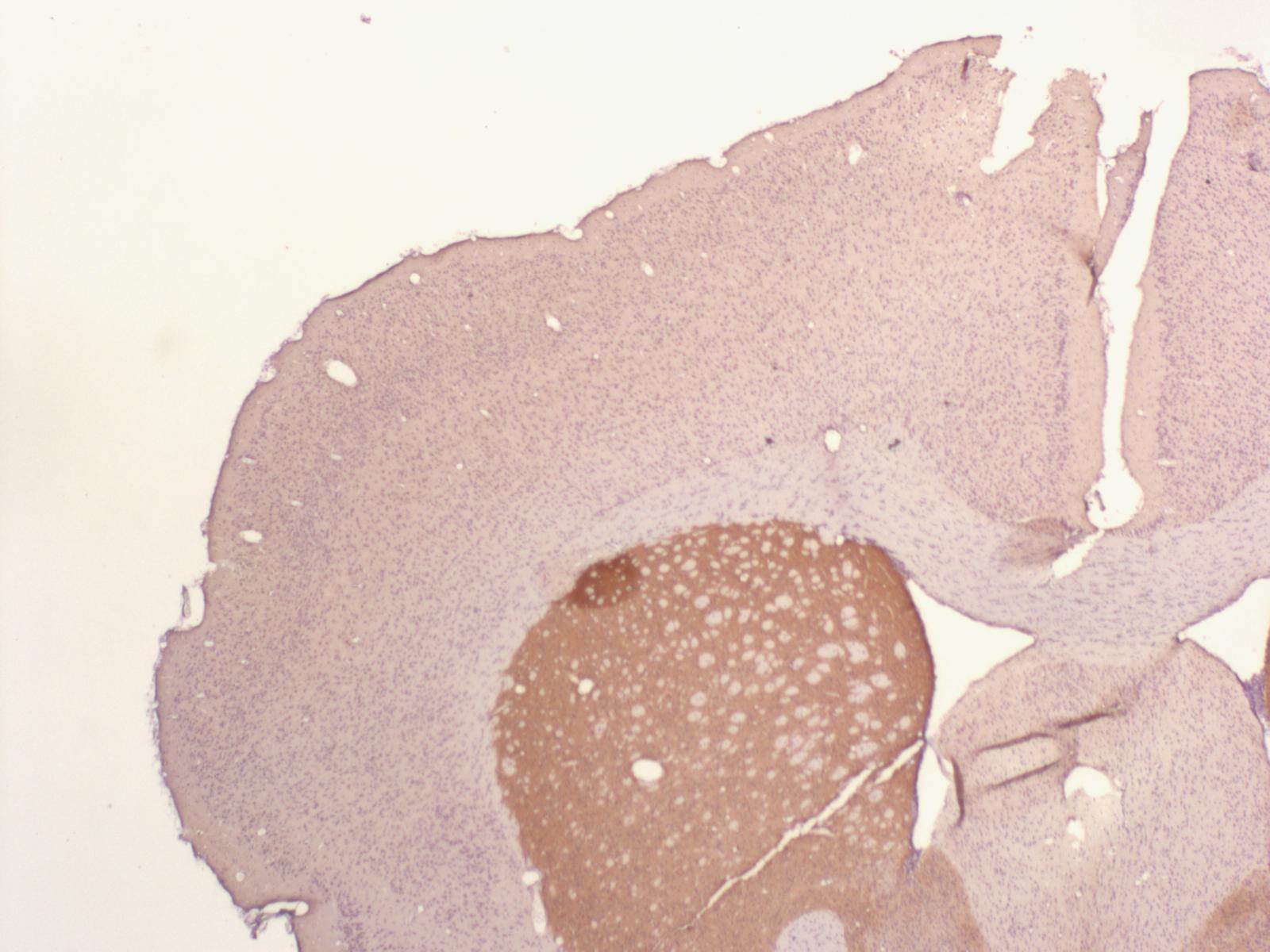

Supplement: S3 File — (ZIP) [file pone.0256488.s004.zip › S3 File/Figure 5A Striatum control PBS PBS.jpg]

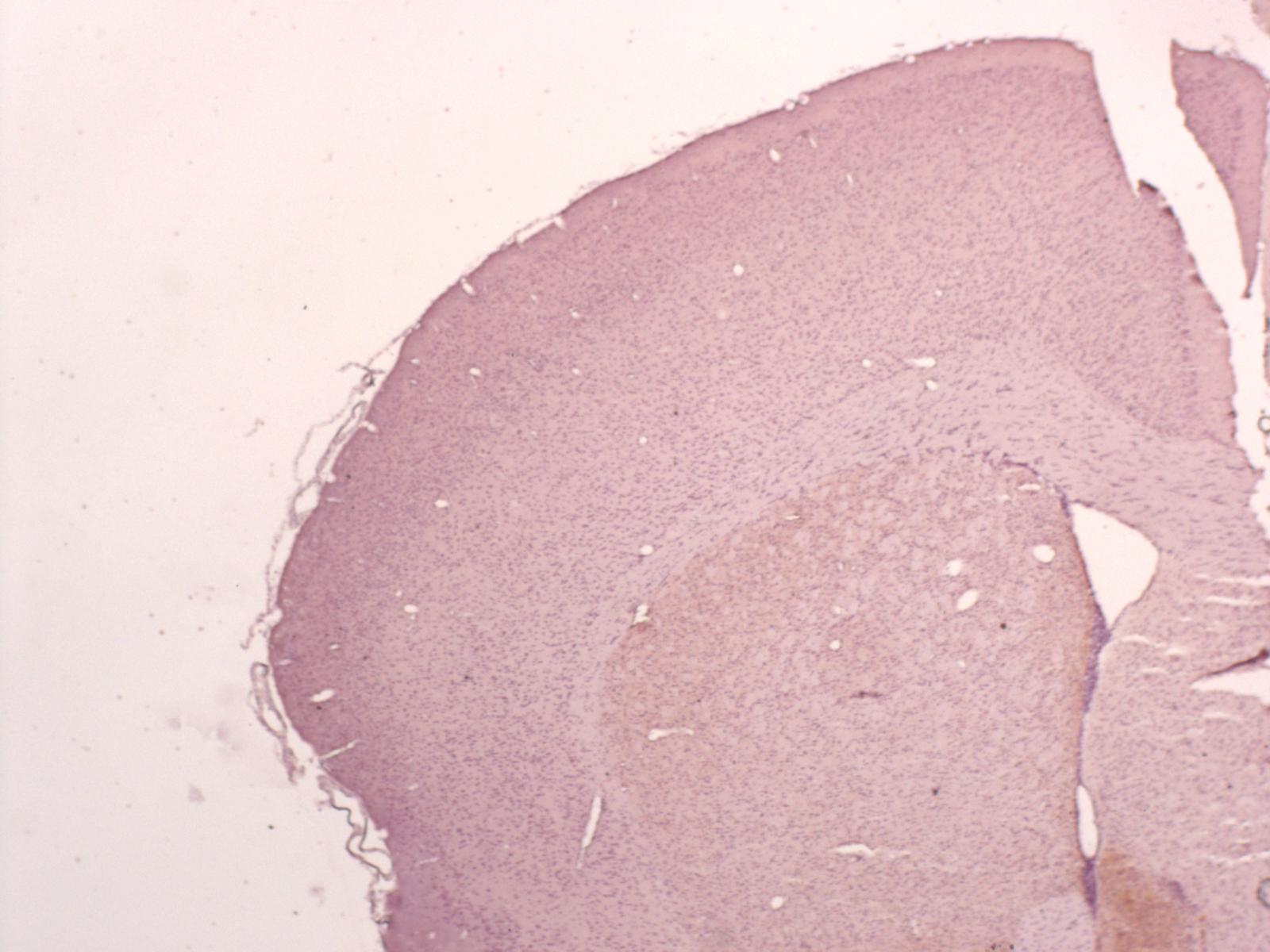

Supplement: S3 File — (ZIP) [file pone.0256488.s004.zip › S3 File/FIGURE 5A Striatum MPTP PBS.jpg]

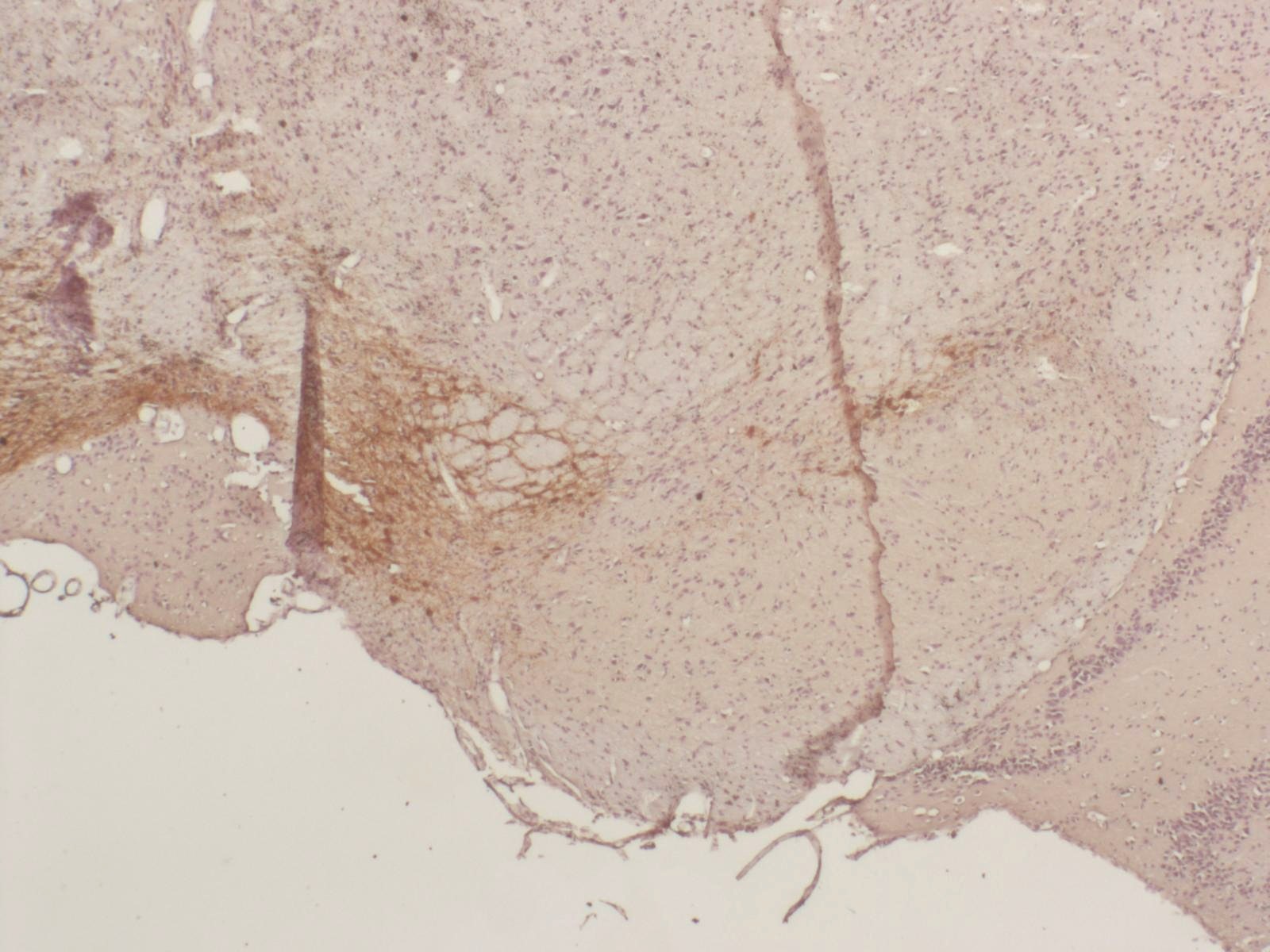

Supplement: S3 File — (ZIP) [file pone.0256488.s004.zip › S3 File/Figure 5B SNpc 28 days MPTP NCSC .jpg]

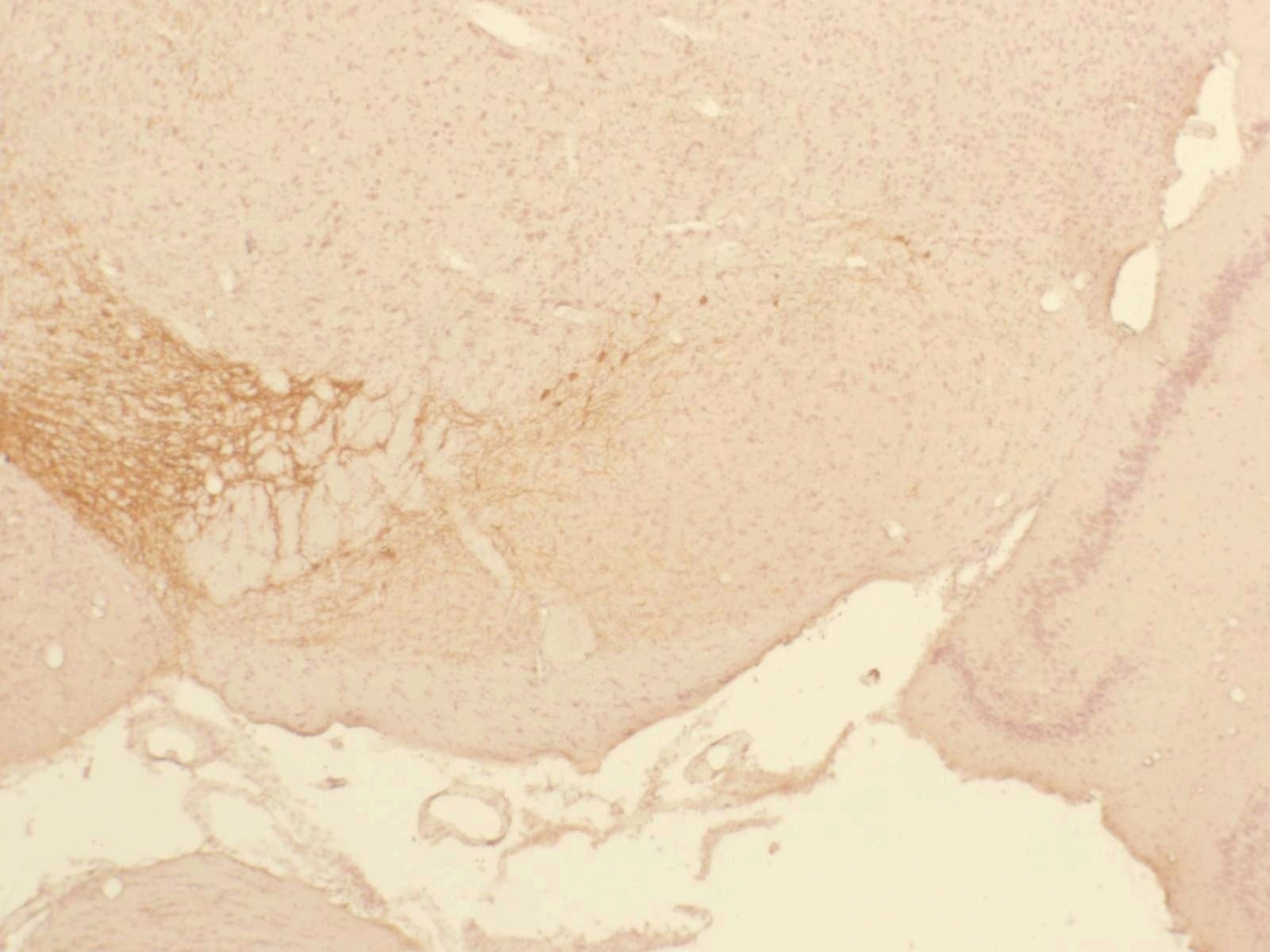

Supplement: S3 File — (ZIP) [file pone.0256488.s004.zip › S3 File/Figure 5B SNpc 70 days MPTP NCSC.jpg]

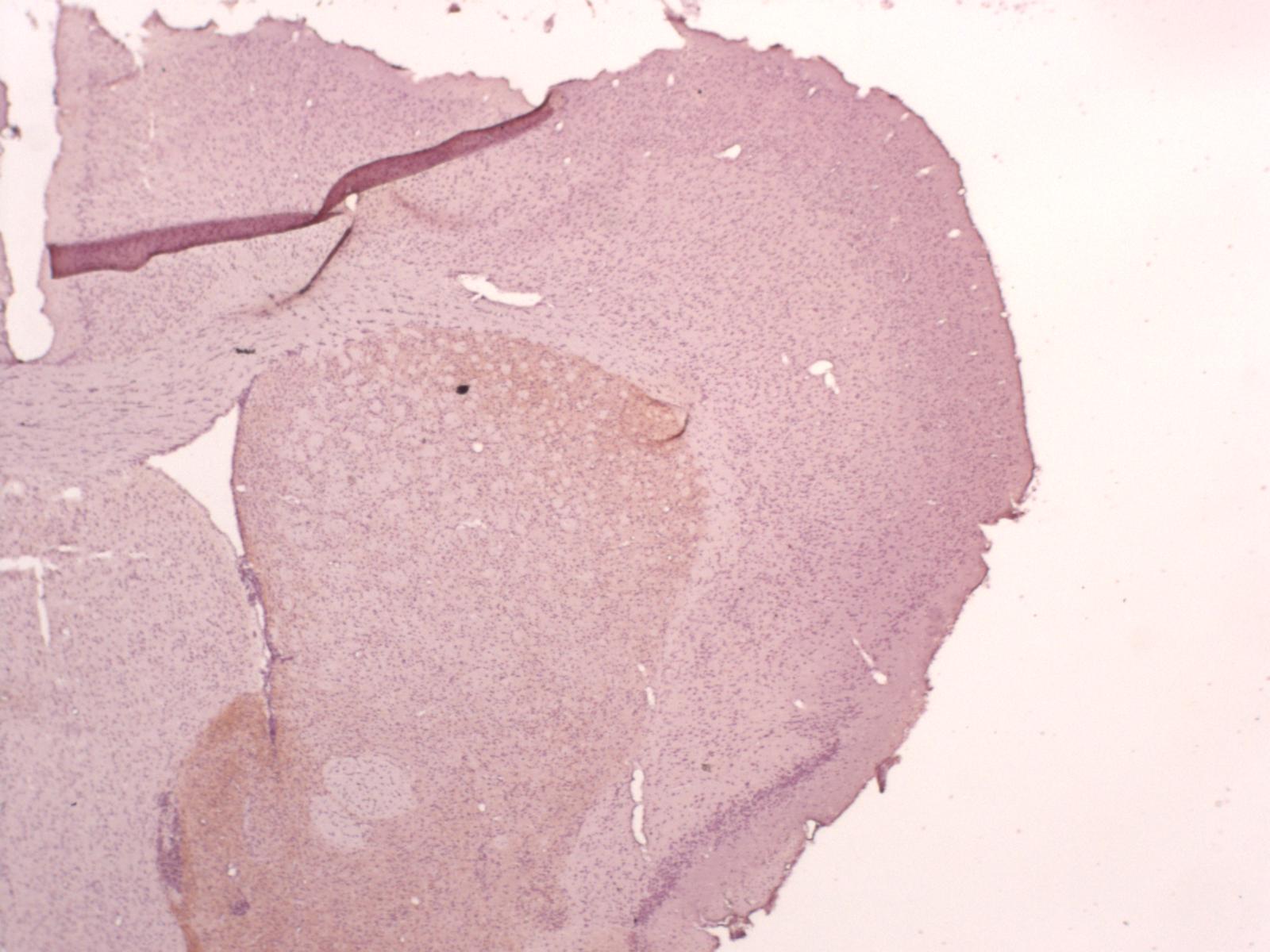

Supplement: S3 File — (ZIP) [file pone.0256488.s004.zip › S3 File/FIGURE 5B Striatum 28 Days MPTP NCSC.jpg]

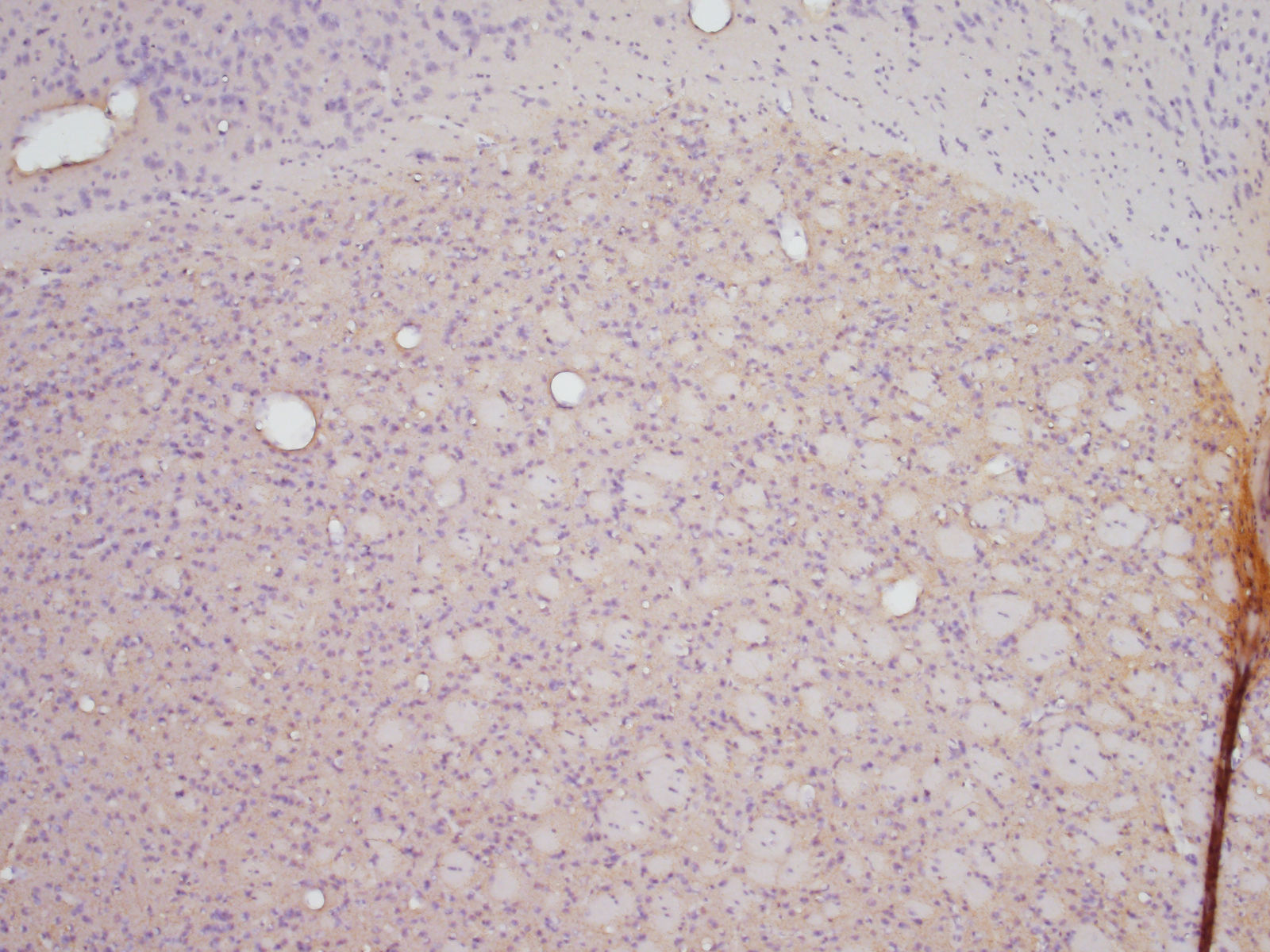

Supplement: S3 File — (ZIP) [file pone.0256488.s004.zip › S3 File/Figure 5B striatum 70 days MPTP NCSC.tif]

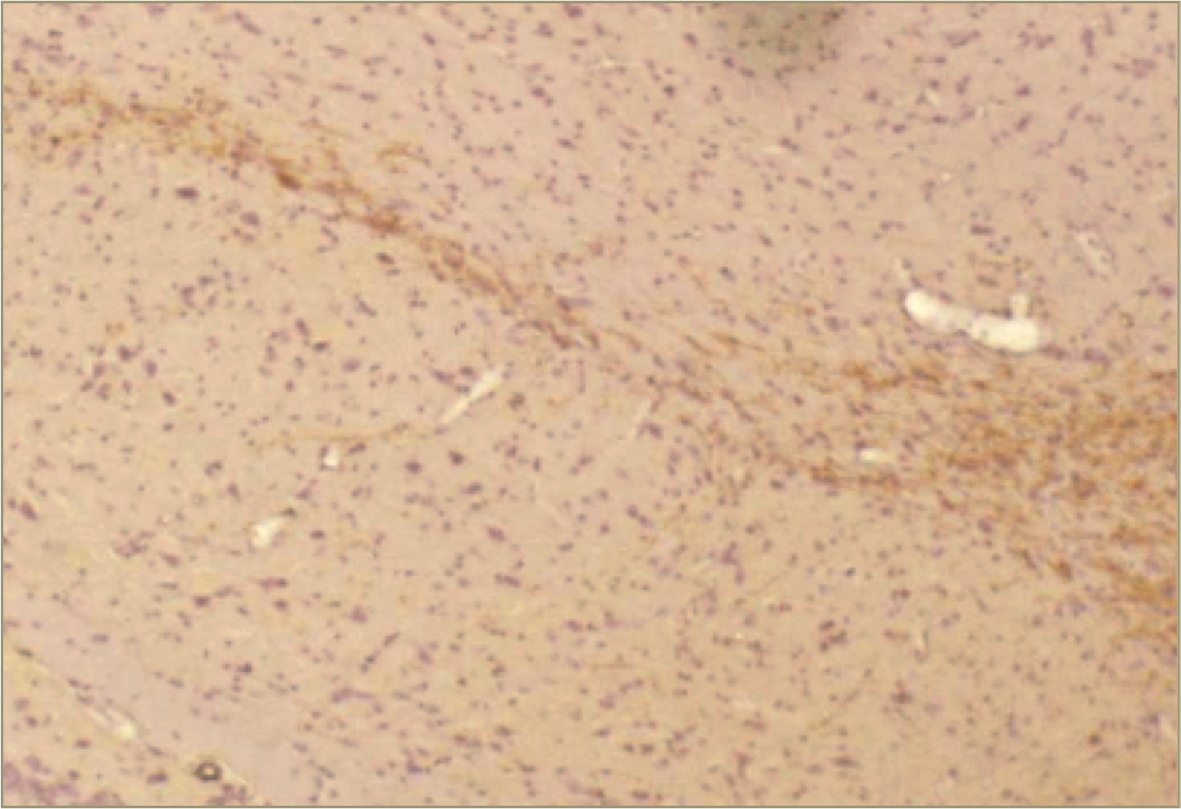

Supplement: S3 File — (ZIP) [file pone.0256488.s004.zip › S3 File/Figure 5C SNpc 28 days MSC.png]

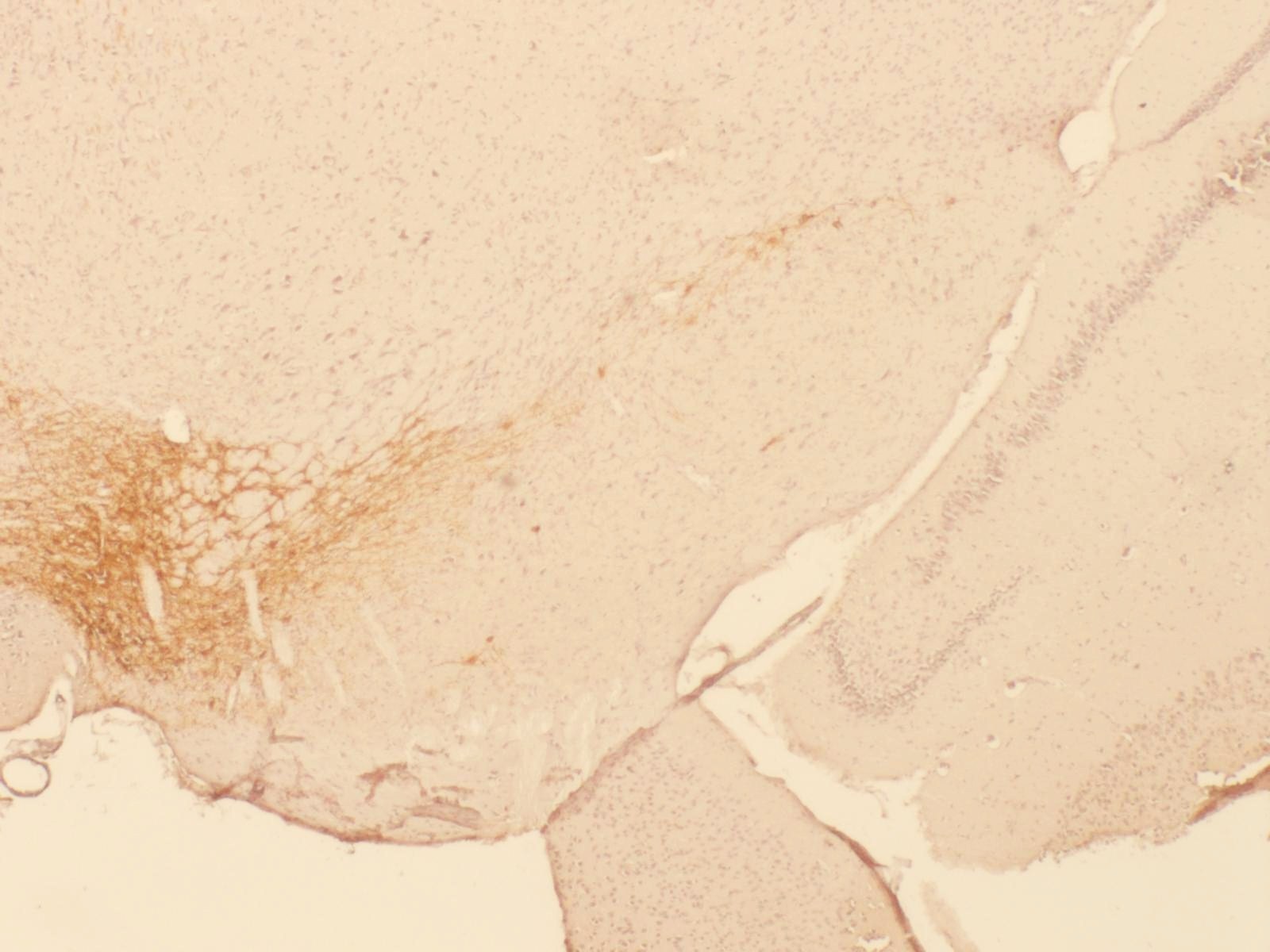

Supplement: S3 File — (ZIP) [file pone.0256488.s004.zip › S3 File/Figure 5C SNpc 70 days MPTP MSC.jpg]

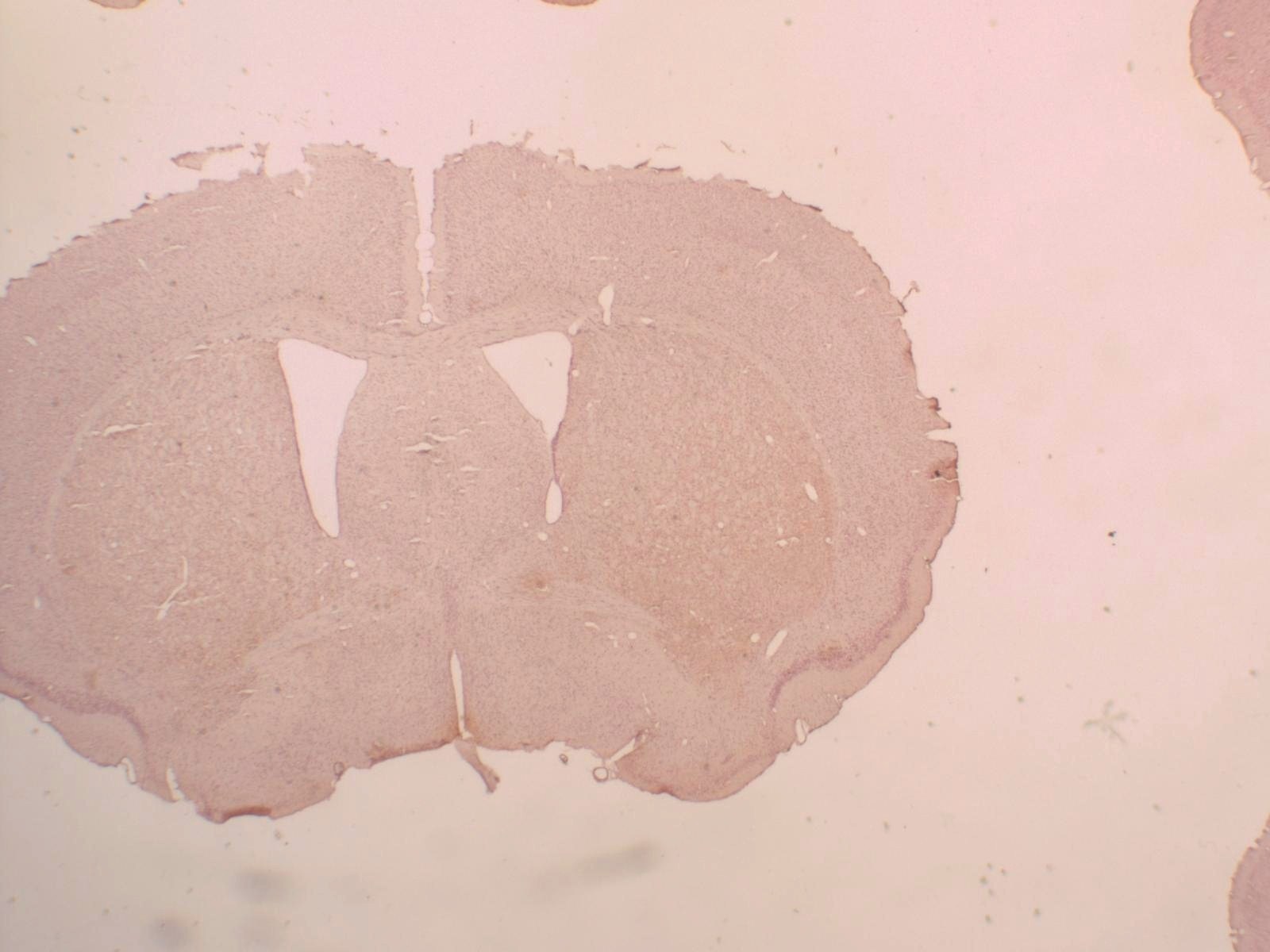

Supplement: S3 File — (ZIP) [file pone.0256488.s004.zip › S3 File/Figure 5C Striatum 28 days MPTP MSC.jpg]

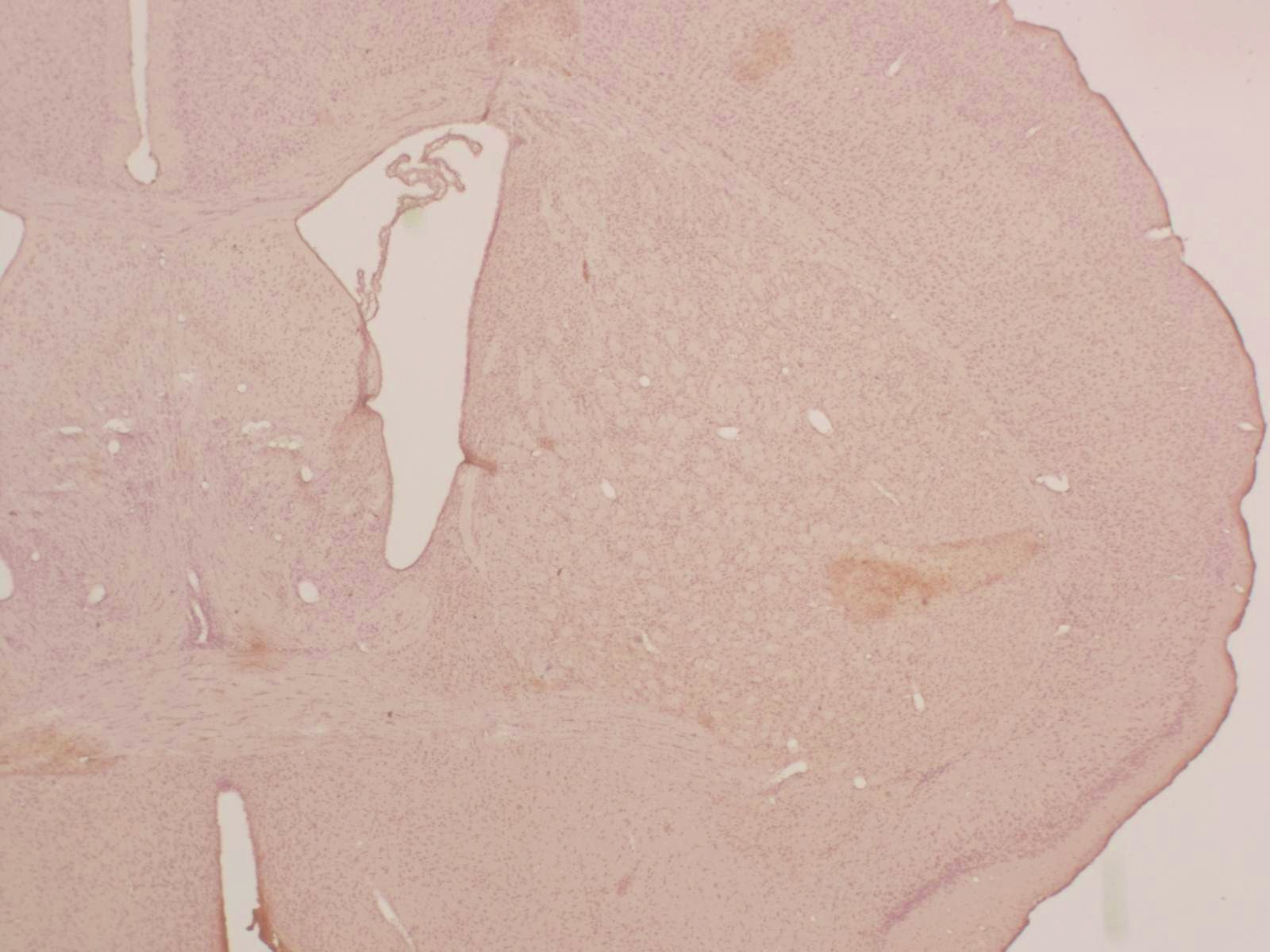

Supplement: S3 File — (ZIP) [file pone.0256488.s004.zip › S3 File/Figure 5C Striatum 70 days MPTP MSC.jpg]

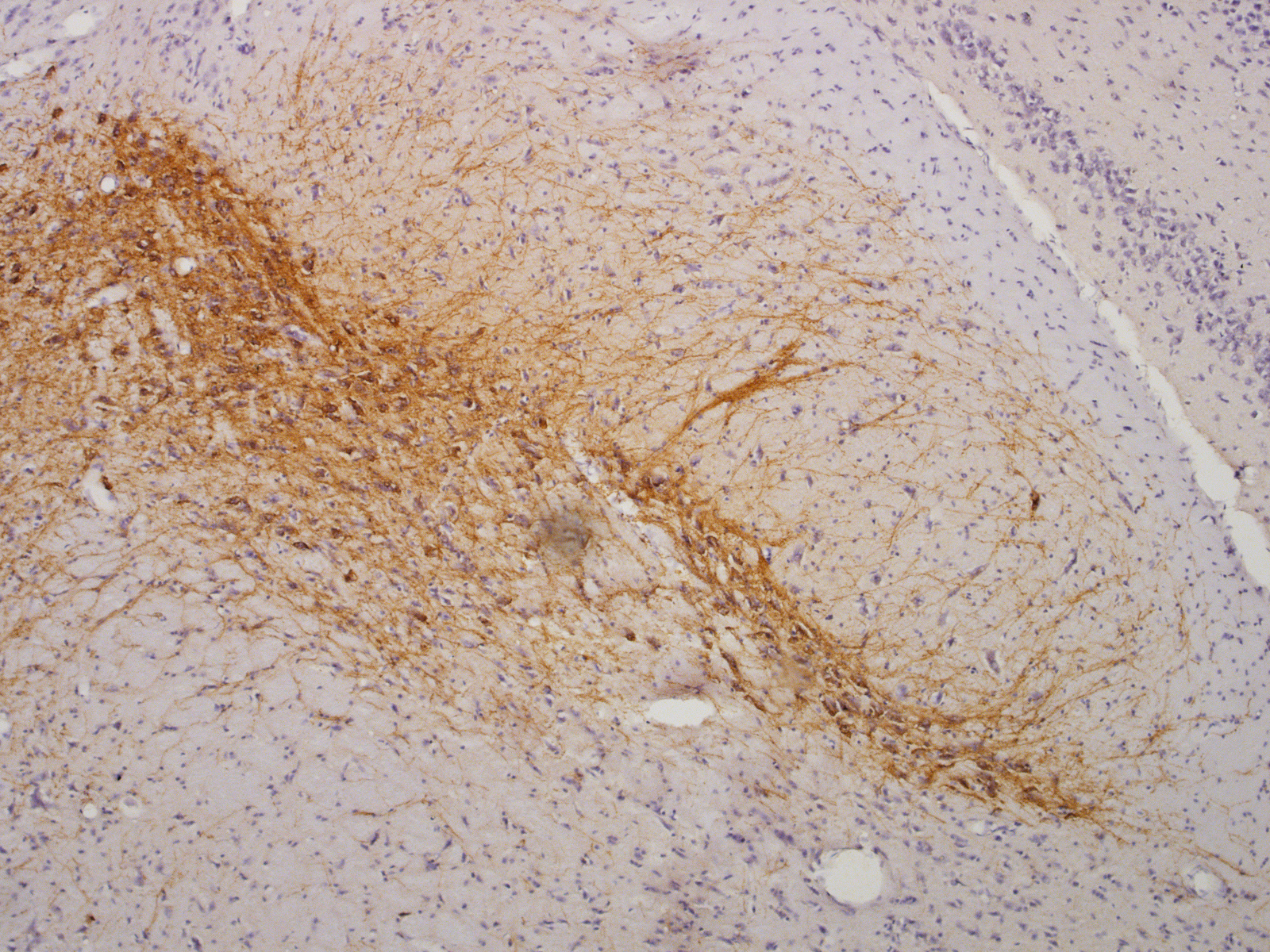

Supplement: S3 File — (ZIP) [file pone.0256488.s004.zip › S3 File/Figure 5A SNcp PBS PBS.jpg]

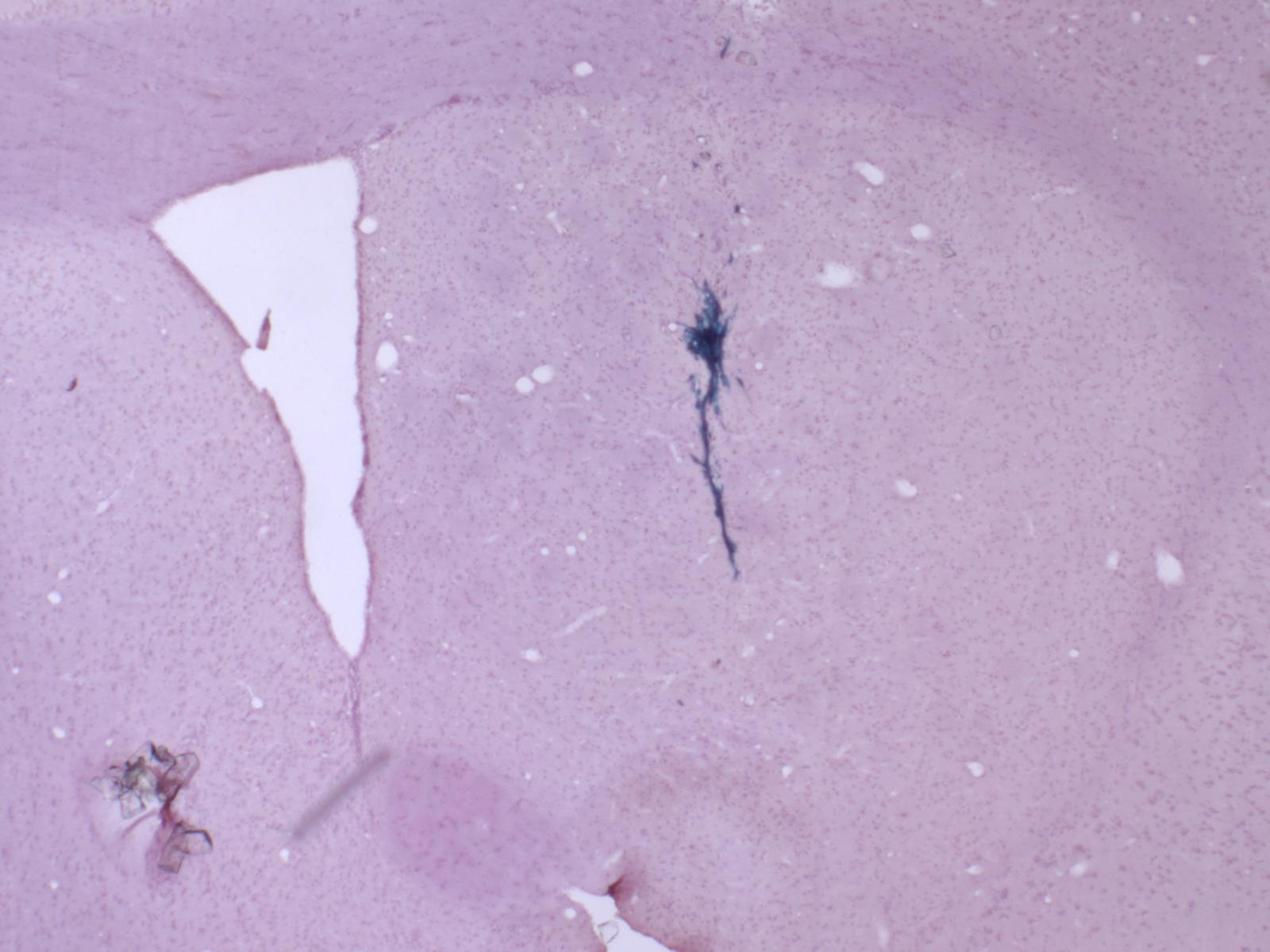

Supplement: S4 File — (ZIP) [file pone.0256488.s005.zip › S4 File/1 Figure S1A NCSC MPTP 3J .jpg]

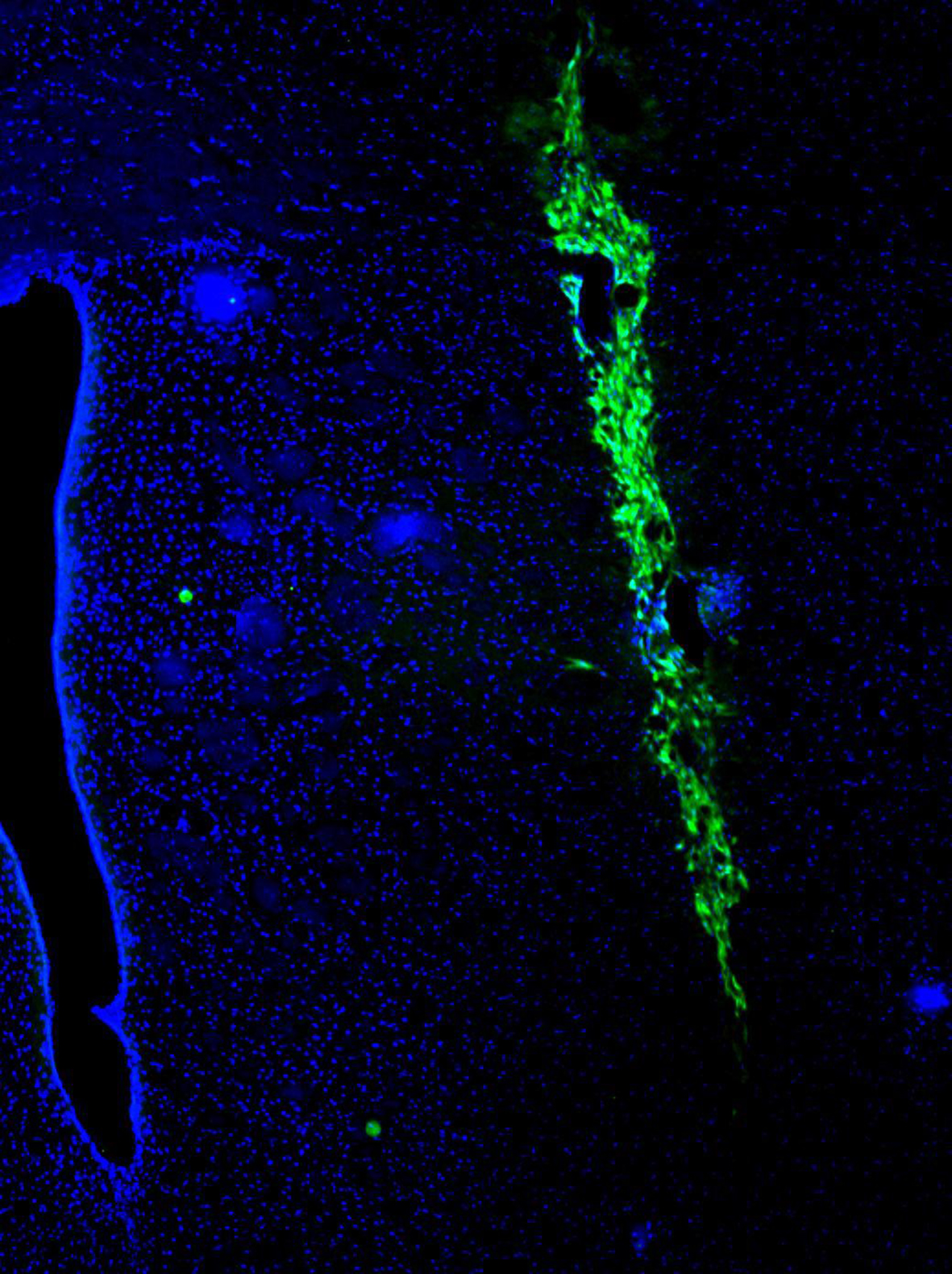

Supplement: S4 File — (ZIP) [file pone.0256488.s005.zip › S4 File/1 Figure S1B.jpg]

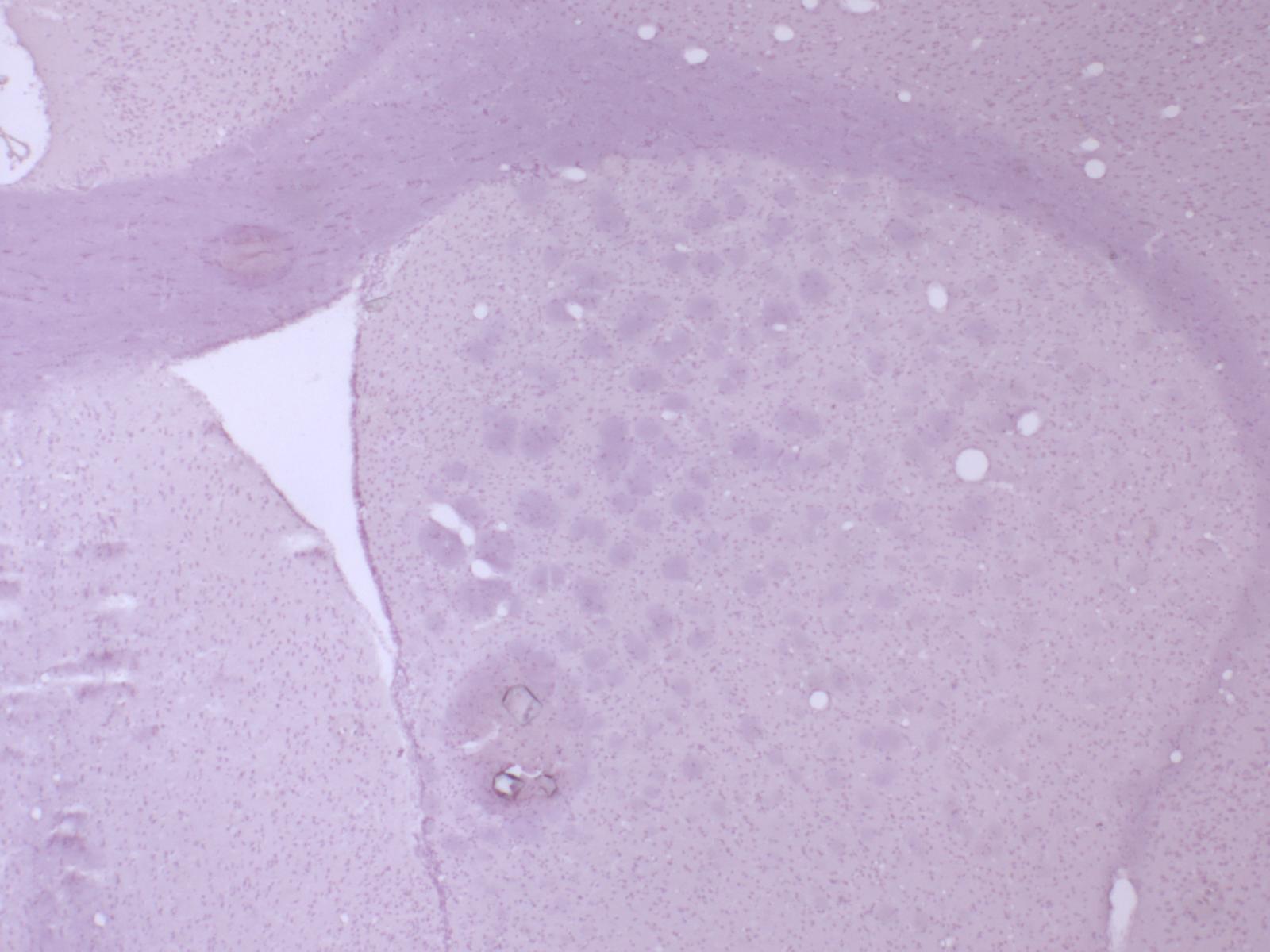

Supplement: S4 File — (ZIP) [file pone.0256488.s005.zip › S4 File/10 Figure S1A PBS NCSC 70J.jpg]

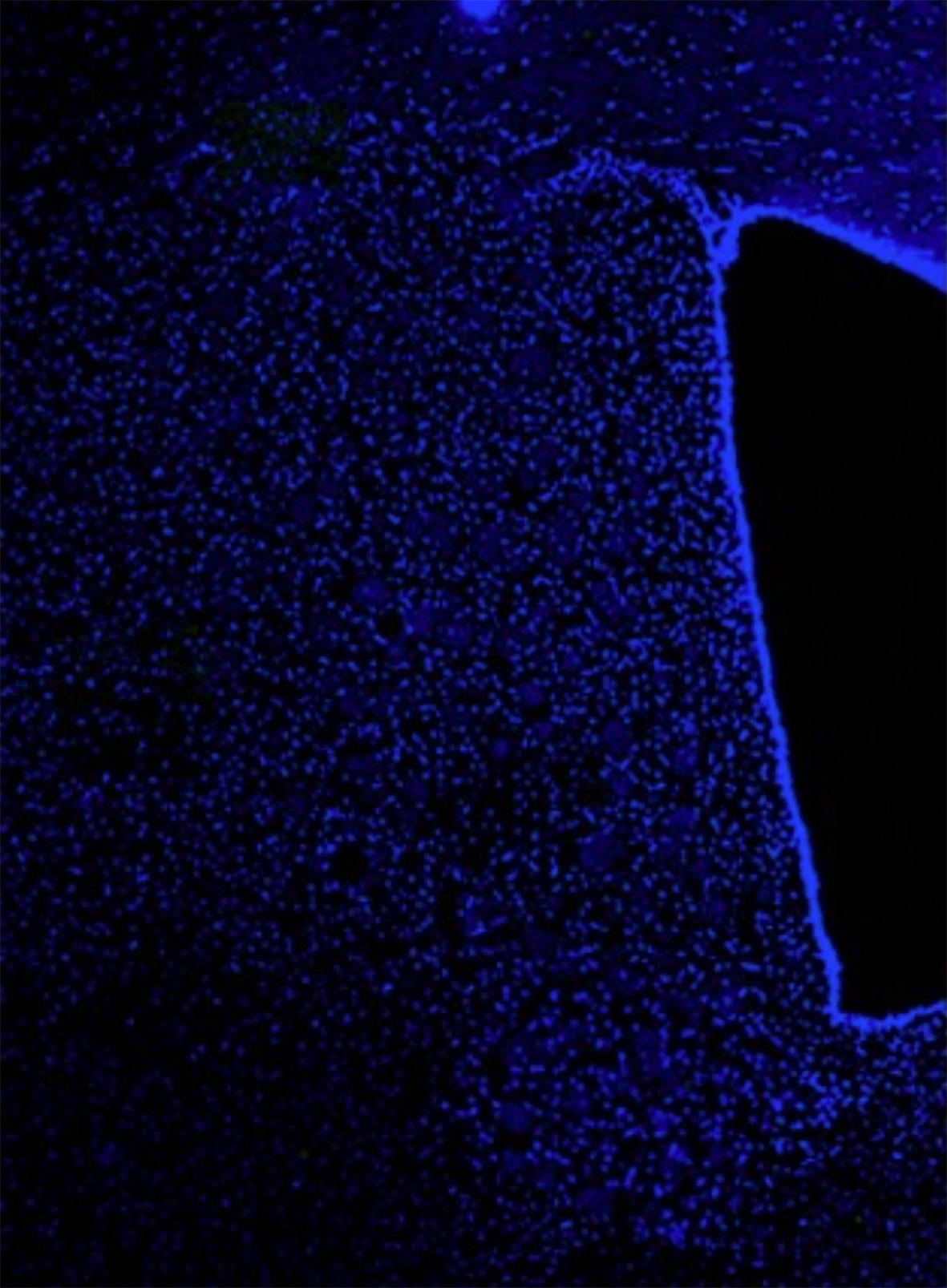

Supplement: S4 File — (ZIP) [file pone.0256488.s005.zip › S4 File/10 Figure S1B.jpg]

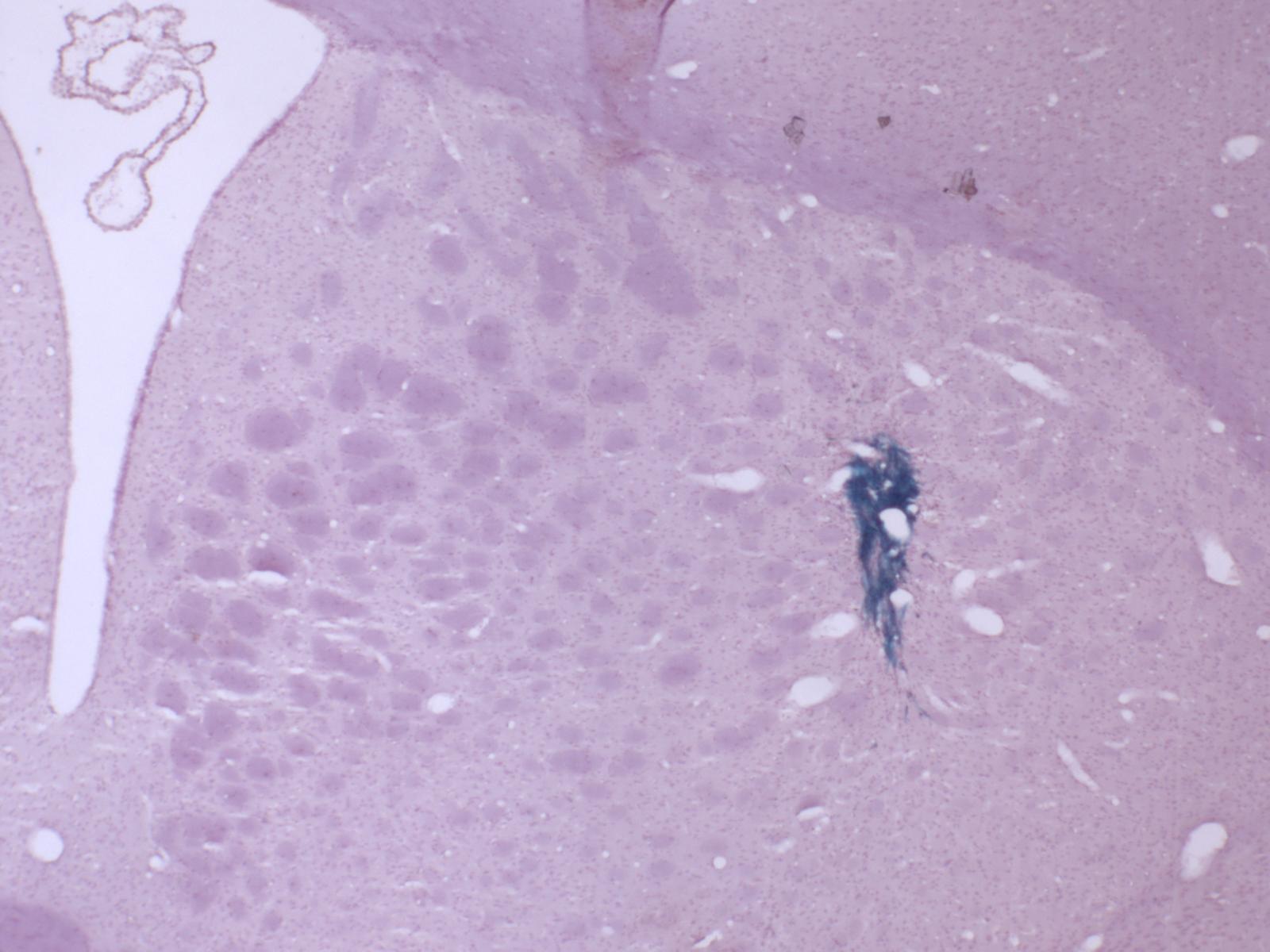

Supplement: S4 File — (ZIP) [file pone.0256488.s005.zip › S4 File/2 Figure S1A NCSC MPTP 7J.jpg]

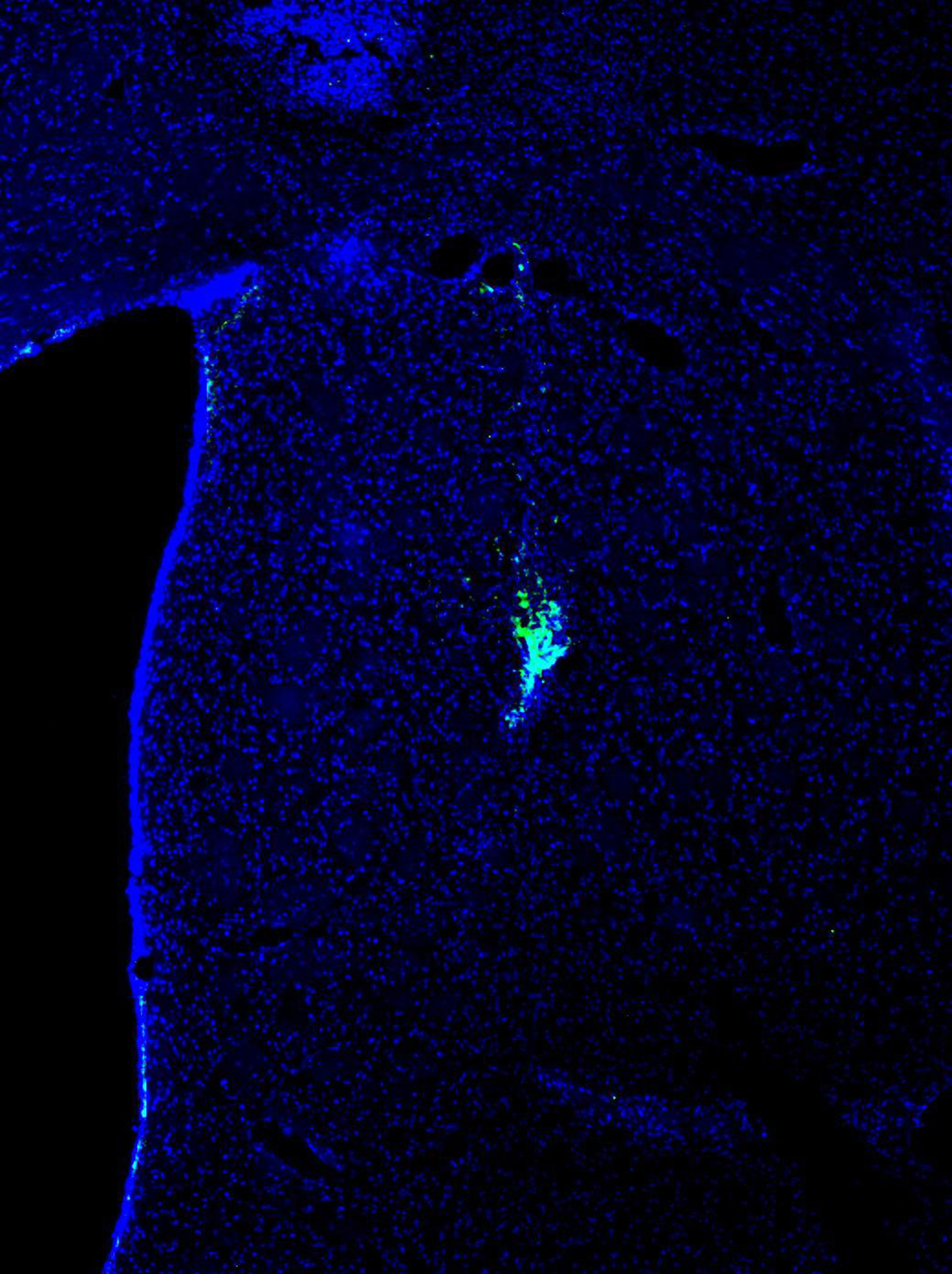

Supplement: S4 File — (ZIP) [file pone.0256488.s005.zip › S4 File/2 Figure S1B.jpg]

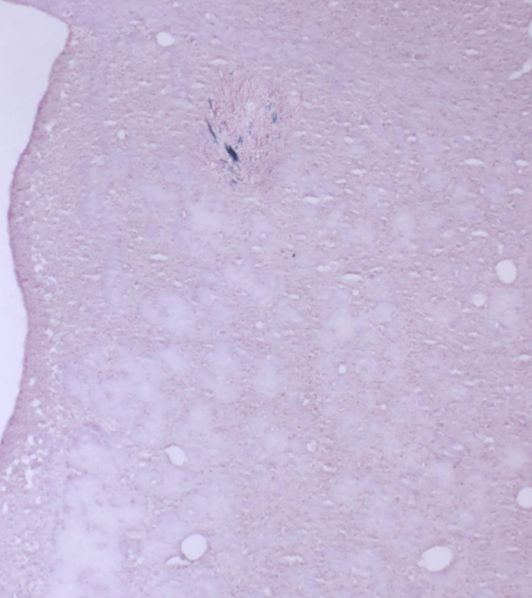

Supplement: S4 File — (ZIP) [file pone.0256488.s005.zip › S4 File/3 Figure S1A NCSC MPTP 14J.JPG]

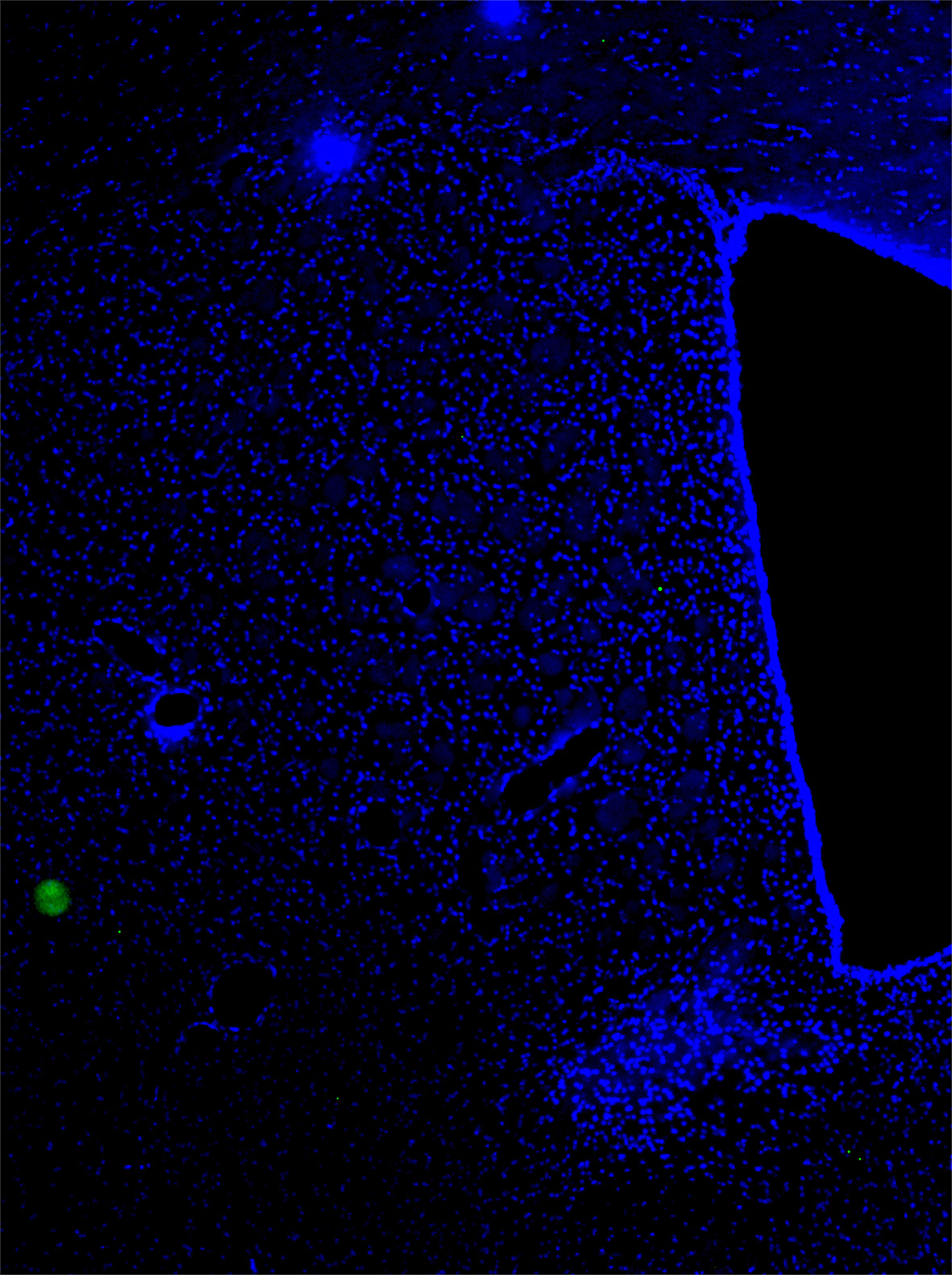

Supplement: S4 File — (ZIP) [file pone.0256488.s005.zip › S4 File/3 Figure S1B.jpg]

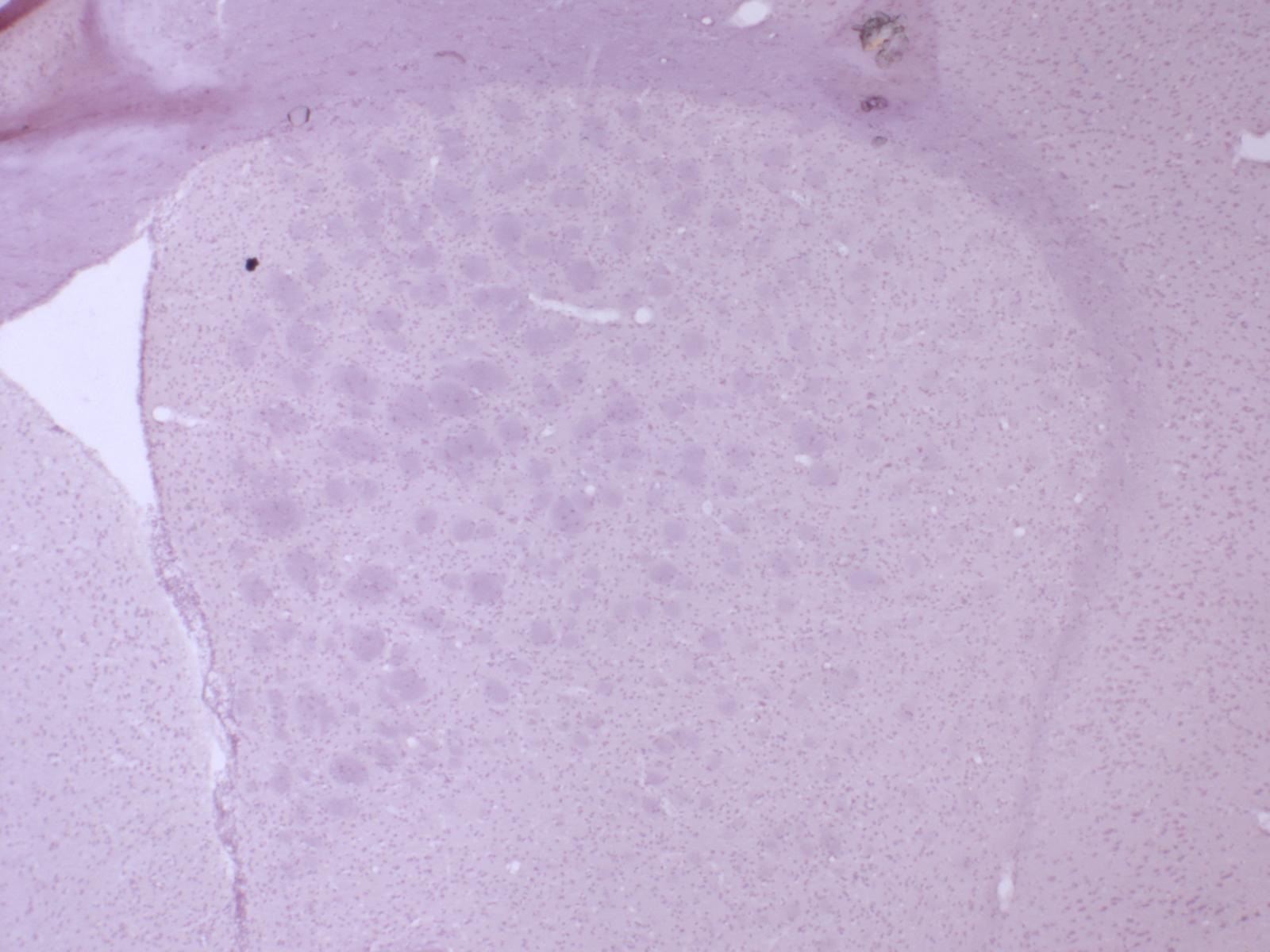

Supplement: S4 File — (ZIP) [file pone.0256488.s005.zip › S4 File/4 Figure S1A NCSC MPTP 28J.jpg]

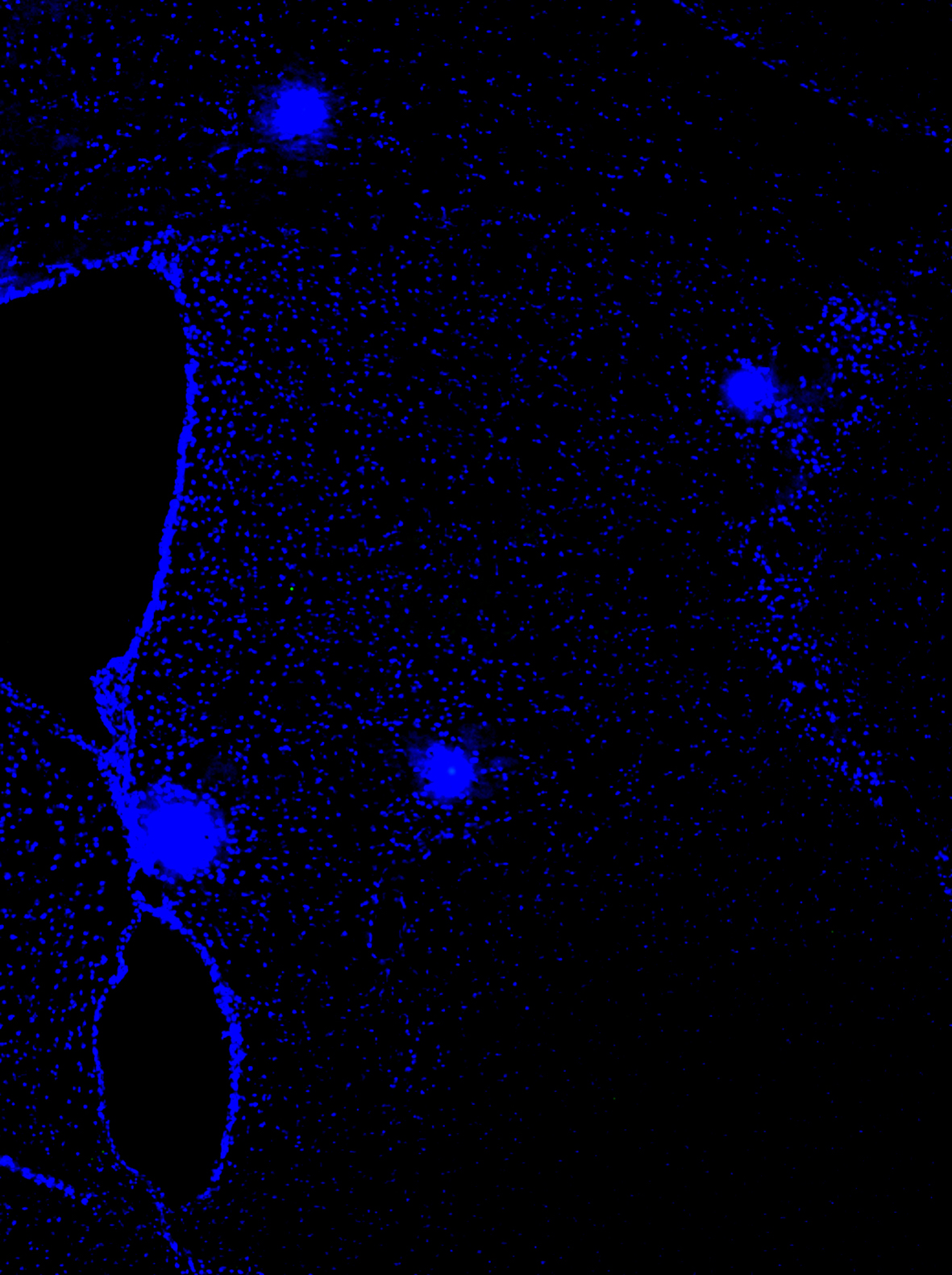

Supplement: S4 File — (ZIP) [file pone.0256488.s005.zip › S4 File/4 Figure S1B.jpg]

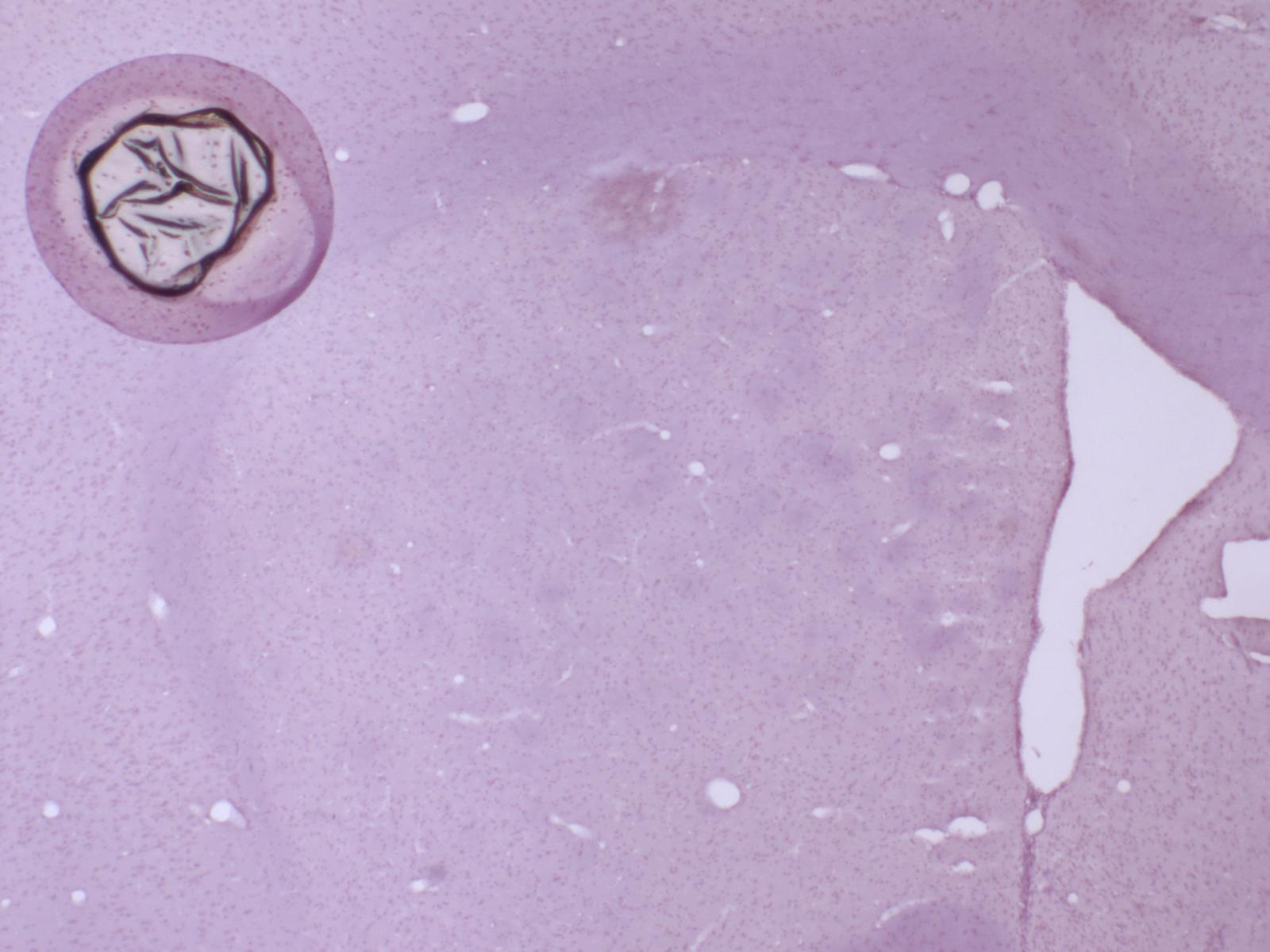

Supplement: S4 File — (ZIP) [file pone.0256488.s005.zip › S4 File/5 Figure S1A NCSC MPTP 70J.jpg]

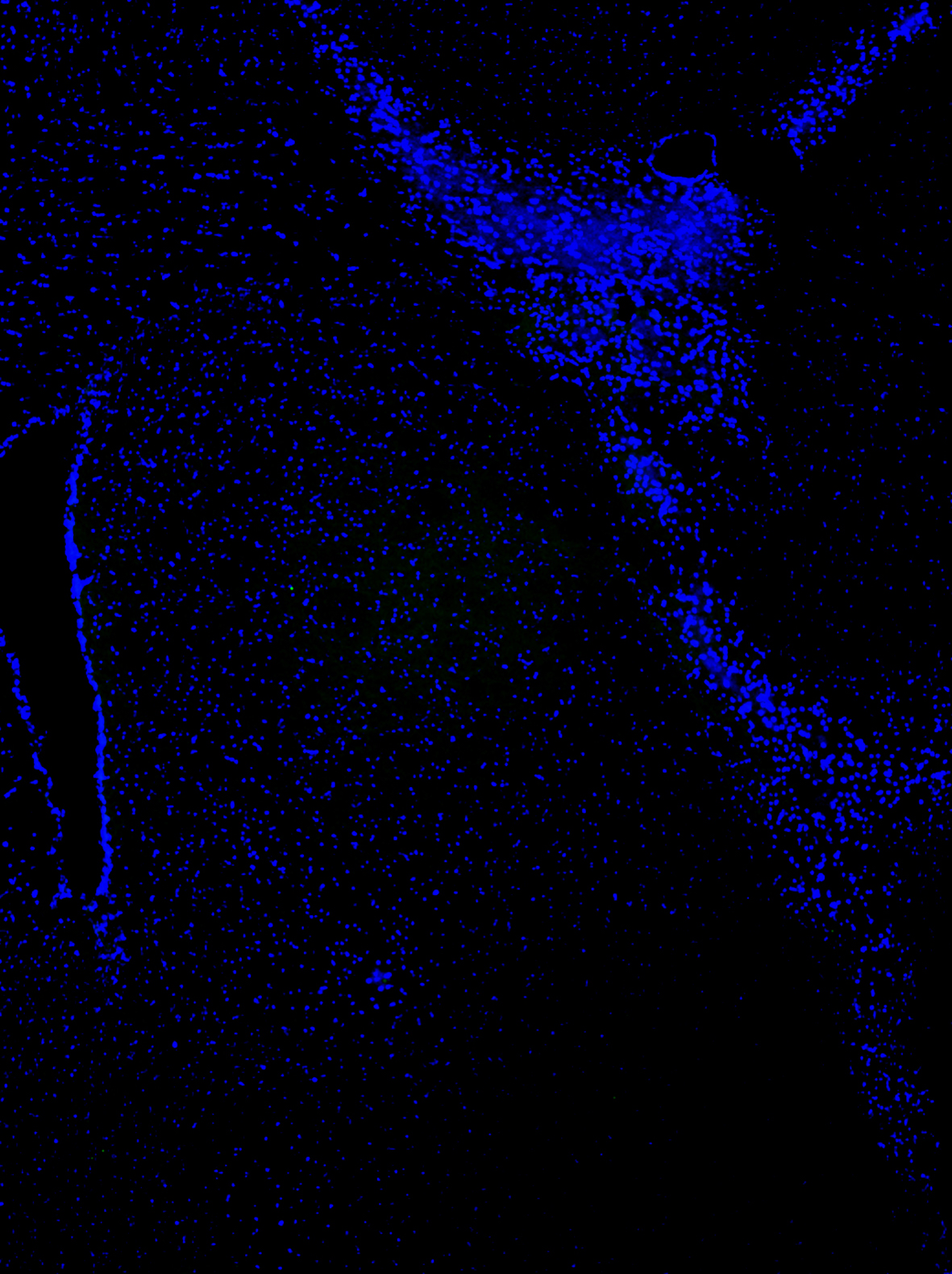

Supplement: S4 File — (ZIP) [file pone.0256488.s005.zip › S4 File/5 Figure S1B.jpg]

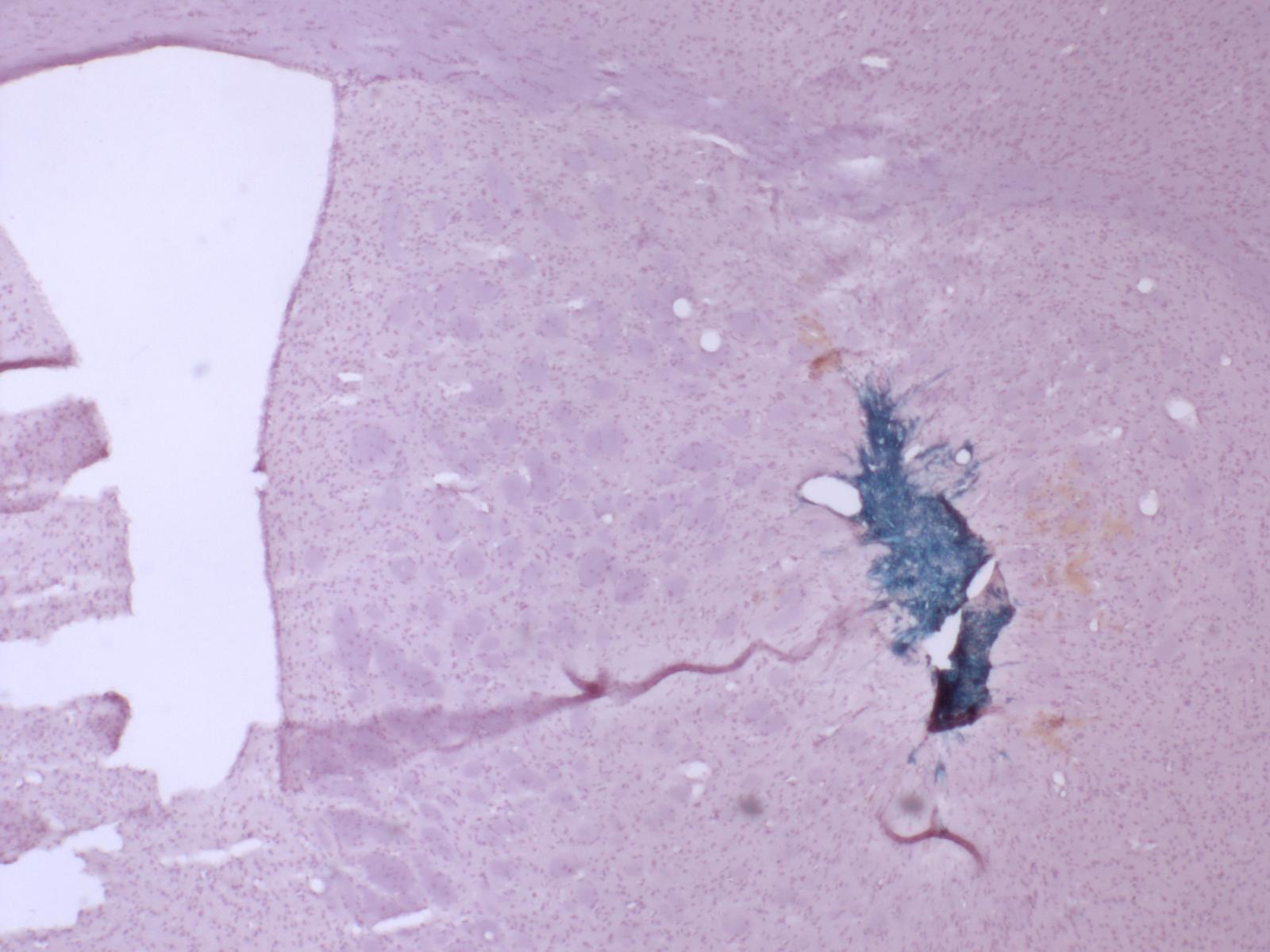

Supplement: S4 File — (ZIP) [file pone.0256488.s005.zip › S4 File/6 Figure S1A NCSC PBS 3J.jpg]

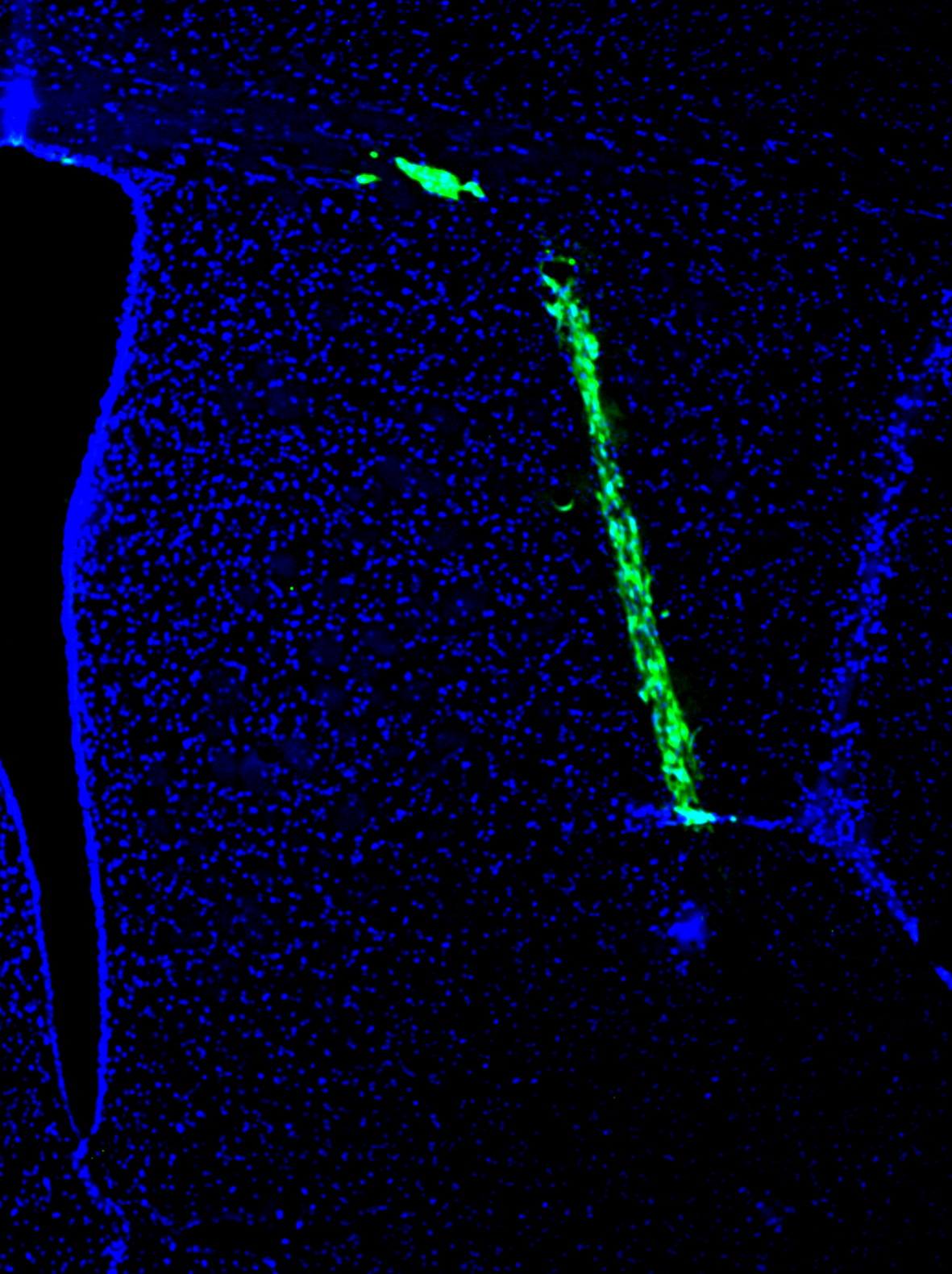

Supplement: S4 File — (ZIP) [file pone.0256488.s005.zip › S4 File/6 Figure S1B.jpg]

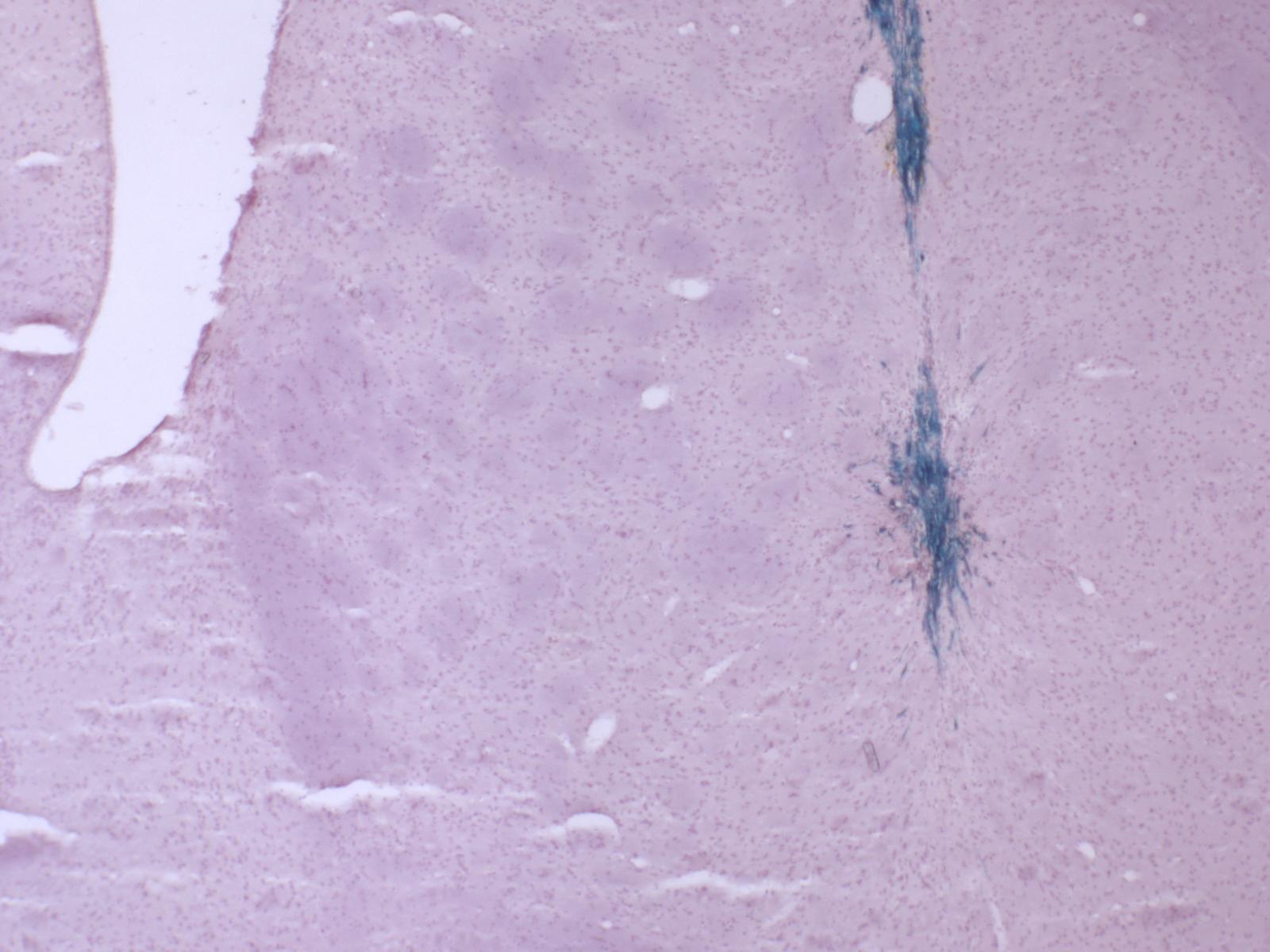

Supplement: S4 File — (ZIP) [file pone.0256488.s005.zip › S4 File/7 Figure S1A PBS NCSC 7J.jpg]

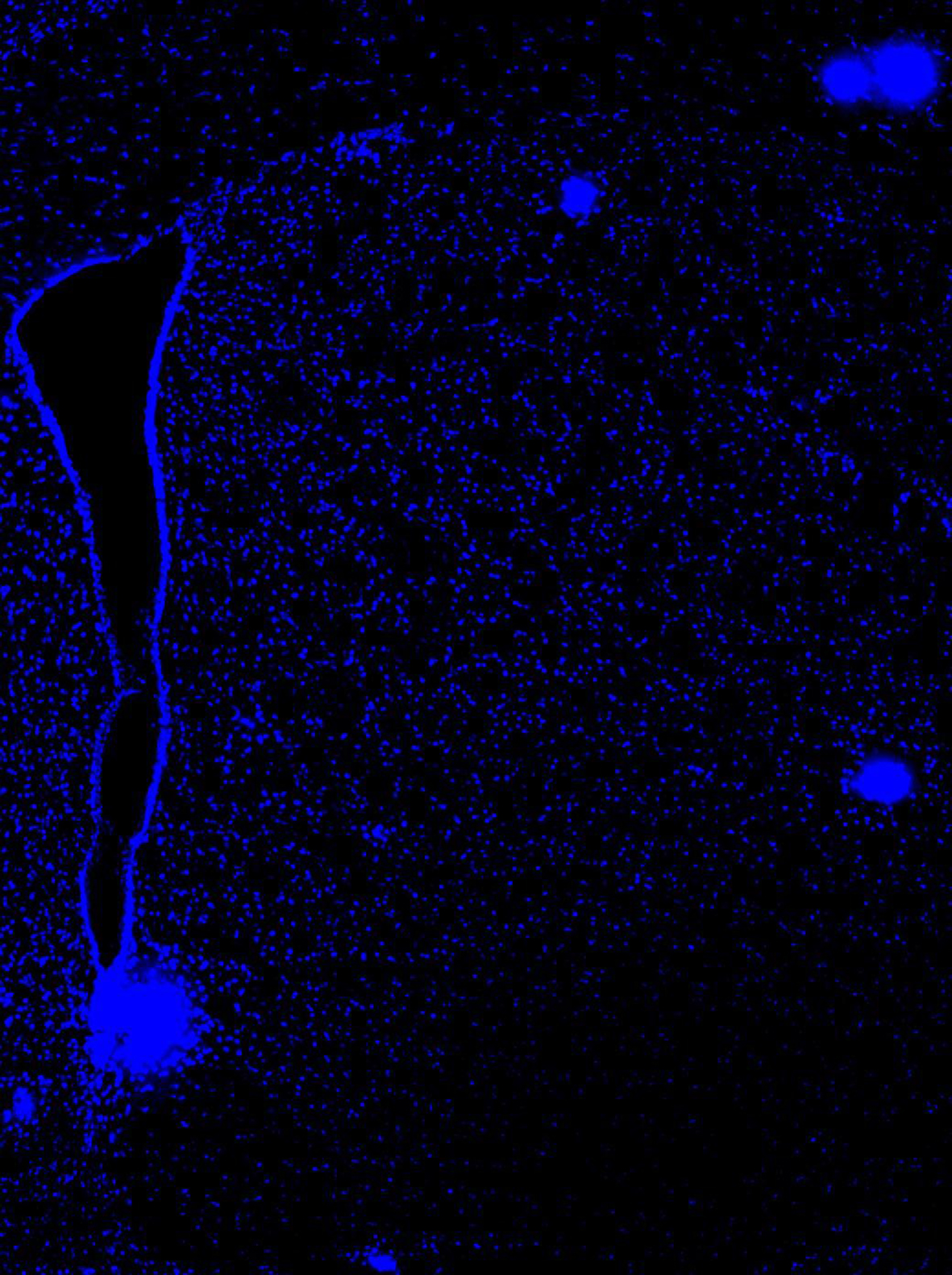

Supplement: S4 File — (ZIP) [file pone.0256488.s005.zip › S4 File/7 Figure S1B.jpg]

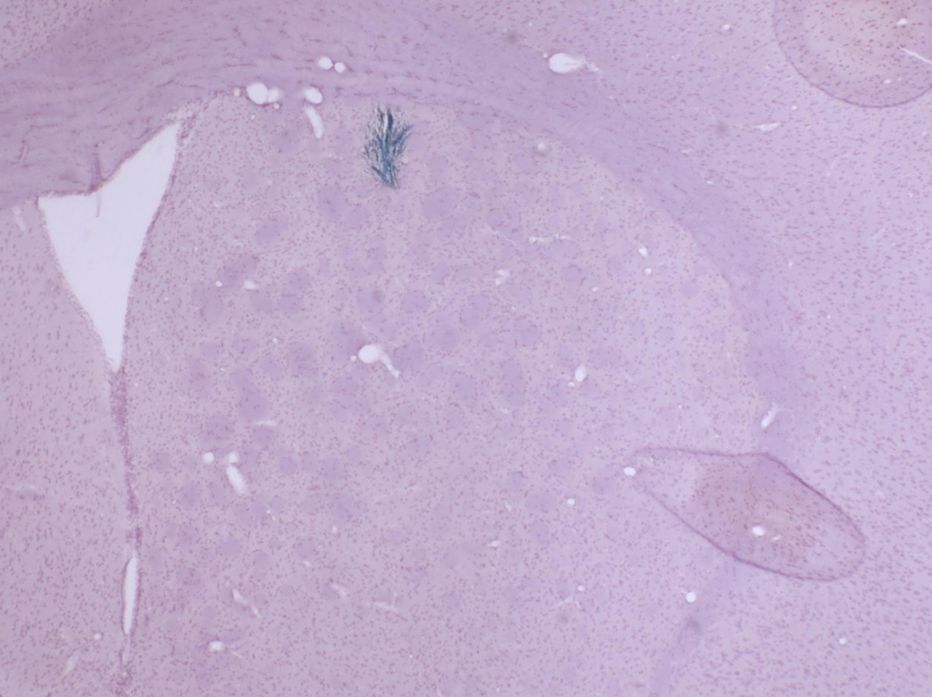

Supplement: S4 File — (ZIP) [file pone.0256488.s005.zip › S4 File/8 Figure S1A NCSC PBS 14J.JPG]

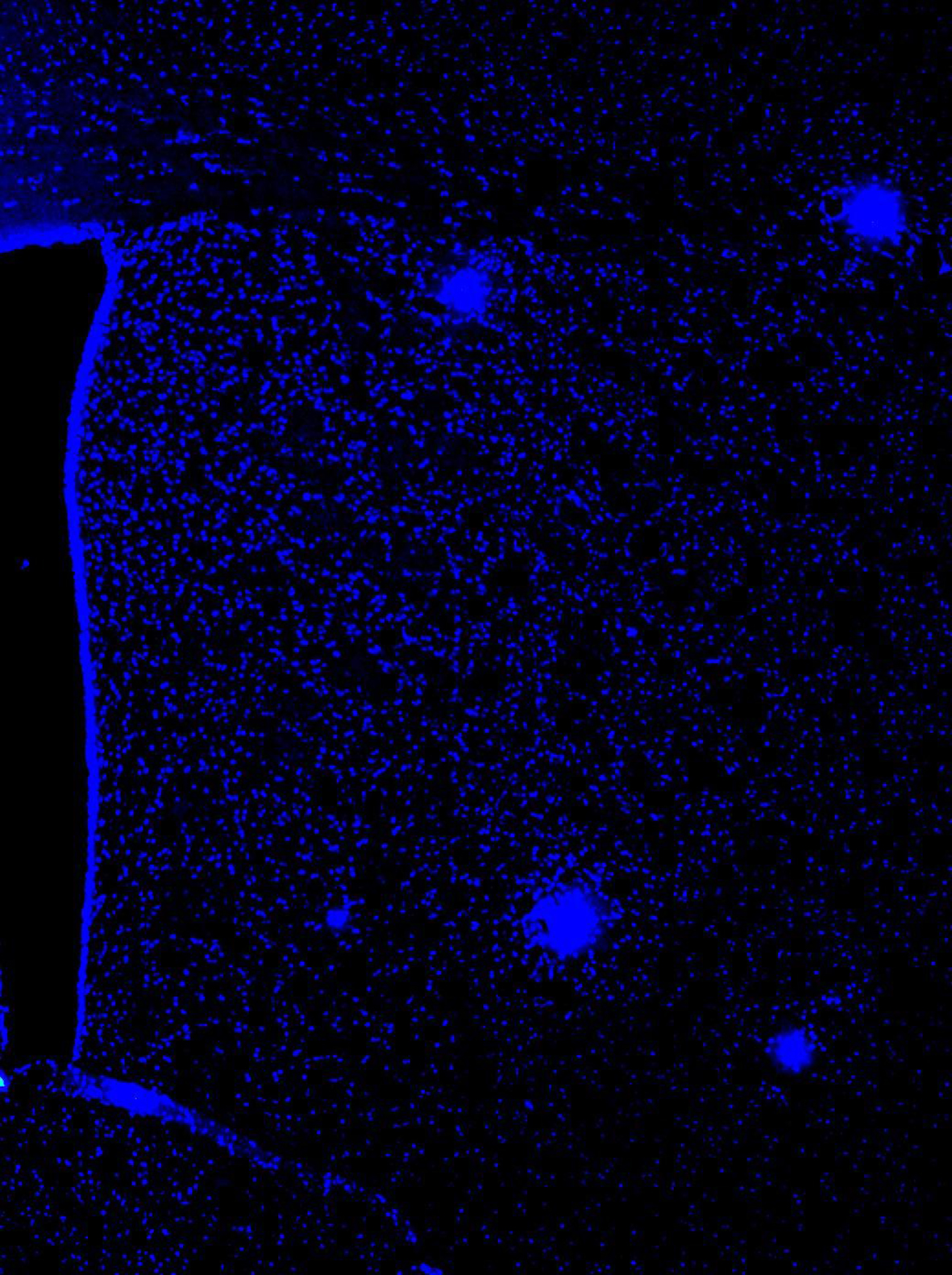

Supplement: S4 File — (ZIP) [file pone.0256488.s005.zip › S4 File/8 Figure S1B 14j.jpg]

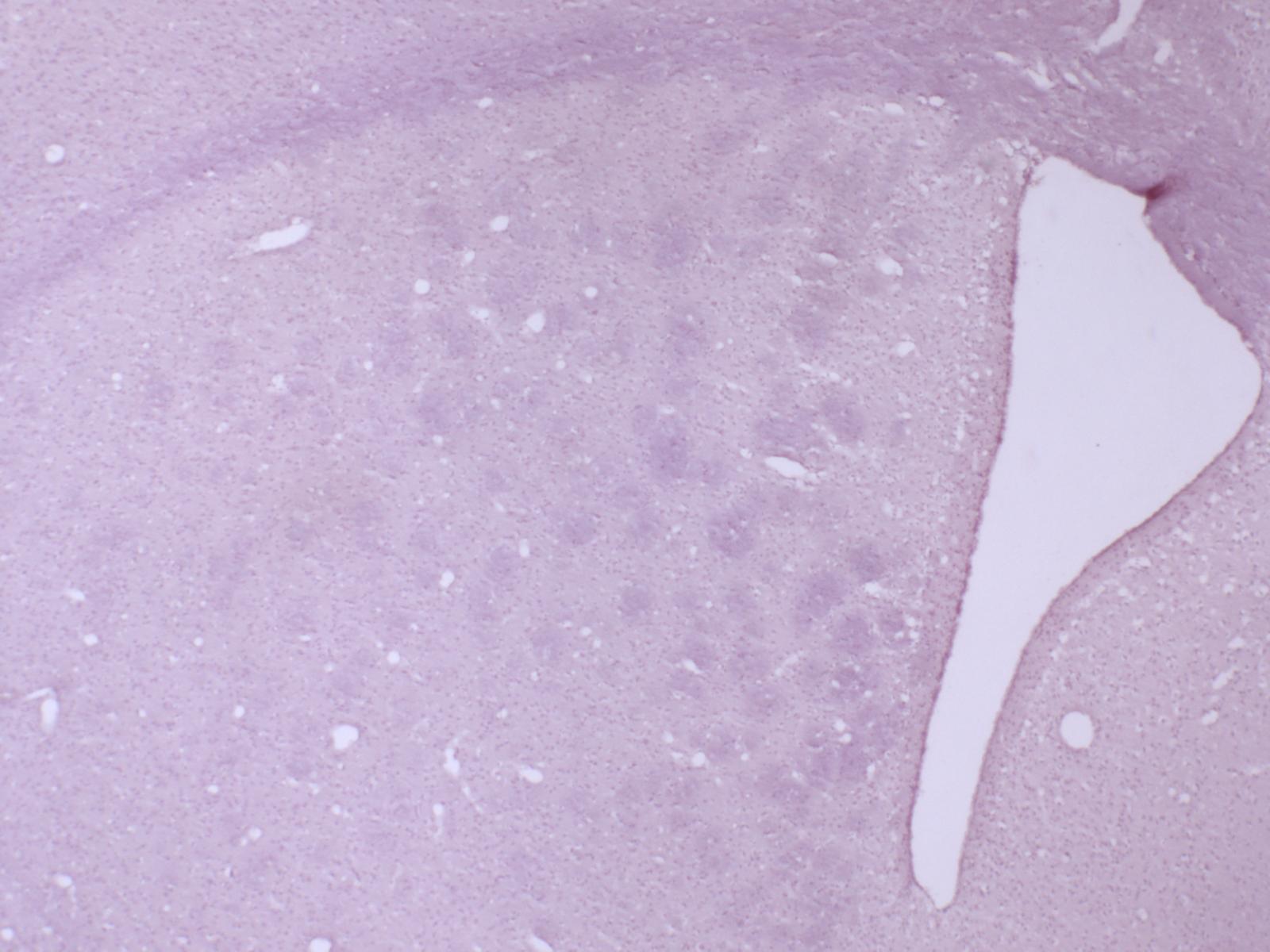

Supplement: S4 File — (ZIP) [file pone.0256488.s005.zip › S4 File/9 Figure S1A 28J PBS NCSC.jpg]

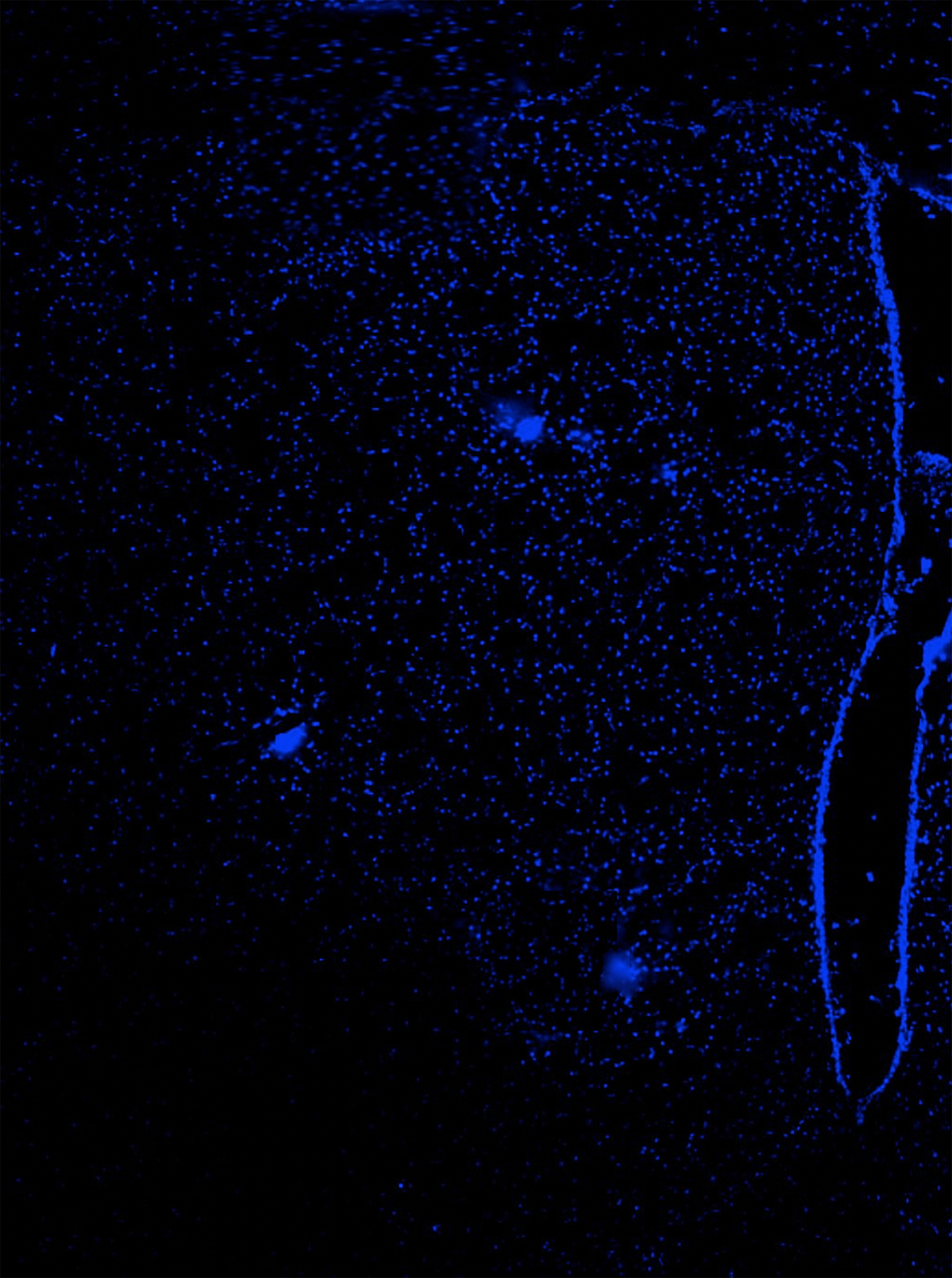

Supplement: S4 File — (ZIP) [file pone.0256488.s005.zip › S4 File/9 Figure S1B.jpg]

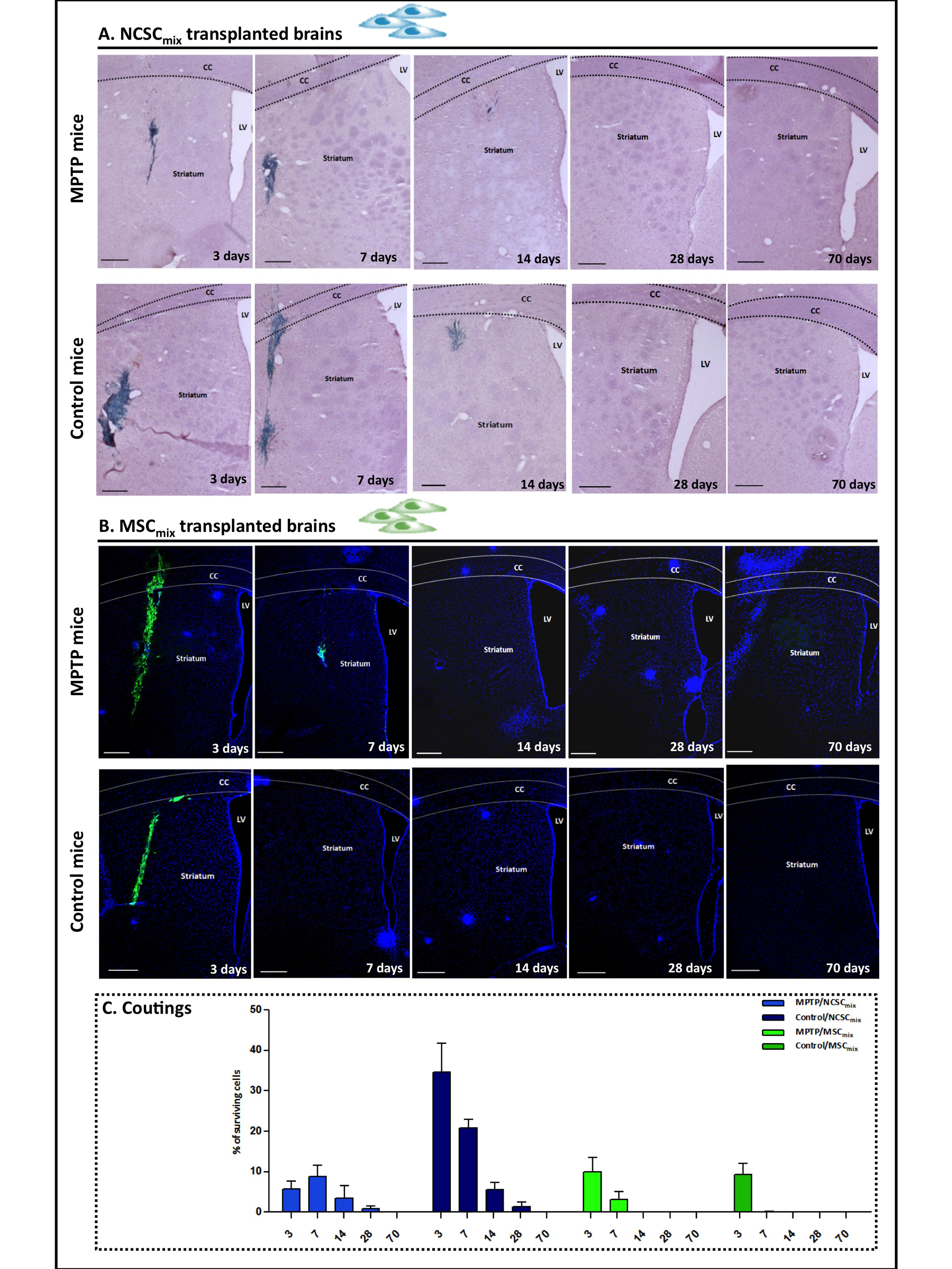

Supplement: S6 File — (TIF) [file pone.0256488.s007.tif]
