# Supplementary material for: Expression of Concern: Adult Bone Marrow Neural Crest Stem Cells and Mesenchymal Stem Cells Are Not Able to Replace Lost Neurons in Acute MPTP-Lesioned Mice
Source: PLoS One. 2021 Oct 28;16(10):e0256488. doi: 10.1371/journal.pone.0256488 (PMC8553060; doi:10.1371/journal.pone.0256488)
Supplement: S5 File — (PDF) [file pone.0256488.s006.pdf]

Adult Bone Marrow Neural Crest Stem Cells and Mesenchymal Stem Cells Are Not Able to Replace Lost Neurons in Acute MPTP-Lesioned Mice

Virginie Neirinckx[1], Alice Marquet[1], Cécile Coste[1], Bernard Rogister[1,2,3]⁹, Sabine Wislet-Gendebien[1]*⁹

1 Groupe Interdisciplinaire de Génoprotéomique Appliquée (GIGA), Unit of Neurosciences, University of Liege, Liège, Belgium, 2 GIGA, Unit of Development, Stem Cells and Regenerative Medicine, University of Liège, Liège, Belgium, 3 Department of Neurology, Centre Hospitalier Universitaire de Liège, Liège, Belgium

Abstract

Adult bone marrow stroma contains multipotent stem cells (BMSC) that are a mixed population of mesenchymal and neural-crest derived stem cells. Both cells are endowed with *in vitro* multi-lineage differentiation abilities, then constituting an attractive and easy-available source of material for cell therapy in neurological disorders. Whereas the *in vivo* integration and differentiation of BMSC in neurons into the central nervous system is currently matter of debate, we report here that once injected into the striatum of 1-methyl-4-phenyl-1,2,3,6-tetrahydropyridine (MPTP)-treated mice, pure populations of either bone marrow neural crest stem cells (NCSC) or mesenchymal stem cells (MSC) survived only transiently into the lesioned brain. Moreover, they do not migrate through the brain tissue, neither modify their initial phenotype, while no recovery of the dopaminergic system integrity was observed. Consequently, we tend to conclude that MSC/NCSC are not able to replace lost neurons in acute MPTP-lesioned dopaminergic system through a suitable integration and/or differentiation process. Altogether with recent data, it appears that neuroprotective, neurotrophic and anti-inflammatory features characterizing BMSC are of greater interest as regards CNS lesions management.

Citation: Neirinckx V, Marquet A, Coste C, Rogister B, Wislet-Gendebien S (2013) Adult Bone Marrow Neural Crest Stem Cells and Mesenchymal Stem Cells Are Not Able to Replace Lost Neurons in Acute MPTP-Lesioned Mice. PLoS ONE 8(5): e64723. doi:10.1371/journal.pone.0064723

Editor: Alexandre Hiroaki Kihara, Universidade Federal do ABC, Brazil

Received March 20, 2013; **Accepted** April 18, 2013; **Published** May 31, 2013

Copyright: © 2013 Neirinckx et al. This is an open-access article distributed under the terms of the Creative Commons Attribution License, which permits unrestricted use, distribution, and reproduction in any medium, provided the original author and source are credited.

Funding: This work was supported by a grant from the Fonds National de la Recherche Scientifique (FNRS) of Belgium, by the Belgian League against Multiple Sclerosis associated with the Leon Frédericq Foundation and by the Fonds Spéciaux à la Recherche of the University of Liège. The funders had no role in study design, data collection and analysis, decision to publish, or preparation of the manuscript.

Competing Interests: The authors have declared that no competing interests exist.

* E-mail: s.wislet@ulg.ac.be

⁹ These authors contributed equally to this work.

Introduction

The treatment of neurological disorders represents a critical issue in clinical research, since no complete functional recovery can be achieved with current therapeutic means, despite symptomatic improvements. Indeed, whereas restricted brain areas still house cells competent to generate newborn neurons in adulthood [1,2], this limited neurogenesis does not seem to be sufficient to enable neuronal regeneration in cases of lesions of the central nervous system. Therefore, other sources of neural cells have to be considered in a cell therapy objective. Stem cells are characterized as cells endowed with continuous self-renewal ability and pluri- or multipotentiality [3], and could consequently give rise to a wide panel of cell types, including neural cells. Indeed, while neurons have already been successfully generated from embryonic stem cells (ES) [4,5] or induced pluripotent stem cells (iPS) [6,7], the use of adult somatic stem cells definitely remains of significant interest regarding technical, ethical and immunological issues concerning cell transplantation for brain-related diseases. In this regard, bone marrow stromal cells (BMSC) represent an important source of easily-accessible multipotent cells to use in a cell therapy purpose [8].

Numerous studies already described cell therapy experiments using BMSC and explored their neuronal plasticity *in vivo* [9–11]. However, result discrepancies appeared in those studies, which could mainly reside in the lack of exact phenotypic characterization of BMSC, due to the absence of specific membrane markers and non-standardized culture methods. Consequently, several groups described BMSC with major different phenotypes: Verfaillie's group described a rare population of cells in human BM stroma as mesodermal adult progenitor cells [12,13]; D'Ippolito and collaborators cultured cells in low oxygen tension and characterized marrow isolated adult multilineage inducible cells [14,15]; whereas a lot of other groups kept the mesenchymal stem cell (MSC) concept as defined by Pittenger et al. [16]. In addition to the phenotypic differences of BMSC which are inherent to culture settings, it has been demonstrated that BMSC are constituted by a mixed population of cells arising from different embryonic lineages. Indeed, although adult BMSC were commonly considered to be of mesodermal origin [17], several studies have conclusively shown that some adult BMSC derive from the neural crest [8,18–21].

The main objective of this study was consequently to specifically analyze the capacity of *in vivo* differentiation of the two distinct populations of BMSC: mesenchymal stem cells (MSC) and neural crest stem cells (NCSC), both isolated from adult bone marrow and recently characterized by Wislet-Gendebien et al. [21,22], when injected into lesioned brain. Indeed, we know that bone marrow NCSC are present in low proportion inside primary

BMSC cultures compared to the MSC [22]. Consequently, a graft of pure bone marrow NCSC could lead to different results than observed with BMSC and could be able to restore brain lesions through a neural differentiation process in a larger extent, due to their neural crest developmental origin. We therefore grafted NCSC and MSC pure populations into the brain of mice characterized by dopaminergic nigrostriatal pathway lesions (mimicking the dopaminergic cell loss in advanced stages of Parkinson's disease) induced by previous 1-methyl-4-phenyl-2,3,5-tetrahydropyridine hydrochloride (MPTP-HCl) injections. We then investigated neural differentiation events and downstream effects on the nigrostriatal pathway integrity, in order to evaluate potential of NCSC and MSC therapeutic abilities once inside the lesioned brain.

Materials and Methods

Animal Care

Wnt1-Cre/R26R-LacZ double transgenic mice were used to isolate NCSC and MSC clones from adult bone marrow stromal cells cultures [22]. 12 to 16-week-old wild type C57BL/6J mice (The Jackson Laboratory, Bar Harbor, ME, USA) were used as recipient mice for graft experiments. Animals were bred at the University of Liège Central Animal facility and experiments were performed in accordance with the rules set by the local animal ethics committee (ethical permit 1038) as well as the Swiss Academy of Medical Sciences.

MPTP Administration

Five days before the cell graft experiment, 1-methyl-4-phenyl-1,2,3,6-tetrahydropyridine hydrochloride (MPTP-HCl) (Sigma-Aldrich, St-Louis, MO, USA) is suspended in sterile PBS solution at a concentration of 5 mg/mL. Mice received four intraperitoneal injections of 20 mg/kg MPTP-HCl, at two hours interval (100 to 120 µL for 25 to 30 g-weight mice), as already described in [23,24], triggering acute bilateral dopaminergic neurons cell death.

Cell Culture Procedures and Clonal Selection

Bone marrow cells from adult (8–10 week-old) *Wnt1-Cre/R26R*-LacZ mice were obtained from femoral and tibial bones by aspiration and were resuspended in MesenCult Medium (MesenCult, Stem Cells Technologies, Grenoble, France). After 24 hours, non-adherent cells were removed. After reaching confluence, BMSC were resuspended using 0,05% trypsin-EDTA (Life Technologies, Carlsbald, CA, USA) and then sub-cultured (750,000 cells/25 cm^2) at 37°C, in a 95% O_2/5% CO_2 atmosphere. For clonal selection, passage 5 BMSC were seeded in a 96 well plate (Thermo Fisher Scientific, Langenselbold, Germany) at a mean dilution of 0.7 cell/well, in MesenCult Medium. At confluence, cells were dissociated with 0,05% trypsin-EDTA and subcultured in the same conditions.

Genomic Validation of Wnt1-CRE/R26R-LacZ Recombination

DNA was isolated from $NCSC_{mix}$ and MSC_{mix} using the QIAamp DNA Mini Kit extraction protocol (Qiagen, Germantown, MD, USA). Briefly, cells were incubated at 56°C with proteinase K in a lysis buffer for 10 min, then genomic DNA was purified through several silica-membrane-based steps (see manufacturer's instructions). DNA amount was then calculated via a NanoDrop spectrophotometer (Thermo Scientific). Afterwards, DNA sequences of interest were amplified by polymerase chain reaction by mixing 500 ng of genomic DNA with Taq Polymerase (Promega) and specific primers (PGK-Neo: For-ATGGATTG-CACGCAGGTTCTCC; Rev-CAGAAGAACTCGTCAA-GAAGGC and actin: For-ATCTTGATCTTCATGGTGC-TAGG; Rev-TGTTACCAACTGGGACGACATGG) in a T3000 thermocycler (Biometra, Göttingen, Germany).

Cell Preparation and Transplantation

Just before the transplantation, two cell solutions containing respectively 5 NCSC clones and 5 MSC clones in equal numbers were prepared. Whereas $NCSC_{mix}$ were already traceable thanks to their β-galactosidase activity, we needed to label MSC_{mix} with Cell Tracker Green (CTG) (Life Technologies) to allow their traceability *in vivo*. Mice were anesthetized with 100 mg/kg of a solution containing equivalent volumes of xylazine (Rompun, Bayer, Belgium) and ketamine (Ketalar, Pfijzer, Belgium). They were then placed into a stereotaxic frame (Benchmark, MyNeuroLab.com) and received one injection of 5×10^4 cells suspended in 2 µL PBS (Life Technologies) in the right striatum (0,5 mm anterior, 2 mm lateral and 3 mm ventral, with respect to bregma). The intracerebral injection was performed using a Hamilton's 5 µl syringe, coupled with a 26-gauge needle. The needle was left in place for few minutes before being retracted, to avoid reflux along the injection track. After the surgery, mice were placed under a warm lamp until their complete awakening.

Brain Processing

At different delays following cell transplantation, animals were anesthetized with pentobarbital and sacrificed by intracardiac perfusion of ice-cold PBS, followed by paraformaldehyde (PFA) 4% (in PBS 0,1 M), at. Skulls were dissected and brains were immediately removed, post-fixed for 2 hours at 4°C in the same fixative then immersed overnight in a solution of sucrose 20% (in PBS 0,1 M). They were frozen by slow immersion in isopentane cooled on dry-ice. Coronal 14 µm-sections were cut using a cryostat, mounted on positively charged slides, and stored in −20°C for further experiments (30 slides covering the entirety of the striatum and 10 slides covering the entirety of the midbrain).

DNA Extraction from Striatal Slices and PCR Validation of Survival Rate Evaluation

DNA was extracted from 14 µm-striatal slices (after −20°C storage) using the PrepFiler Forensic DNA Extraction Kit (Life Technologies). Right striatum was microdissected on about 10 slices and then scraped into a 1,5 mL microcentrifuge tube. After lysis with proteinase K and heat/shake treatment, genomic DNA was bound to PrepFiler Magnetic Particles and then eluted in order to amplify sequences of interest by PCR (See *Genomic validation of Wnt1-CRE/R26R-LacZ recombination* section).

Immunostainings

Briefly, 14-µm brains slices (or cells on coverslips) were incubated for 1 hour with 10% normal donkey serum in PBS 0,1 M (supplemented with 0,3% Triton X-100 for intracellular antigens). For specific immunofluorescent staining, anti-nestin (1:300, NB100-1604; Novus Biologicals, Littleton, CO, USA), anti-βIII-tubulin (1:1000, MMS-435P; Covance, Princeton, NJ, USA), anti-GFAP (1:1000, Z0334; Dako, Glostrup, Denmark), anti-TH (1:250, ab112; Abcam, Cambridge, UK), anti-Sca-1 (1:100, ab25195; Abcam), anti-Fzd-4 (1:100, MAB194; R&D System, Minneapolis, MN, USA) and anti-CD24 (1:200, ab64064; Abcam) were diluted in PBS 0,1 M overnight at 4°C. After three PBS washes, brains sections were incubated with FITC or Rhodamine Red X-conjugated secondary antibodies (1:500;

Jackson Immunoresearch Laboratories, West Grove, PA, USA) for 1 hour at room temperature. Nuclei were then counterstained with Hoescht 33342 (Molecular Probes, Life Technologies) and finally mounted in Q Path Safemount (Labonord, Templemars, France).

The same steps and panel of primary antibodies were used for immunochemistry, but stainings were acquired using peroxydase-coupled secondary antibodies (1:500, Dako) and diaminobenzidine revelation.

Image acquisition and analysis were performed using a Zeiss AxioImager Z1 epifluorescent microscope (Zeiss, Zaventem, Belgique) coupled with FluoView software (Olympus, Artselaar, Belgique), and Olympus AX-70 microscope (Olympus) coupled with AnalySIS software (Olympus). The digitized images were adjusted for brightness and contrast, color-coded, and merged, when appropriate, using the NIH program ImageJ (Wayne Rasband, National Institute of Mental Health, Bethesda, MD, USA).

Other Stainings

X-gal staining. Brains sections were incubated for 2 hours in PBS supplemented with Tris (pH 7,4) 20 mM, $MgCl_2$ 2 mM, 0.02% NP-40, 0.01% Na-deoxycholate, $K_3Fe(CN)_6$ 5 mM (Sigma-Aldrich), $K_4Fe(CN)_6$ 5 mM (Sigma-Aldrich) and 1-methyl-3-indolyl-beta-Dgalactopyranoside 1 mg/ml (Sigma-Aldrich) suspended in DMSO. The reaction was stopped by PBS washes.

Carazzi hematoxylin coloration. Dry brain sections were placed in denatured ethanol and slightly heated for approximately 4 minutes, then were washed three times in milliQ water, before an incubation of 10 minutes in Carazzi hematoxylin. After three washes in water, sections were finally mounted with Q Path Safemount (Labonord).

Quantification of Cell Survival and Number of Neurons in the SN and VTA

Cell survival was quantified as followed: To evaluate the number of $NCSC_{mix}$ in the brains at each time point post-transplantation, X-gal staining was performed on 4 slides containing striatal slices (4 slides covering the 30 slides : e.g. slide 1–10–20 - 25). Nuclei were counterstained with Hoescht, and after superposition of X-gal/Hoescht staining, X-gal positive nuclei were counted in each striatal sections on the slides. We then normalized the number of X-gal positive nuclei for all the 30 sections covering the entirety of striatum, and expressed this number in % regarding the 5×10^4 cells that were initially injected. To evaluate the number of MSC_{mix} in the brains at each time point post-transplantation, co-localization of Cell Tracker Green and Hoescht was used and the same countings and normalizations were performed.

To evaluate the nigrostriatal pathway integrity, SNpc and VTA neurons were counted as previously described in the followed MPTP-administration protocol [23]. Our neuronal counts were expressed as mean number of neurons per representative mesencephalic plane. For each mouse, sections covering the entire rostrocaudal axis of the mesencephalon were analyzed. The mean number of neurons for each representative mesencephalic plane was obtained by averaging the number of neurons counted from both right and left SNpc or VTA areas, respectively.

Statistical Analysis

Data were analyzed statistically using Statistica 10 program (StatSoft, Tulsa, OK, USA). Results are reported as mean ± standard error of mean, with the n described as the number of mice in each group. Level of statistical significance was set at $p<0.05$.

Results

Clonal Selection of NCSC and MSC from Adult Bone Marrow

Since we previously demonstrated that neural crest stem cells were mainly composed of nestin-positive cells [22] and that the number of nestin-positive cells increased with the number of passages [25], we decided to perform clonal selection of NCSC and MSC starting from passage 5, which should theoretically give us equal chances to isolate NCSC or MSC. Single cell BMSC were placed in a 96-wells plate in MesenCult medium allowing 1.2% of cells to proliferate. NCSC clones or MSC clones were then pooled together creating two distinct and pure population: $NCSC_{mix}$ and $MSC_{mix.}$ We first verified that cells in $NCSC_{mix}$ were effectively derived from initial embryonic neural crest cells that underwent Cre-Lox recombination, conversely to MSC clones that were not neural crest-derived (Fig. 1 A–B). $NCSC_{mix}$ (β-galactosidase-positive cells; Fig 1.C) and MSC_{mix} (β-galactosidase-negative cells; Fig 1.D) were then characterized *in vitro*. As previously described [22], $NCSC_{mix}$ were Sox10-positive (Fig 1.D), nestin-positive (Fig. 1.E) and $p75^{NTR}$-positive (Fig. 1.F) while MSC_{mix} were Sox10-negative (Fig. 1.L), less than 15% were nestin-positive (Fig 1.M) and weakly $p75^{NTR}$-positive (Fig. 1.N). MSC_{mix} also expressed Fzd-4 (Fig. 1.Q), Sca-1 (Fig. 1.O) and CD24 (Fig. 1.P) while $NCSC_{mix}$ were only positives for Fzd-4 (Fig. 1.I). Additionally, βIII-tubulin positive cells were observed in $NCSC_{mix}$ when cultivated in Mesencult medium (without any differentiation protocol, Fig. 1.J). At the opposite, no βIII-tubulin positive cells were observed among MSC_{mix} (Fig. 1.R). However, $NCSC_{mix}$ and MSC_{mix} were both negative for more mature or specific neuronal markers like MAP2ab or TH and for GFAP (data not shown). We then decided to characterize $NCSC_{mix}$ and MSC_{mix} differentiation and therapeutic abilities *in vivo* using the MPTP mouse model.

MPTP Mouse Model Validation

In order to verify MPTP-injection impact on nigrostriatal system (classically affected in Parkinson's disease) and validate our experimental model, we quantified the number of dopaminergic neurons by immunostaining of tyrosine hydroxylase (TH) (limiting enzyme in dopamine synthesis). As observed on Fig. 2.A, a drastic decrease in TH-positive fibers in the entire striatum was observed in MPTP-treated animals compared to control (>95%). At the midbrain level, TH-positive cell bodies in the Substantia Nigra *pars compacta* (SNpc) of MPTP-treated mice were quantified (45,93±5,86 cells per representative mesencephalic plane, n = 5) and revealed a significant decrease (60%) of the number of cells compared to the control condition (129,40±10,50 TH-positive neurons per representative mesencephalic plane; n = 5; One-way ANOVA, $p<0,001$; Fig. 2.A–B). Ventral tegmental area (VTA) provides an internal control zone: VTA dopaminergic neurons were affected by MPTP in a lesser extent than SNpc neurons [26], and the difference between controls and MPTP-treated animals was not significant, attesting of MPTP specificity for SNpc neurons (One-way ANOVA, $p>0,05$). Those results were consistent with already published studies [23] (Fig. 2.A–B).

$NCSC_{mix}$ and MSC_{mix} Survival Rate

$NCSC_{mix}$ and MSC_{mix} were injected in mice striatum 5 days post-MPTP injection. Surviving cells were quantified after 3, 7, 14, 28 and 70 days following cell graft, on all brain sections by counting nuclei co-localizing with X-gal staining or Cell Tracker

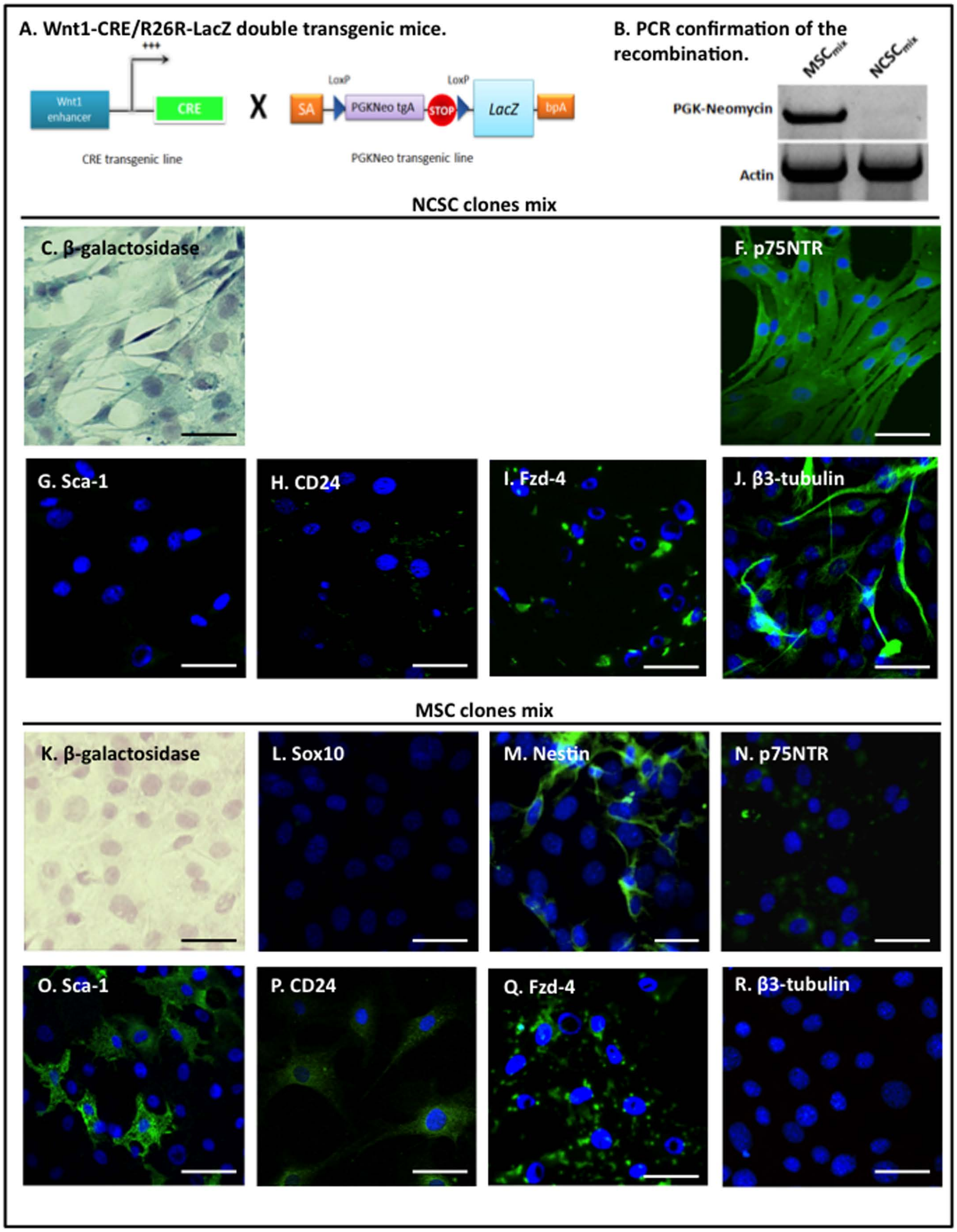

Figure 1. In vitro characterization of $NCSC_{mix}$ and $MSC_{mix,}$ isolated from adult Wnt1-Cre/R26R-LacZ mouse bone marrow. BMSC were harvested from double-transgenic Wnt1-CRE/R26R-LacZ mouse bone marrow (A). Amplification of the PGK-Neo cassette confirmed that $NCSC_{mix}$

underwent recombination, conversely to MSC_{mix} (B). $NCSC_{mix}$ were β-galactosidase positive (*blue*, C), whereas MSC_{mix} were not (*blue*, K) (Hematoxylin–stained nuclei). $NCSC_{mix}$ expressed neural crest-associated proteins Sox10 (*green*, D), Nestin (*green*, E) and p75NTR (*green*, F). MSC_{mix} were Sox10-negative (*green*, L), weakly p75NTR-positive (*green*, N), and only a small proportion (<15%) of cells expressed nestin (*green*, M). MSC_{mix} also expressed Sca-1 (*green*, O) and CD24 (*green*, P) while $NCSC_{mix}$ did not (*green*, G-H), and both types of cells were positive for Fzd-4 (*green*, I-Q). In $NCSC_{mix}$, some β-tubulin-expressing cells were detected (in MesenCult medium) (*green*, J), but no cell in the MSC_{mix} was β-tubulin-positive in those conditions (*green*, R) (DAPI-stained nuclei). (Scale bars = 30 μm).
doi:10.1371/journal.pone.0064723.g001

Green (CTG) (for respectively grafted $NCSC_{mix}$ and MSC_{mix}). We first observed that transplanted cells (both $NCSC_{mix}$ and MSC_{mix}) staid tightly confined to the engraftment site, without any evident signs of migration through the brain tissue: no grafted cells were recovered into the lesioned SNpc or anywhere else inside the brain. As observed on Fig. 3.B, around 10% of $NCSC_{mix}$ survived up to 7 days post-graft and 3% up to 14 days. After that delay, less than 1% of $NCSC_{mix}$ were detected. Similar results were observed for MSC_{mix} as the mean survival rate of grafted cells was evaluated at 10% after 3 days. The survival rate at 7 days post-graft was decreased to 3%, and no grafted MSC were detected at 14 days post-graft. Control mice (injected with saline instead of MPTP) were also grafted with the same number of $NCSC_{mix}$ or MSC_{mix} and we observed that the cells also disappeared within a 28 days timeframe (Fig. S1). As the stability of CTG fluorescence with time could be questioned, we confirmed by PCR that the cells were totally gone from the brain starting from 14 days after transplantation. In that purpose, we microdissected transplanted striatum slices and we showed that no PGK-Neo signal was observed starting from 14 days post transplantation (in saturating conditions) (Fig. 3.B).

Phenotypic Characterization of Grafted MSC_{mix}/$NCSC_{mix}$

At each time point post-transplantation, grafted $NCSC_{mix}$ and MSC_{mix} were tested for nestin, glial fibrillary acidic protein (GFAP), βIII-tubulin, and tyrosine hydroxylase (TH) immunoreactivity. Similar results than the one observed *in vitro* were recovered as transplanted $NCSC_{mix}$ expressed nestin (Fig. 4.E) while MSC_{mix} did not (Fig. 4.I) [22], and both types of cells were GFAP-negative (Fig. 4.F,J). Regarding βIII-tubulin expression, specific co-localization was more difficult to appreciate since the environing brain tissue was entirely immunoreactive. However, only few positive cells were observed for $NCSC_{mix}$ (Fig 4.G), and no evidence was noticed for MSC_{mix} (Fig. 4.K). Finally, no grafted cell (neither $NCSC_{mix}$ nor MSC_{mix}) differentiated into functional dopaminergic cells, as attested by their negativity for TH marker (Fig. 4.H,L and Fig. S2). According to those results, it appeared that the *in vivo* environment (MPTP-lesioned striatum) did not induce any modification in the phenotype of transplanted $NCSC_{mix}$ and MSC_{mix}.

Effects of Cell Graft on MPTP-induced Lesions

We evaluated the integrity of nigro-striatal pathway thanks to the immunolabelling of tyrosine hydroxylase (TH). As already mentioned, the number of TH-positive neurons in the SNpc of

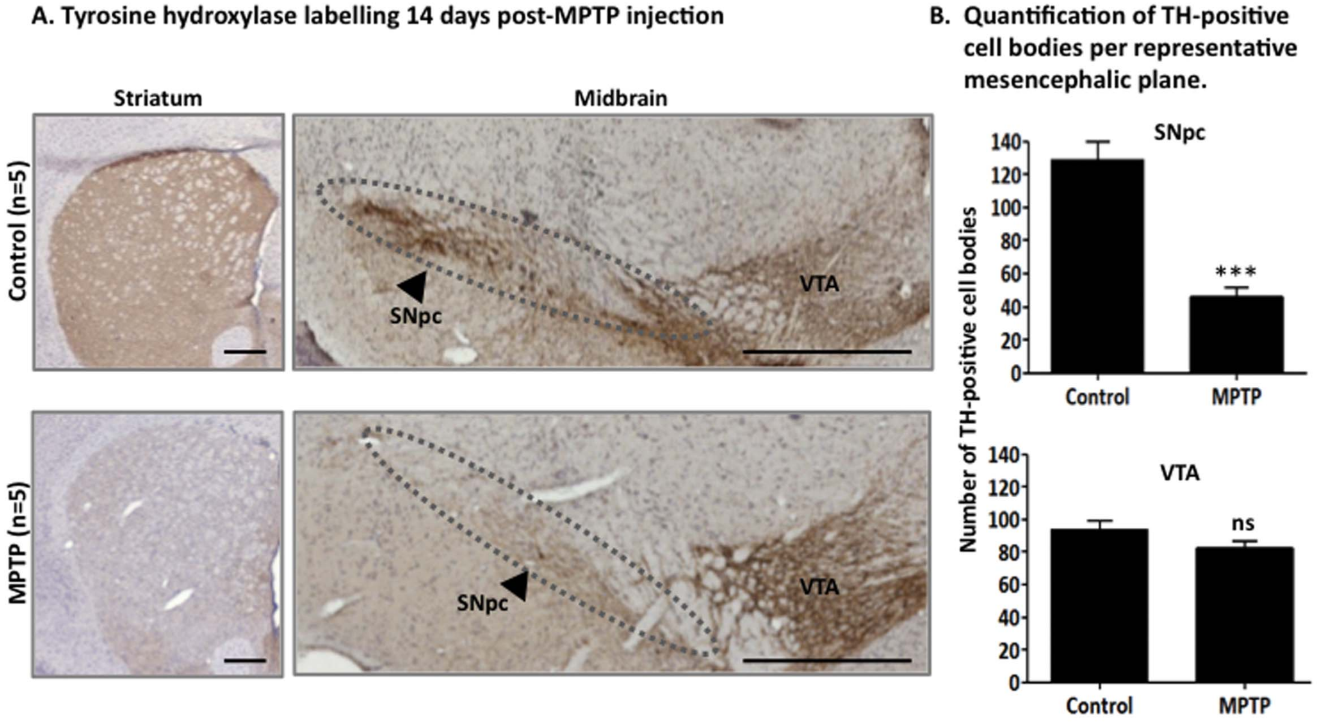

Figure 2. Validation of the MPTP mouse model of dopaminergic cell degeneration. A. Tyrosine hydroxylase (TH) immunostaining (*brown*, hematoxylin-stained nuclei) of dopaminergic fibers in the striatum and neurons in the midbrain of control and acute MPTP-treated mice. **B.** Number of TH-positive cell bodies per representative mesencephalic plane (Mean ± SEM). The number of dopaminergic neurons in the SNpc of MPTP-treated mice is significantly decreased compared to control ($p<0{,}001$, One-way ANOVA), while the VTA dopaminergic neurons are not strongly affected ($p>0{,}05$, One-way ANOVA). (Scale bars = 500 μm).
doi:10.1371/journal.pone.0064723.g002

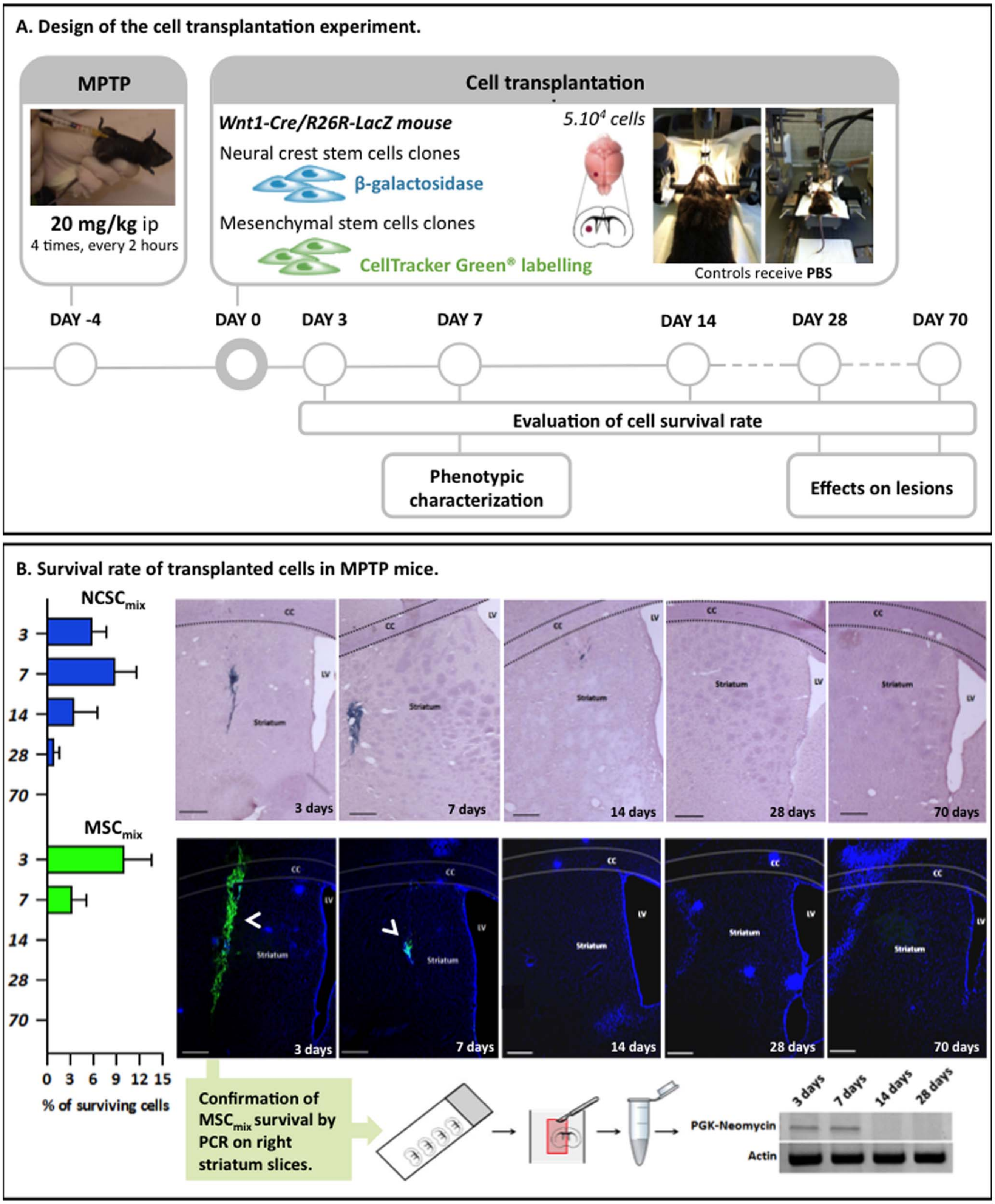

Figure 3. Intrastriatal transplantation of MSC_{mix}/$NCSC_{mix}$ in MPTP mice and survival rate of grafted cells at 3, 7, 14, 28 and 70 days after transplantation. A. Experimental design of the lesion and transplantation experiment. Adult C57Bl/6J male mice were injected with MPTP following the "classical" acute regimen. Five days after MPTP treatment MSC_{mix}/$NCSC_{mix}$ were injected into the right striatum of mice (MSC_{mix} were first stained with Cell Tracker Green in order to be detected *in vivo*). Survival rate evaluation and phenotypic characterization were performed at different delays post-graft. **B.** The number of surviving $NCSC_{mix}$ in the right striatum (*blue* X-gal staining and *purple* Hematoxylin-stained nuclei) can reach 15% in the first week after transplantation, then the cells begin to disappear and after 4 weeks, we only observe a mean survival rate of 1%. Results are expressed in %, according to the 5×10^4 injected cells (Mean $\pm$ SEM). MSC_{mix} (*green* CTG staining, *blue* DAPI-stained nuclei) seem to

disappear more rapidly than $NCSC_{mix}$, since no cells were observed starting from 14 days. (n≥3 for each group, at each delay post-transplantation). As CTG relevancy might be questioned, the temporary survival of cells was confirmed by PCR amplification of the PGK-Neomycin cassette in grafted MSC_{mix}. (CC = Corpus callosum; LV = Lateral ventricle; Scale bars = 500 μm).
doi:10.1371/journal.pone.0064723.g003

MPTP-treated animals was decreased for more than 50%, compared to control animals, and the dopaminergic fibers in the striatum nearly completely disappeared (Fig. 5.A). After 28 and even 70 days following the cell transplantation, no improvement in striatal TH staining was observed in MPTP-injected mice in both conditions ($NCSC_{mix}$-grafted group, Fig. 5.B and MSC_{mix}-grafted group, Fig. 5. C). Similarly, we did not see any significant modification in the number of host TH-positive cell bodies into the SNpc of MPTP mice that received cell grafts (Fig. 5.D) (Kruskal-Wallis ANOVA, p>0,05).

Discussion

Adult bone marrow stromal cells (BMSC) are of significant interest in cell therapy, regarding their accessible location, low immunogenicity and multipotentiality. Previous studies already described their neural differentiation abilities [27,28], then confirming their potential use in the treatment of neurological diseases. Moreover, the presence of neural crest derived cells into the adult bone marrow stroma [21,22] raised new hopes to obtain functional neurons from autologous adult stem cells [8]. Recently, a clinical trial described unilateral transplantation of autologous whole BMSC population into the subventricular zone (SVZ) of PD patients, and reported reserved clinical improvement with no adverse effects, such as tumor formation [29,30]. Whereas those results were based on clinical observations and Unified Parkinson's disease Rate Scale scores, the mechanisms underlying the reported improvements are completely unknown.

To the light of those observations, the main objective of this study was to determine if bone marrow neural crest stem cells (NCSC) were responsible for the positive impact of bone marrow stromal cells in several PD models rather than mesenchymal stem cells (MSC). In this study, we addressed the aspect of neural differentiation abilities of pure NCSC or MSC populations by directly injecting those cells into the lesioned brain. In this purpose, the model we selected was an acute lesion of dopaminergic system, induced by 1-methyl-4-phenyl-1,2,3,6-tetrahydropyridine (MPTP) injection [23,31,32]. Consequently, cells were grafted five days after MPTP injection in order to pass up ongoing cell death and acute inflammatory events [33–35], with the aim of

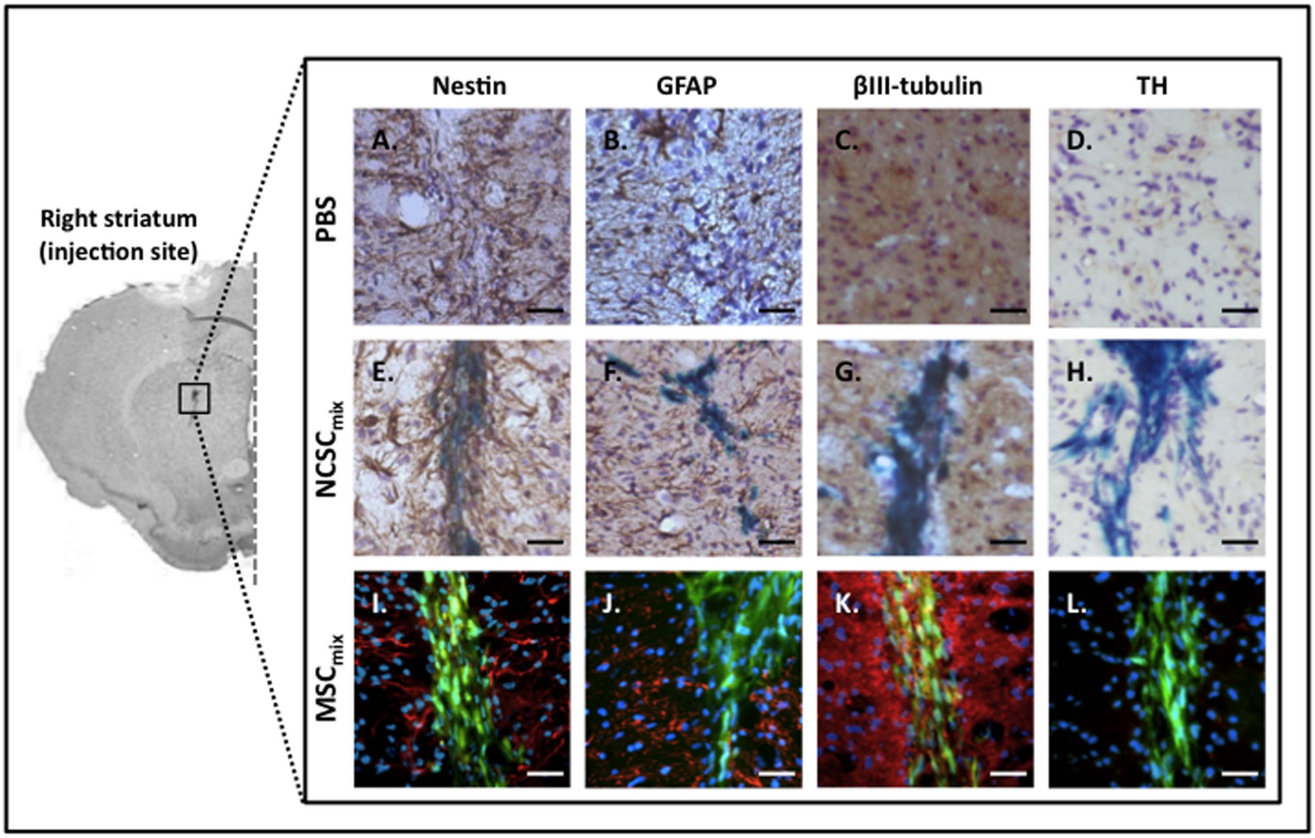

Figure 4. In vivo characterization of brain-injected $NCSC_{mix}$ and MSC_{mix}. All cells were implanted in the correct brain site, in each mouse that was included in the study. Transplanted $NCSC_{mix}$ were detected by X-gal staining (*blue*) and MSC_{mix} were identified thanks to CTG staining (green). Grafted cells conserved their *in vitro* phenotype, whatever the delay post-transplantation : $NCSC_{mix}$ were nestin-positive (*brown*, A) whereas MSC_{mix} were not (*red*, I). No cells did differentiate into GFAP-positive cells ($NCSC_{mix}$, *brown*, F; MSC_{mix}, *red*, J). Only few positive cells in $NCSC_{mix}$ were βIII-tubulin-positive (*brown*, G), and no specific positivity was noticed for MSC_{mix} (*red*, K). Finally, grafted cells were negative for TH ($NCSC_{mix}$, *brown*, F; MSC_{mix}, *red*, J). Nestin and GFAP staining (*brown* or *red*) are detected around the transplanted cells and are linked with injection-induced inflammation (n≥3 for each group, at each delay post-transplantation). Scale bars = 25 μm.
doi:10.1371/journal.pone.0064723.g004

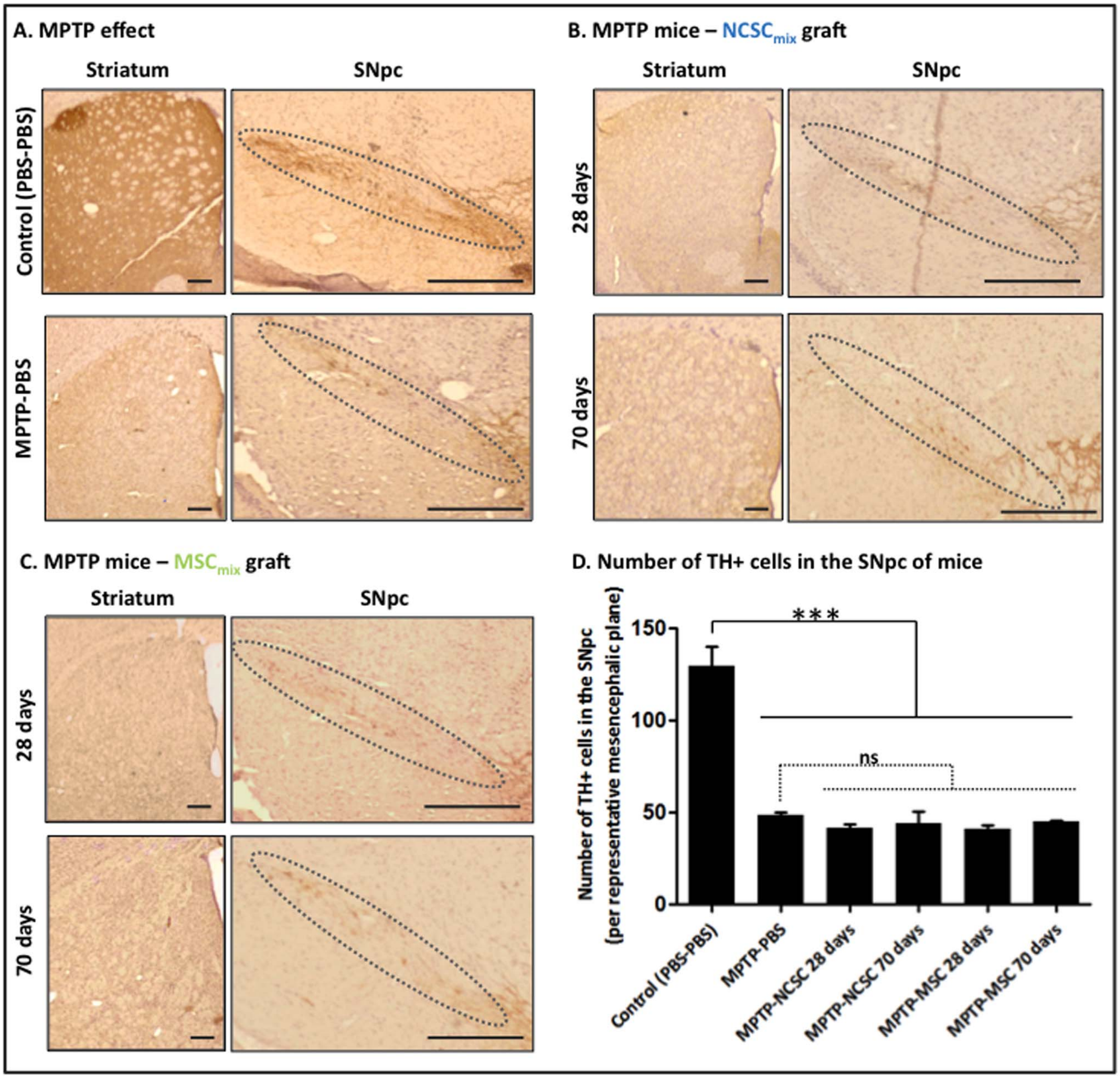

Figure 5. Evaluation of $NCSC_{mix}$ and MSC_{mix} graft consequences on the number of host TH-positive neurons in MPTP-induced dopaminergic lesions. **A.** Effect of MPTP on the integrity of dopaminergic nigro-striatal pathway (at 28 days post-MPTP treatment). **B.** $NCSC_{mix}$ graft in MPTP-treated mice. No increase in TH-positive (*brown*) striatal dopaminergic fibers and TH-positive cell bodies in the SNpc is observed, at 28 days as well as 70 days post- $NCSC_{mix}$ transplantation. **C.** The same observations were carried out after MSC_{mix} transplantation. **D.** Number of TH-positive cell bodies in the SNpc of MPTP-treated mice that were transplanted with $NCSC_{mix}/MSC_{mix}$ ($p>0{,}05$; Kruskall-Wallis ANOVA). Scale bars = 250 μm.
doi:10.1371/journal.pone.0064723.g005

confirming if a potential enhancement was properly due to phenotypic plasticity and neural differentiation of injected cells. In those conditions, neither NCSC nor MSC survived for more than 28 days into the lesioned brain, neither underwent phenotypic modifications compared to their *in vitro* state before graft. Therefore, it wasn't surprising not to observe any enhancement in nigro-striatal pathway integrity, suggesting that NCSC and MSC were not able to successfully differentiate into neural cells and to integrate and connect with host neurons in acute MPTP-treated mice.

Discrepancies of our observations with previous studies [36] could be justified regarding the chronic MPTP-injection protocol that leads to completely different degeneration kinetics [37]. Indeed, acute-MPTP mouse model do not reflect a real phenomenon of progressive degeneration, we might miss several events that could prompt grafted cells to adapt their phenotype. On the other hand, grafting stem cells in chronic-MPTP mice would be trickier to characterize, as both neuroprotective and neurorestorative events could simultaneously occur.

Nonetheless, our results reinforce the current controversy on BMSC neural differentiation ability. Indeed, whereas loads of

papers describe *in vitro* specific neural proteins expression in BMSC after various neural induction protocols [38–41], few data provide convincing evidence for a neuron-specific electrophysiological signature of the differentiated cells, namely the elicitation of action potentials [42]. Moreover, the expression of neural specific proteins fails to characterize authentic functional and mature neurons, as some neural markers are already observed in primary cultures (without any differentiation protocols) [43,44] and even after mesenchymal differentiation [45].

Concerning preclinical cell therapy experiments on PD animals models, neural differentiation-based therapy protocols were performed using stem cells from Wharton's Jelly [46], dental pulp [47] and bone marrow [9,48] that underwent various culture conditions before being transplanted in 6-hydroxydopamine (6-OHDA)-treated rats. Behavioral and pathological enhancements were observed in most of the studies, but the underlying mechanisms were not sufficiently detailed, and no evidence for an appropriate integration into the lesioned central nervous system (CNS) was observed. Conversely, significant improvements were observed in PD animal models that were transplanted with BMSC without any pre-differentiation step. In those conditions, no sign of neural differentiation was properly observed. Still, beneficial effects and rescue of dopaminergic neurons were noticed and mainly associated with neuroprotection [49,50], trophic support (i.e. glial cell line-derived neurotrophic factor (GDNF) or epidermal growth factor (EGF) secretion) [50,51] or anti-inflammation (attenuation of blood-brain barrier damages, microglia inactivation) [52]. Moreover, BMSC graft induced proliferation and migration of endogenous SVZ neuroblasts in two PD animal models [50,53].

As regards the recent data about PD models and BMSC-based cell therapy, it appears that neural differentiation might not be responsible for physiopathological and clinical recoveries that are observed after BMSC transplantation in PD experimental models. Indeed, no evidence for *in vivo* functional neuronal replacement and CNS integration has been provided so far. Our results confirmed that bone marrow NCSC (in a pure population) are not any more competent than pure MSC nor whole BMSC to differentiate into neurons and integrate the damaged dopaminergic system. Altogether, it looks like adult BMSC are not a prime option for cell replacement therapies in the context of Parkinson's disease. However, neuroprotective, neurotrophic and anti-inflammatory features characterizing BMSC are of greater interest as regards CNS lesions management, and still need to be fully characterized [54].

Supporting Information

Figure S1 Survival rate of grafted cells at 3, 7, 14, 28 and 70 days after transplantation of MSC_{mix}/$NCSC_{mix}$ in MPTP and control mice. A. In MPTP mice, the number of surviving $NCSC_{mix}$ in the right striatum (*blue* X-gal staining and *purple* Hematoxylin-stained nuclei) can reach 15% in the first week after transplantation, then the cells begin to disappear and after 4 weeks, we only observe a mean survival rate of 1%. In control mice, even if the number of surviving cells is higher at 3 and 7 days post-graft, the survival rate also decreases to 1% after 28 days. **B.** MSC_{mix} (*green* CTG staining, *blue* DAPI-stained nuclei) seem to disappear more rapidly than $NCSC_{mix}$, since no cells were observed starting from 14 days, in both MPTP and control mice. **C.** Number of grafted cells that were recovered in mice brains at different delays post transplantation (Mean $\pm$ SEM) (CC = Corpus callosum; LV = Lateral ventricle; Scale bars = 500 μm).
(TIF)

Figure S2 Tyrosine hydroxylase staining of brain-injected $NCSC_{mix}$, at different delays post transplantation. Transplanted $NCSC_{mix}$ were detected by X-gal staining (*blue*). Grafted cells were negative for TH (*brown*) at 3, 7, 14 and 28 days after the cell injection ($n \geq 3$ for each group, at each delay post-transplantation). (Scale bars = 100 μm).
(TIF)

Acknowledgments

We thank Dr Angelo Abati and Marielle Delvoye from the Genetic Identification lab at the Forensic Institute of University of Liège for their great help in DNA extraction from brain slices. Finally, we would like to thanks Dr Ormenese and the GIGA plateform of flow cytometry and cell imaging for their valuable advises and technical support.

Author Contributions

Conceived and designed the experiments: VN SW-G BR. Analyzed the data: VN AM CC. Contributed reagents/materials/analysis tools: VN AMSW-G. Wrote the paper: VN SW-G BR.

References

1. Zhao C, Deng W, Gage FH (2008) Mechanisms and functional implications of adult neurogenesis. Cell, 132(4): 645–60.
2. Eriksson PS, Perfilieva E, Björk-Eriksson T, Alborn AM, Nordborg C, et al. (1998) Neurogenesis in the adult human hippocampus. Nat Med 4(11): 1313–7.
3. Hall PA, Watt FM (1989) Stem cells: the generation and maintenance of cellular diversity. Development 106 (4): 619–33.
4. Patani R, Hollins AJ, Wishart TM, Puddifoot CA, Álvarez S, et al. (2011) Retinoid-independent motor neurogenesis from human embryonic stem cells reveals a medial columnar ground state. Nat Commun 2: 214.
5. Kriks S, Shim JW, Piao J, Ganat Y, Wakeman DR, et al. (2011) Dopamine neurons derived from human ES cells efficiently engraft in animal models of Parkinson's disease. Nature 480(7378): 547–51.
6. Swistowski A, Peng J, Liu Q, Mali P, Rao MS, et al. (2010) Efficient generation of functional dopaminergic neurons from human induced pluripotent stem cells under defined conditions. Stem Cells 28(10): 1893–904.
7. Malgrange B, Borgs L, Grobarczyk B, Purnelle A, Ernst P, et al. (2011) Using human pluripotent stem cells to untangle neurodegenerative disease mechanisms. Cell Mol Life Sci, 68(4): 635–49.
8. Wislet-Gendebien S, Laudet E, Neirinckx V, Rogister B (2012) Adult bone marrow: which stem cells for cellular therapy protocols in neurodegenerative disorders? J Biomed Biotechnol 2012: 601560.
9. Khoo ML, Tao H, Meedeniya AC, Mackay-Sim A, Ma DD (2011) Transplantation of neuronal-primed human bone marrow mesenchymal stem cells in hemiparkinsonian rodents. PLoS One 6(5): 19025.
10. Alexanian AR, Fehlings MG, Zhang Z, Maiman DJ (2011) Transplanted neurally modified bone marrow-derived mesenchymal stem cells promote tissue protection and locomotor recovery in spinal cord injured rats. Neurorehabil Neural Repair 25(9): 873–80.
11. Levy YS, Bahat-Stroomza M, Barzilay R, Burshtein A, Bulvik S, et al. (2008) Regenerative effect of neural-induced human mesenchymal stromal cells in rat models of Parkinson's disease. Cytotherapy 10(4): 340–52.
12. Reyes M, Verfaillie CM (2001) Characterization of multipotent adult progenitor cells, a subpopulation of mesenchymal stem cells. Ann N Y Acad Sci 938: 231–3; discussion 233–5.
13. Verfaillie CM (2005) Multipotent adult progenitor cells: an update. Novartis Found Symp 265: 55–61; discussion 61–5, 92–7.
14. D'Ippolito G, Diabira S, Howard GA, Menei P, Roos BA, et al. (2004) Marrow-isolated adult multilineage inducible (MIAMI) cells, a unique population of postnatal young and old human cells with extensive expansion and differentiation potential. J Cell Sci 117: 2971–81.
15. D'Ippolito G, Howard GA, Roos BA, Schiller PC (2006) Isolation and characterization of marrow-isolated adult multilineage inducible (MIAMI) cells. Exp Hematol 34(11): 1608–10.
16. Pittenger MF, Mackay AM, Beck SC, Jaiswal RK, Douglas R, et al. (1999) Multilineage potential of adult human mesenchymal stem cells. Science 284(5411): 143–7.
17. Dennis JE, Charbord P (2002) Origin and differentiation of human and murine stroma. Stem Cells 20(3): 205–14.

18. Takashima Y, Era T, Nakao K, Kondo S, Kasuga M, et al. (2007) Neuroepithelial cells supply an initial transient wave of MSC differentiation. Cell 129(7): 1377–88.
19. Nagoshi N, Shibata S, Kubota Y, Nakamura M, Nagai Y, et al. (2008) Ontogeny and multipotency of neural crest-derived stem cells in mouse bone marrow, dorsal root ganglia, and whisker pad. Cell Stem Cell 2(4): 392–403.
20. Morikawa S, Mabuchi Y, Niibe K, Suzuki S, Nagoshi N, et al. (2009) Development of mesenchymal stem cells partially originate from the neural crest. Biochem Biophys Res Commun 379: 1114–1119.
21. Glejzer A, Laudet E, Leprince P, Hennuy B, Poulet C, et al. (2011) Wnt1 and BMP2: two factors recruiting multipotent neural crest progenitors isolated from adult bone marrow. Cell Mol Life Sci, 68(12): 2101–14.
22. Wislet-Gendebien S, Laudet E, Neirinckx V, Alix P, Leprince P, et al. (2012) Mesenchymal stem cells and neural crest stem cells from adult bone marrow: characterization of their surprising similarities and differences. Cell Mol Life Sciences 69(15): p.2593–608.
23. Jackson-Lewis V, Jakowec M, Burke RE, Przedborski S (1995) Time course and morphology of dopaminergic neuronal death caused by the neurotoxin 1-methyl-4-phenyl-1,2,3,6-tetrahydropyridine. Neurodegeneration 4(3): 257–69.
24. Jackson-Lewis V, Przedborski S (2007) Protocol for the MPTP mouse model of Parkinson's disease. Nat Protoc 2(1): 141–51.
25. Wislet-Gendebien S, Leprince P, Moonen G, Rogister B (2003) Regulation of neural markers nestin and GFAP expression by cultivated bone marrow stromal cells. Journal of Cell Science 116: 3295–3302.
26. Phani S, Gonye G, Iacovitti L (2010) VTA neurons show a potentially protective transcriptional response to MPTP. Brain Res 1343: 1–13.
27. Sanchez-Ramos J, Song S, Cardozo-Pelaez F, Hazzi C, Stedeford T, et al. (2000) Adult bone marrow stromal cells differentiate into neural cells in vitro. Exp Neurol 164(2): 247–56.
28. Woodbury D, Schwarz EJ, Prockop DJ, Black IB (2000) Adult rat and human bone marrow stromal cells differentiate into neurons. J Neurosci Res 61(4): 364–70.
29. Venkataramana NK, Pal R, Rao SAV, Naik AL, Jan M, et al. (2012) Bilateral transplantation of allogenic adult human bone marrow-derived mesenchymal stem cells into the subventricular zone of Parkinson's disease: a pilot clinical study. Stem Cells Int 2012: 931902.
30. Venkataramana NK, Kumar SK, Balaraju S, Radhakrishnan RC, Bansal A, et al. (2010) Open-labeled study of unilateral autologous bone-marrow-derived mesenchymal stem cell transplantation in Parkinson's disease. Transl Res 155(2): 62–70.
31. Przedborski S, Vila M (2003) The 1-methyl-4-phenyl-1,2,3,6-tetrahydropyridine mouse model: a tool to explore the pathogenesis of Parkinson's disease. Ann N Y Acad Sci 991: 189–98.
32. Przedborski S, Jackson-Lewis V, Djaldetti R, Liberatore G, Vila M, et al. (2000) The parkinsonian toxin MPTP: action and mechanism. Restor Neurol Neurosci 16(2): 135–142.
33. Lofrumento DD, Saponaro C, Cianciulli A, De Nuccio F, Mitolo V, et al. (2011) MPTP-induced neuroinflammation increases the expression of pro-inflammatory cytokines and their receptors in mouse brain. Neuroimmunomodulation 18(2): 79–88.
34. Hébert G, Arsaut J, Dantzer R, Demotes-Mainard J (2003) Time-course of the expression of inflammatory cytokines and matrix metalloproteinases in the striatum and mesencephalon of mice injected with 1-methyl-4-phenyl-1,2,3,6-tetrahydropyridine, a dopaminergic neurotoxin. Neurosci Lett 349(3): 191–5.
35. Ciesielska A, Joniec I, Przybylkowski A (2003) Dynamics of expression of the mRNA for cytokines and inducible nitric synthase in a murine model of the Parkinson's disease. Acta Neurobiol Exp (Wars) 63(2): 117–26.
36. Li Y, Chen J, Wang L, Zhang L, Lu M, Chopp M (2001) Intracerebral transplantation of bone marrow stromal cells in a 1-methyl-4-phenyl-1,2,3,6-tetrahydropyridine mouse model of Parkinson's disease. Neurosci Lett, 316(2): 67–70.
37. Gibrat C, Saint-Pierre M, Bousquet M, Lévesque D, Rouillard C, et al. (2009) Differences between subacute and chronic MPTP mice models: investigation of dopaminergic neuronal degeneration and alpha-synuclein inclusions. J Neurochem 109(5): 1469–82.
38. Trzaska KA, Kuzhikandathil EV, Rameshwar P (2007) Specification of a dopaminergic phenotype from adult human mesenchymal stem cells. Stem Cells 25(11): p.2797–808.
39. Tatard VM, D'Ippolito G, Diabira S, Valeyev A, Hackman J, et al. (2007) Neurotrophin-directed differentiation of human adult marrow stromal cells to dopaminergic-like neurons. Bone 40(2): 360–73.
40. Curtis KM, Gomez LA, Schiller PC (2012) Rac1b regulates NT3-stimulated Mek-Erk signaling, directing marrow-isolated adult multilineage inducible (MIAMI) cells toward an early neuronal phenotype. Mol Cell Neurosci 49(2): p.138–48.
41. Sanchez-Ramos JR (2002) Neural cells derived from adult bone marrow and umbilical cord blood. J Neurosci Res 69(6): 880–93.
42. Liu J, Song L, Jiang C, Liu Y, George J, et al. (2012) Electrophysiological properties and synaptic function of mesenchymal stem cells during neurogenic differentiation - a mini-review. Int J Artif Organs 35(5): 323–37.
43. Montzka K, Lassonczyk N, Tschöke B, Neuss S, Führmann T, et al. (2009) Neural differentiation potential of human bone marrow-derived mesenchymal stromal cells: misleading marker gene expression. BMC Neurosci 10: 16.
44. Tondreau T, Lagneaux L, Dejeneffe M, Massy M, Mortier C, et al (2004) Bone marrow-derived mesenchymal stem cells already express specific neural proteins before any differentiation. Differentiation 72(7): 319–26.
45. Foudah D, Redondo J, Tredici G, Miloso M (2011) Evaluation of neural markers expression in human mesenchymal stem cells after mesengenic differentiation. Italian Journal of Anatomy and Embryology 16(1).
46. Fu YS, Cheng YC, Lin MY, Cheng H, Chu PM, et al (2006) Conversion of human umbilical cord mesenchymal stem cells in Wharton's jelly to dopaminergic neurons in vitro: potential therapeutic application for Parkinsonism. Stem Cells 24(1): 115–24.
47. Wang J, Wang X, Sun Z, Wang X, Yang H, et al. (2010) Stem cells from human-exfoliated deciduous teeth can differentiate into dopaminergic neuron-like cells. Stem Cells Dev 19(9): 1375–83.
48. Offen D, Barhum Y, Levy YS, Burshtein A, Panet H, et al. (2007) Intrastriatal transplantation of mouse bone marrow-derived stem cells improves motor behavior in a mouse model of Parkinson's disease. J Neural Transm Suppl 72: 133–43.
49. Park HJ, Bang G, Lee BR, Kim HO, Lee PH (2011) Neuroprotective effect of human mesenchymal stem cells in an animal model of double toxin-induced multiple system atrophy parkinsonism. Cell Transplant 20(6): 827–35.
50. Park HW, Cho JS, Park CK, Jung SJ, Park CH, et al. (2012) Mesenchymal stem cells augment neurogenesis in the subventricular zone and enhance differentiation of neural precursor cells into dopaminergic neurons in the substantia nigra of a Parkinsonian model. Cell Transplant 7(4)
51. Blandini F, Cova L, Armentero MT, Zennaro E, Levandis G, et al. (2010) Transplantation of undifferentiated human mesenchymal stem cells protects against 6-hydroxydopamine neurotoxicity in the rat. Cell Transplant 19(2): 203–17.
52. Chao YX, He BP, Tay SS (2009) Mesenchymal stem cell transplantation attenuates blood brain barrier damage and neuroinflammation and protects dopaminergic neurons against MPTP toxicity in the substantia nigra in a model of Parkinson's disease. J Neuroimmunol 216(1–2): 39–50.
53. Cova L, Armentero MT, Zennaro E, Calzarossa C, Bossolasco P, et al. (2010) Multiple neurogenic and neurorescue effects of human mesenchymal stem cell after transplantation in an experimental model of Parkinson's disease. Brain Res 1311: 12–27.
54. Neirinckx V, Coste C, Rogister B, Wislet-Gendebien S (2013) Concise review: adult mesenchymal stem cells, adult neural crest stem cells, and therapy of neurological pathologies: a state of play. Stem Cells Transl Med 2(4): 284–96.
